# Supplementary material for: Acylpyruvates and Their Heterocyclic Derivatives as Growth Regulators in Chlorella vulgaris
Source: BioTech (Basel). 2025 Nov 10;14(4):90. doi: 10.3390/biotech14040090 (PMC12641706; doi:10.3390/biotech14040090)
Supplement: Supplementary file 1 [file biotech-14-00090-s001.zip › biotech-3899787-supplementary/SI/spectra.pdf]

Supporting information

for

Acylpyruvates and Their Heterocyclic Derivatives as Growth Regulators in *Chlorella vulgaris*

Anastasia D. Novokshonova [1,2], Pavel V. Khramtsov [2,3,*], Maksim V. Dmitriev [1] and Ekaterina E. Khramtsova [1,*]

[1] Department of Chemistry, Perm State University, ul. Bukireva, 15, 614990 Perm, Russia, caterina.stepanova@psu.ru

[2] Institute of Ecology and Genetics of Microorganisms, Perm Federal Research Center, The Ural Branch of Russian Academy of Sciences, ul. Goleva, 13, 614081 Perm, Russia

[3] Department of Biology, Perm State University, ul. Bukireva, 15, 614990 Perm, Russia

* Correspondence: khramtsovpavel@yandex.ru (P.V.K.), caterina.stepanova@psu.ru (E.E.K.)

Table of contents

NMR charts of compounds **2a-f,j-n**……………………………….S3

NMR charts of compounds **3a-i**………..……………………….S15

NMR chart of compound **4**……………………….……………S25

NMR charts of compounds **6a-g**…………….…………………S26

NMR charts of compounds **7a-d**…………….…………………S34

NMR chart of compound **8**………………..……………………S39

NMR charts of compounds **10a-q**………..…………………….S40

NMR chart of compound **11**……………………….……………S58

NMR chart of compound **13a**…………...……………………….S59

NMR charts of compound **14**………..………………………….S60

NMR chart of compound **15**…………………………..…………S62

NMR chart of compound **16**…………………………..…………S63

ORTEP images of X-ray crystal structures **2n, 6f, 14**…...……...S64

NMR charts of compounds 2a-f,j-n

Acquisition Time (sec)	2.0447						
				Frequency (MHz)	400.17	***Nucleus***	1H
Number of Transients	8	***Origin***	spect	***Original Points Count***	16384	***Owner***	nmr
Points Count	16384	***Pulse Sequence***	zg	***Receiver Gain***	37.99	***SW(cyclical) (Hz)***	8012.82
Solvent	CHLOROFORM-d	***Spectrum Offset (Hz)***	2461.4089	***Spectrum Type***	STANDARD	***Sweep Width (Hz)***	8012.33
Temperature (degree C)	39.997						

MAN7738001.esp

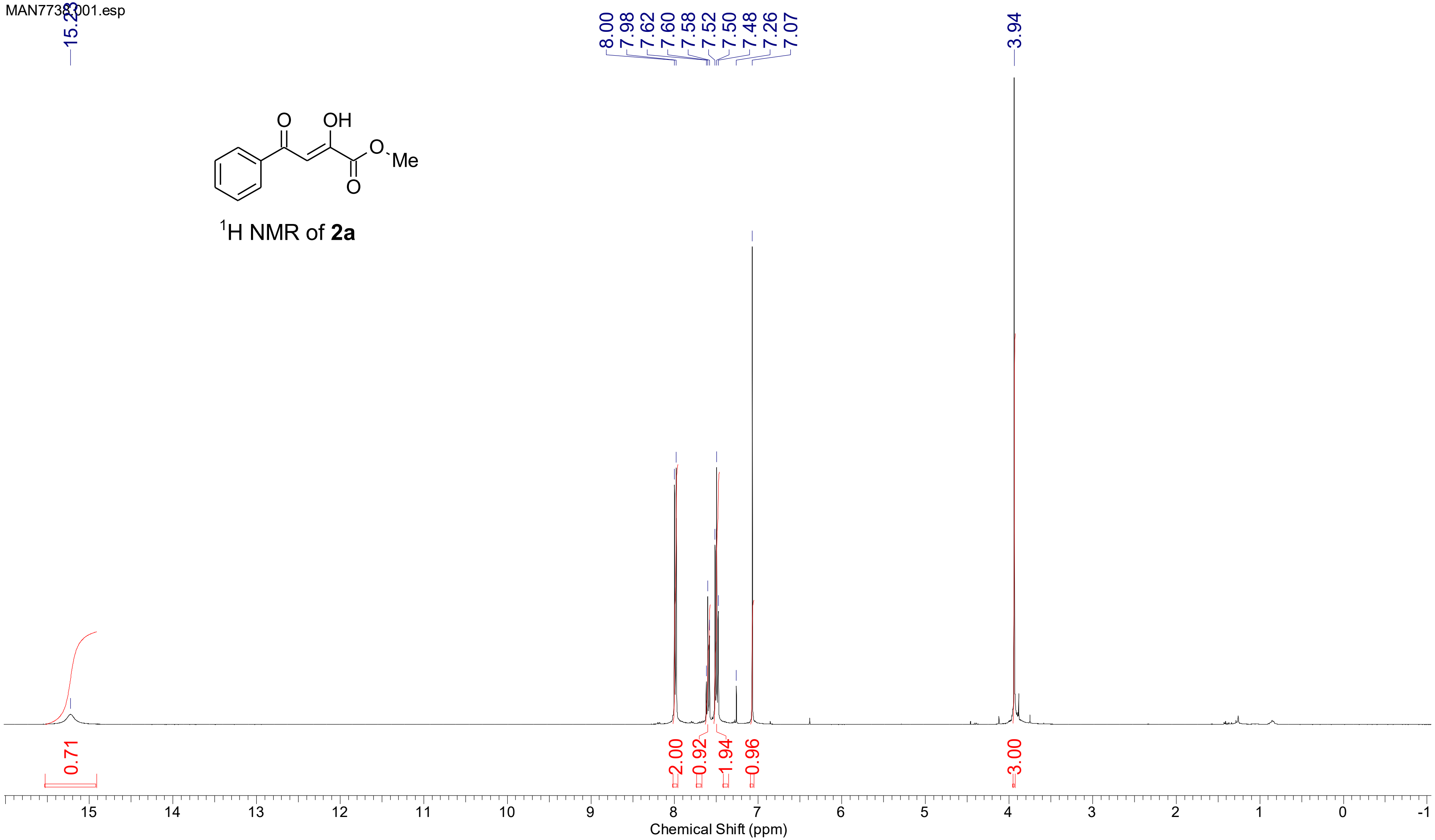

Acquisition Time (sec)	2.0447								
						Frequency (MHz)	400.17	***Nucleus***	1H
Number of Transients	8	***Origin***	spect	***Original Points Count***	16384	***Owner***	nmr	***Points Count***	16384
Pulse Sequence	zg	***Receiver Gain***	37.99	***SW(cyclical) (Hz)***	8012.82	***Solvent***	CHLOROFORM-d		
Spectrum Offset (Hz)	2461.4089	***Spectrum Type***	STANDARD	***Sweep Width (Hz)***	8012.33	***Temperature (degree C)***	39.998		

MAN9912.001.esp

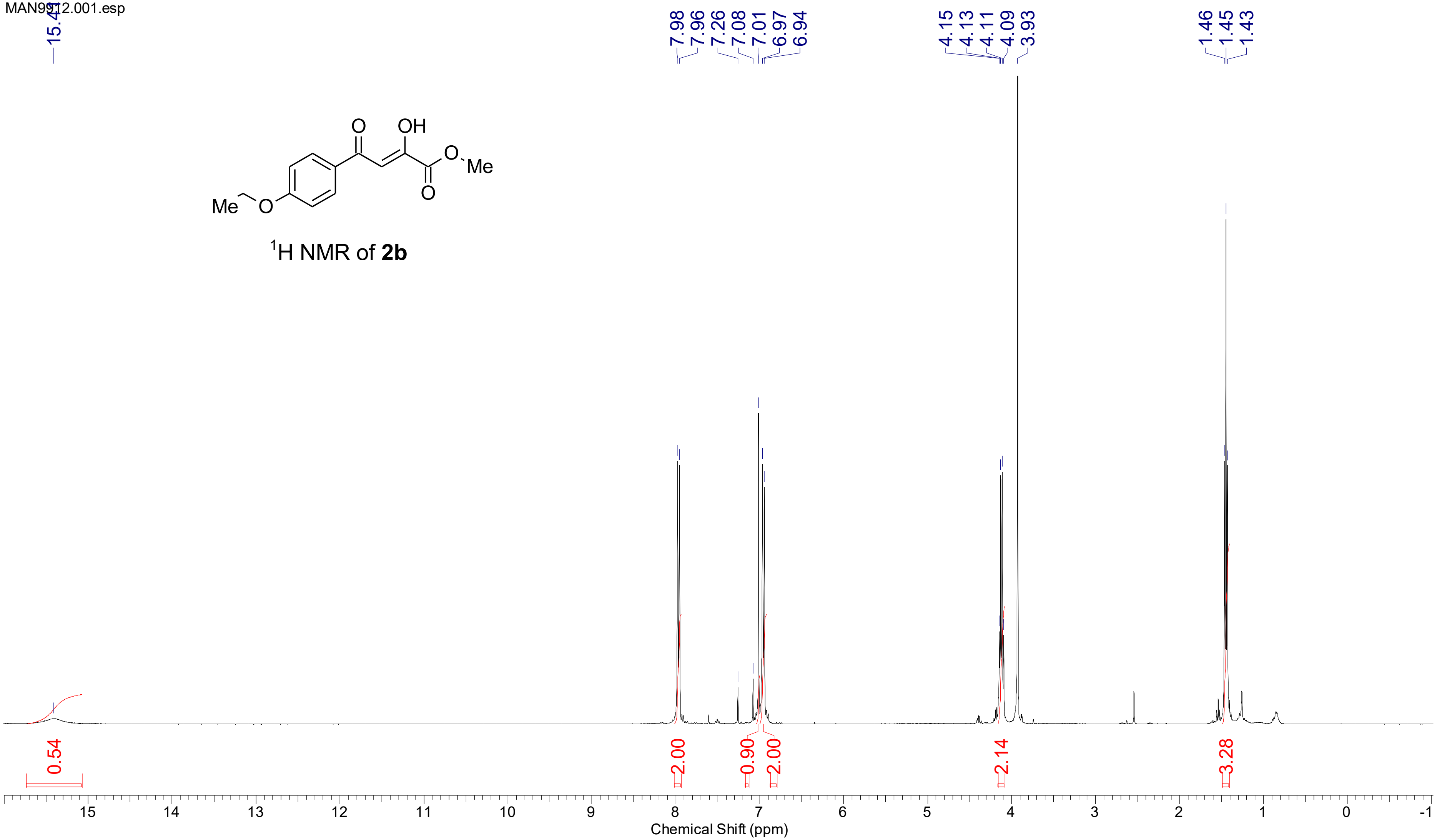

^{1}H NMR of **2b**

Acquisition Time (sec)	1.2976						
						Frequency (MHz)	100.62
Number of Transients	512	*Origin*	spect	*Original Points Count*	32768	*Owner*	nmr
Pulse Sequence	zgpg30	*Receiver Gain*	196.95	*SW(cyclical) (Hz)*	25252.53	*Solvent*	CHLOROFORM-d
Spectrum Offset (Hz)	11572.2871	*Spectrum Type*	STANDARD	*Sweep Width (Hz)*	25251.75	*Temperature (degree C)*	39.995

Nucleus	13C
Points Count	32768

MAN9912002.esp

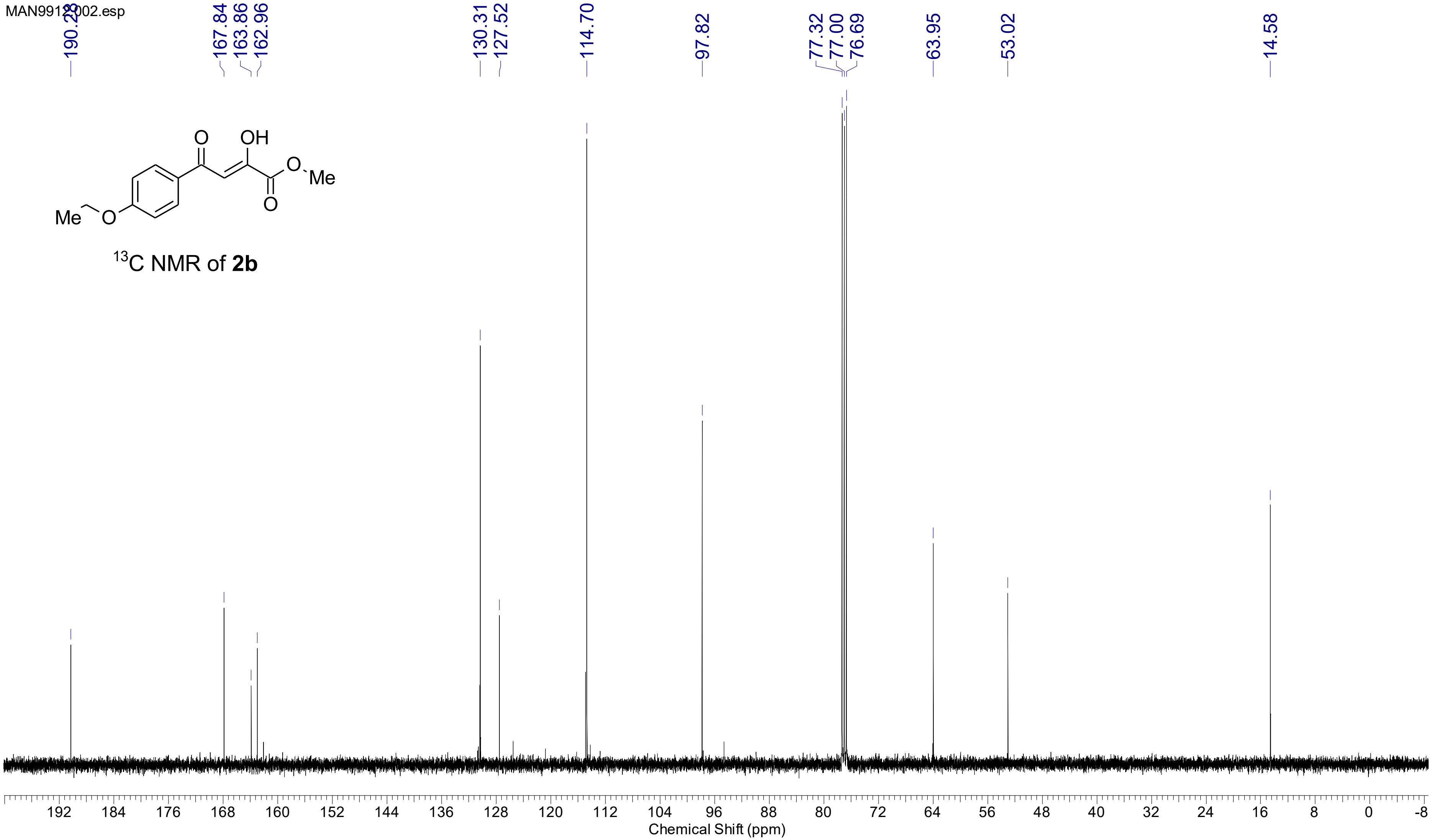

^{13}C NMR of **2b**

Acquisition Time (sec)	2.0447								
						Frequency (MHz)	400.17	***Nucleus***	1H
Number of Transients	8	***Origin***	spect	***Original Points Count***	16384	***Owner***	nmr	***Points Count***	16384
Pulse Sequence	zg	***Receiver Gain***	37.99	***SW(cyclical) (Hz)***	8012.82	***Solvent***	CHLOROFORM-d		
Spectrum Offset (Hz)	2461.4089	***Spectrum Type***	STANDARD	***Sweep Width (Hz)***	8012.33	***Temperature (degree C)***	39.996		

MAN8139.004.esp

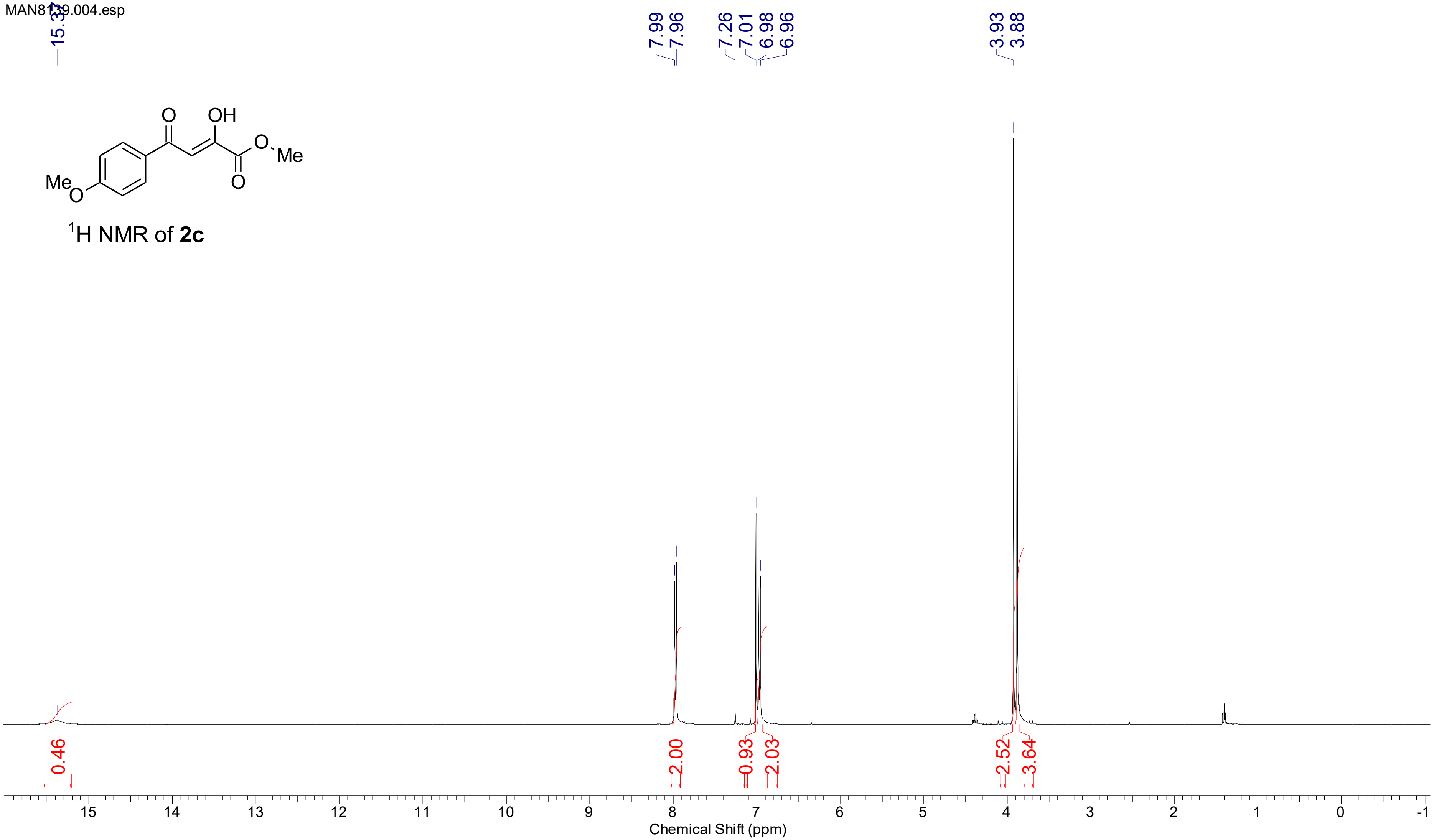

^{1}H NMR of **2c**

Acquisition Time (sec)	2.0447								
						Frequency (MHz)	400.17	***Nucleus***	1H
Number of Transients	8	***Origin***	spect	***Original Points Count***	16384	***Owner***	nmr	***Points Count***	16384
Pulse Sequence	zg	***Receiver Gain***	109.22	***SW(cyclical) (Hz)***	8012.82	***Solvent***	CHLOROFORM-d		
Spectrum Offset (Hz)	2461.4089	***Spectrum Type***	STANDARD	***Sweep Width (Hz)***	8012.33	***Temperature (degree C)***	40.001		

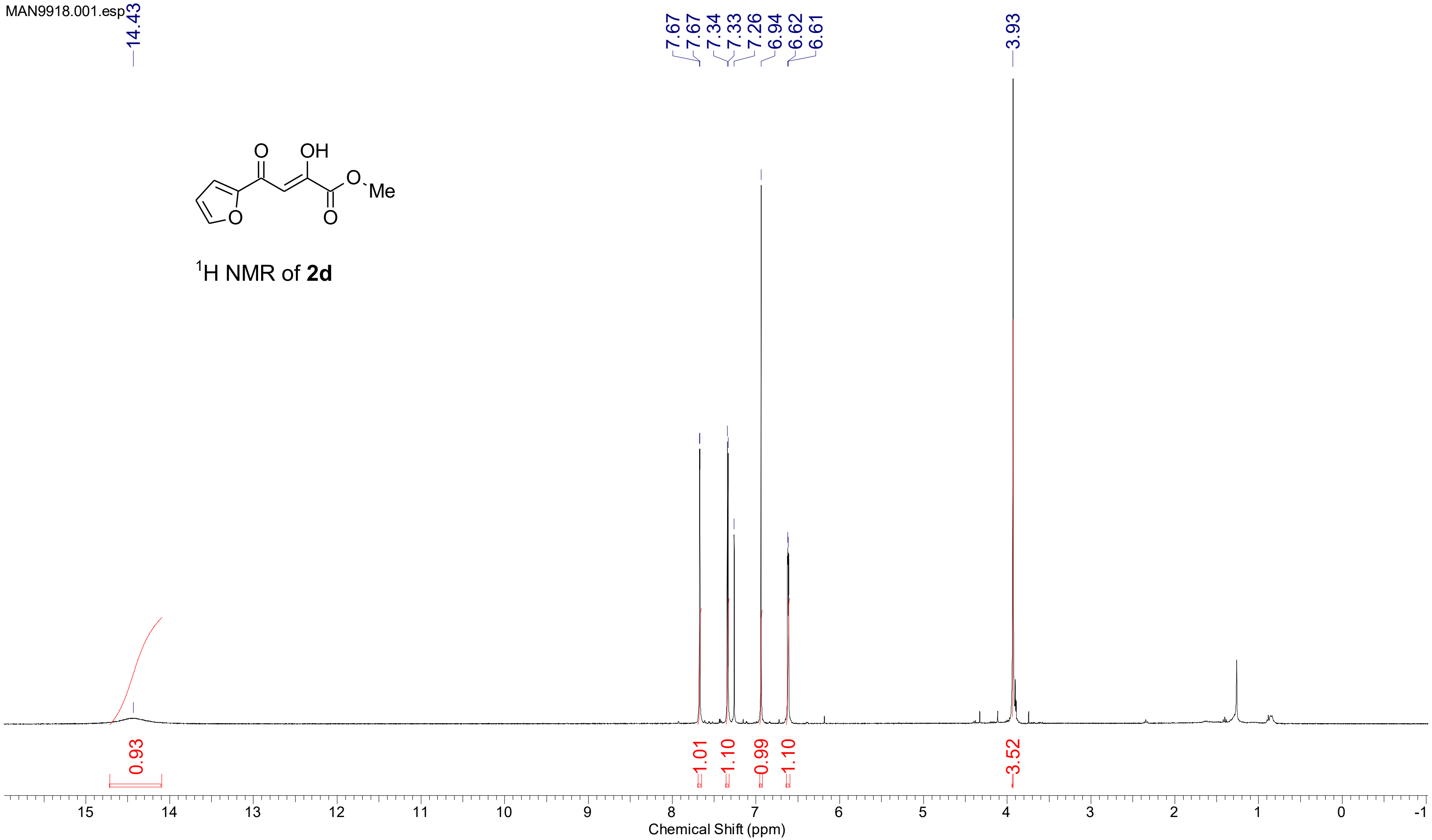

^{1}H NMR of **2d**

Acquisition Time (sec)	2.0447								
						Frequency (MHz)	400.17	***Nucleus***	1H
Number of Transients	8	***Origin***	spect	***Original Points Count***	16384	***Owner***	nmr	***Points Count***	16384
Pulse Sequence	zg	***Receiver Gain***	122.56	***SW(cyclical) (Hz)***	8012.82	***Solvent***	CHLOROFORM-d		
Spectrum Offset (Hz)	2461.8982	***Spectrum Type***	STANDARD	***Sweep Width (Hz)***	8012.33	***Temperature (degree C)***	39.999		

MAN9919.001.esp

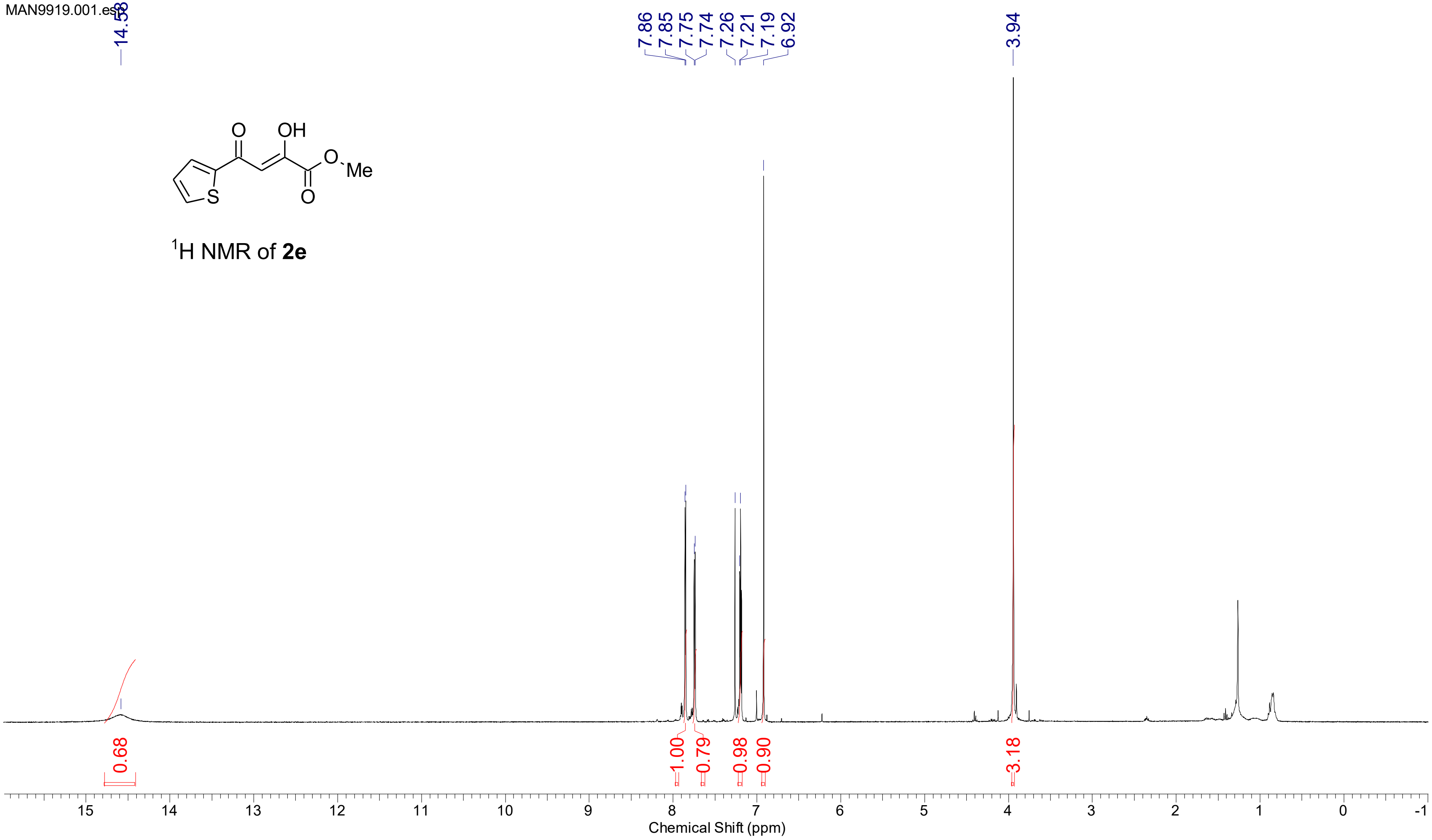

Acquisition Time (sec)	2.0447								
						Frequency (MHz)	400.17	***Nucleus***	1H
Number of Transients	8	***Origin***	spect	***Original Points Count***	16384	***Owner***	nmr	***Points Count***	16384
Pulse Sequence	zg	***Receiver Gain***	77.64	***SW(cyclical) (Hz)***	8012.82	***Solvent***	CHLOROFORM-d		
Spectrum Offset (Hz)	2461.8982	***Spectrum Type***	STANDARD	***Sweep Width (Hz)***	8012.33	***Temperature (degree C)***	40.001		

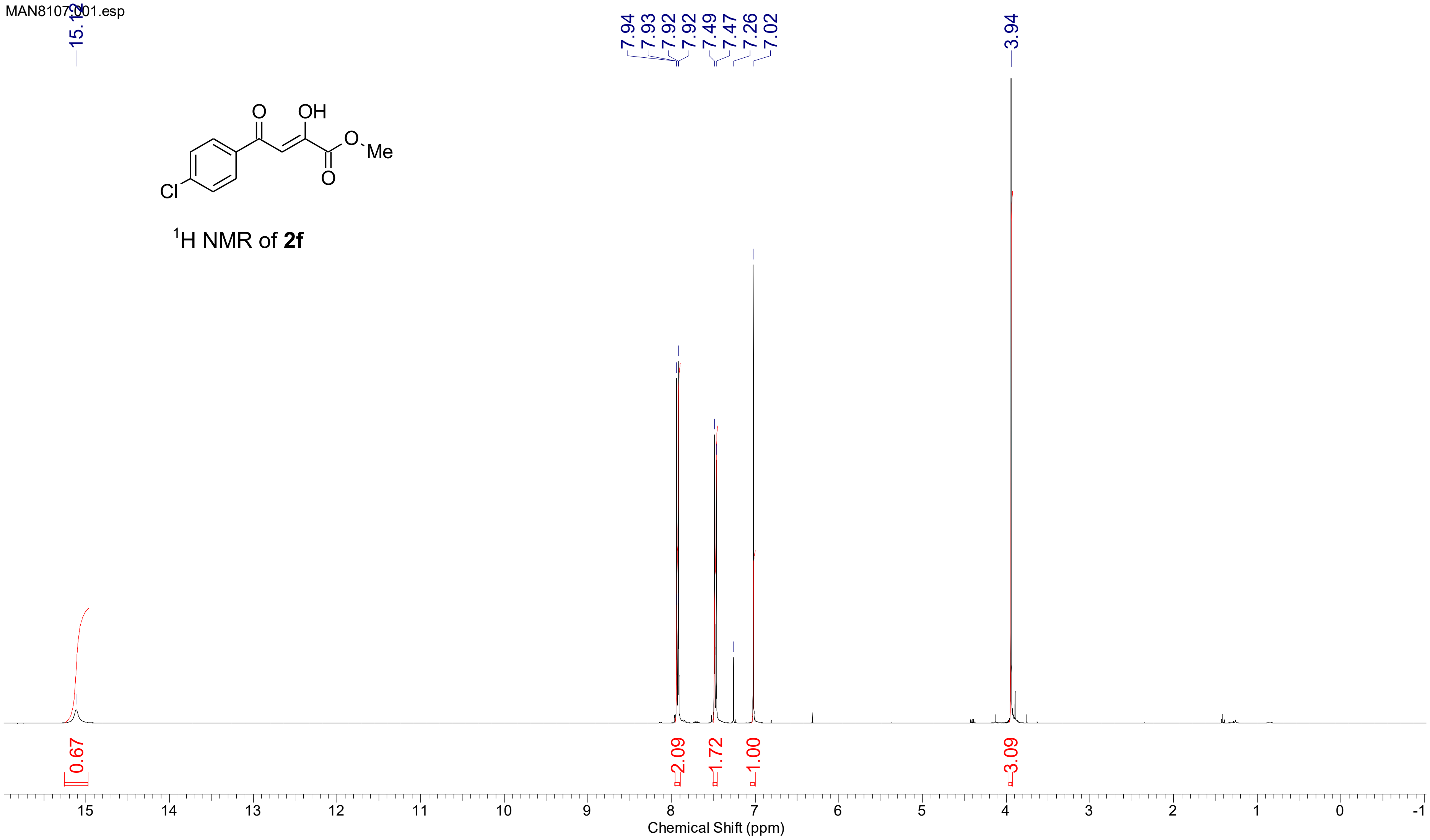

Acquisition Time (sec)	2.0447	Comment	MAN9925 1H				
Frequency (MHz)	400.17	Nucleus	1H	Number of Transients	8	Origin	spect	Original Points Count	16384
Owner	nmr	Points Count	16384	Pulse Sequence	zg	Receiver Gain	109.22	SW(cyclical) (Hz)	8012.82
Solvent	CHLOROFORM-d			Spectrum Offset (Hz)	2461.4089	Spectrum Type	STANDARD	Sweep Width (Hz)	8012.33
Temperature (degree C)	39.993								

MAN9925001.esp

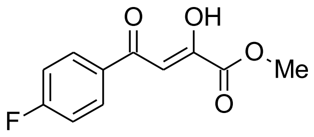

^{1}H NMR of **2j**

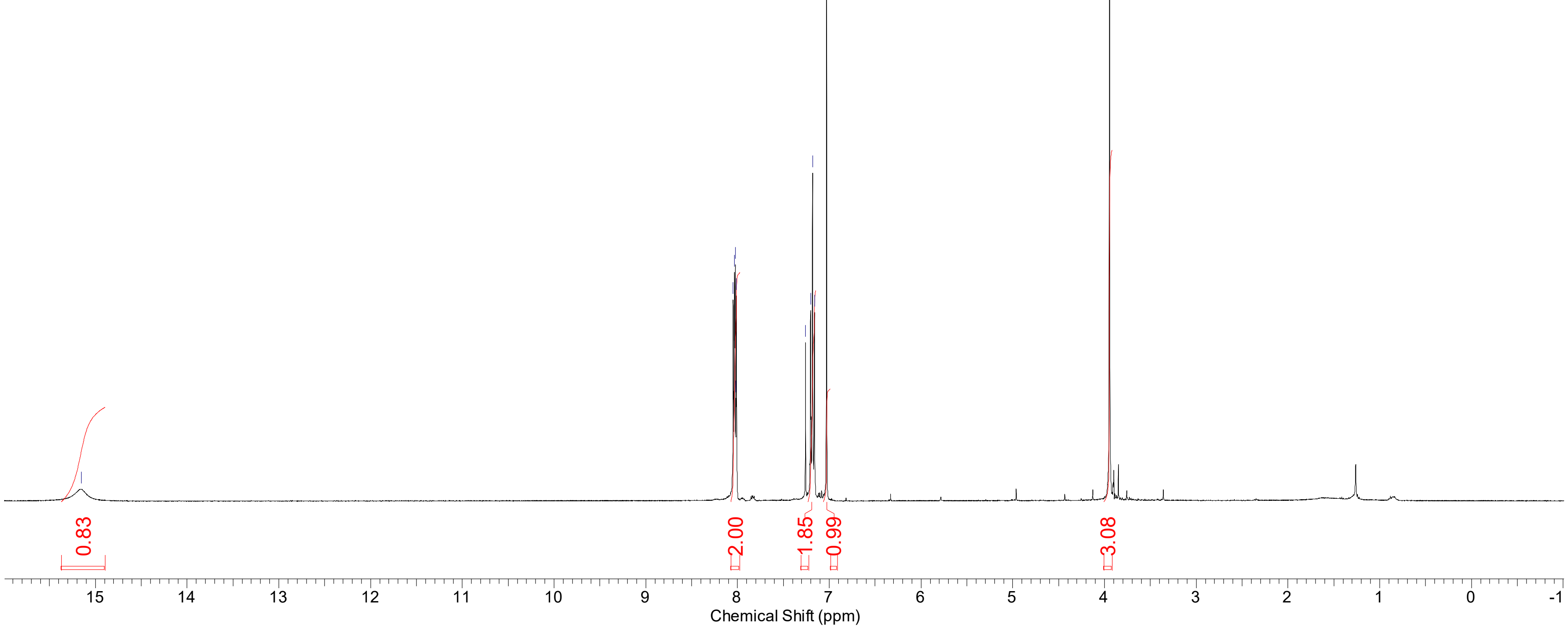

Acquisition Time (sec)	2.0447	Comment	MAN9633 1H						
Frequency (MHz)	400.17	Nucleus	1H	Number of Transients	8	Origin	spect	Original Points Count	16384
Owner	nmr	Points Count	16384	Pulse Sequence	zg	Receiver Gain	37.99	SW(cyclical) (Hz)	8012.82
Solvent	CHLOROFORM-d			Spectrum Offset (Hz)	2461.4089	Spectrum Type	STANDARD	Sweep Width (Hz)	8012.33
Temperature (degree C)	39.999								

MAN9633.001.esp

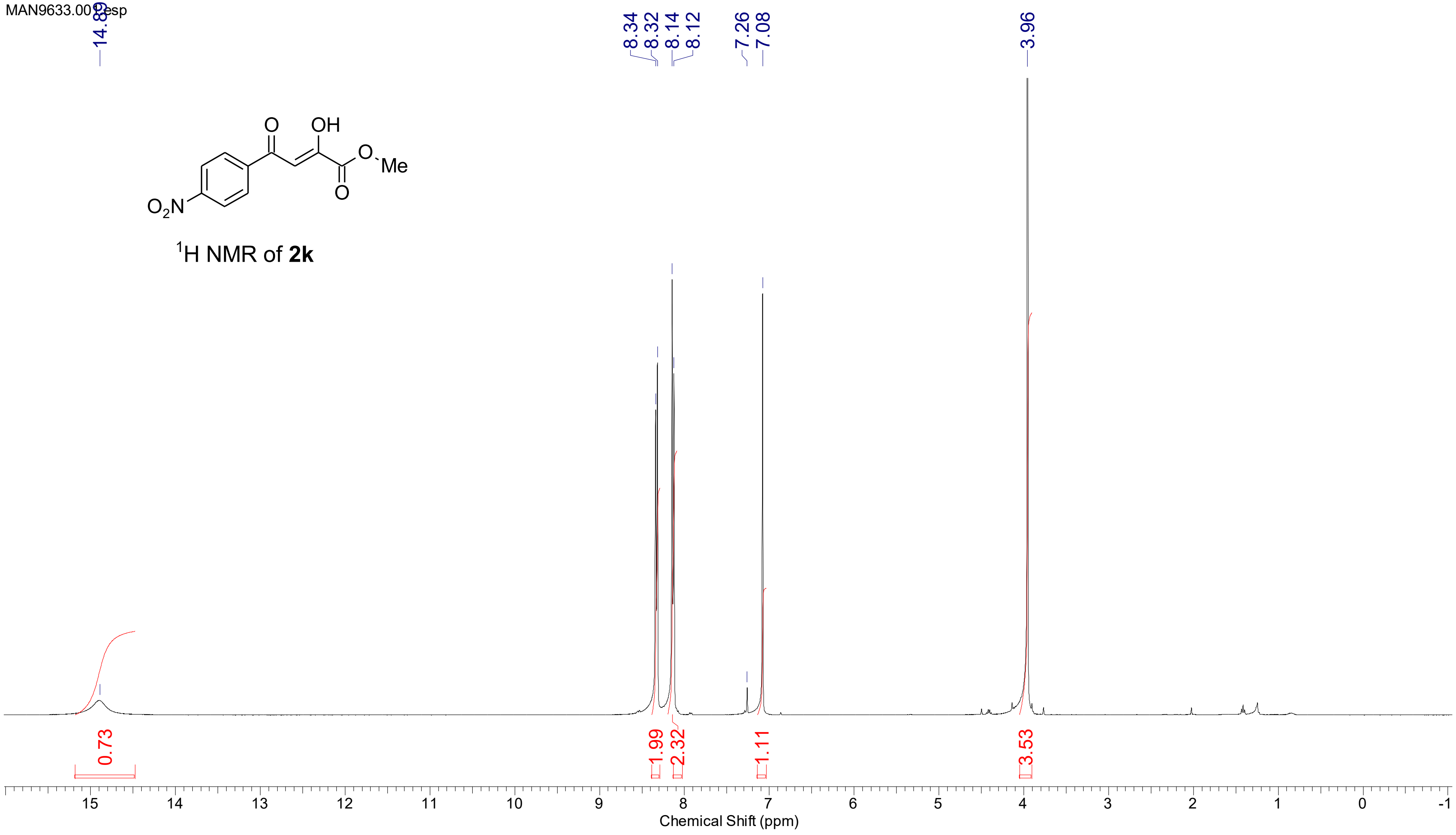

^{1}H NMR of **2k**

Acquisition Time (sec)	2.0447						
				Frequency (MHz)	400.17	Nucleus	1H
Number of Transients	8	Origin	spect	Original Points Count	16384	Owner	nmr
Points Count	16384						
Pulse Sequence	zg	Receiver Gain	109.22	SW(cyclical) (Hz)	8012.82	Solvent	CHLOROFORM-d
Spectrum Offset (Hz)	2461.4089	Spectrum Type	STANDARD	Sweep Width (Hz)	8012.33	Temperature (degree C)	39.999

MAN9916.001.esp

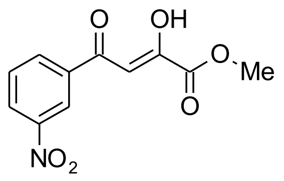

^{1}H NMR of **2l**

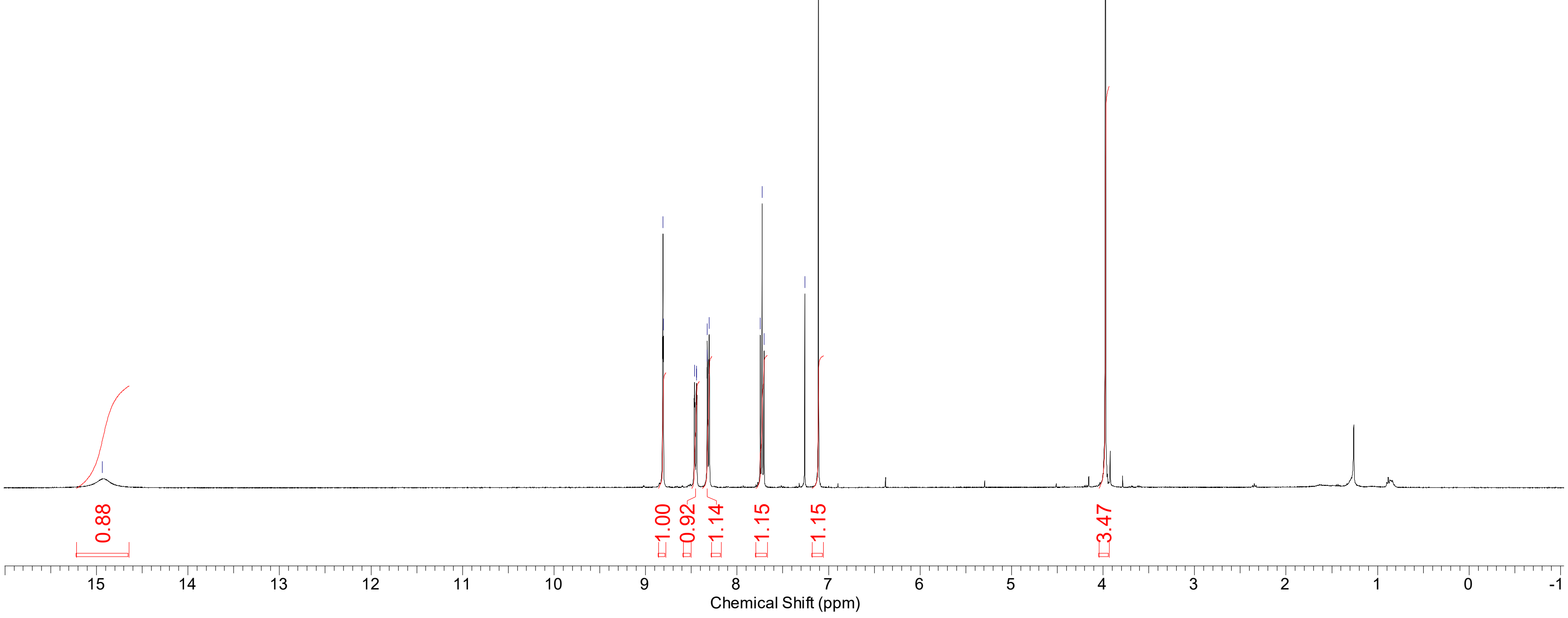

**Acquisition Time (sec)**	2.9999	**Comment**	MAN4874 1H						
**Frequency (MHz)**	400.17	**Nucleus**	1H	**Number of Transients**	32	**Origin**	spect	**Original Points Count**	24038
**Owner**	nmr	**Points Count**	32768	**Pulse Sequence**	zg	**Receiver Gain**	109.22	**SW(cyclical) (Hz)**	8012.82
**Solvent**	CHLOROFORM-d			**Spectrum Offset (Hz)**	2461.7759	**Spectrum Type**	STANDARD	**Sweep Width (Hz)**	8012.58
**Temperature (degree C)**	40.000								

MAN4874.001.esp

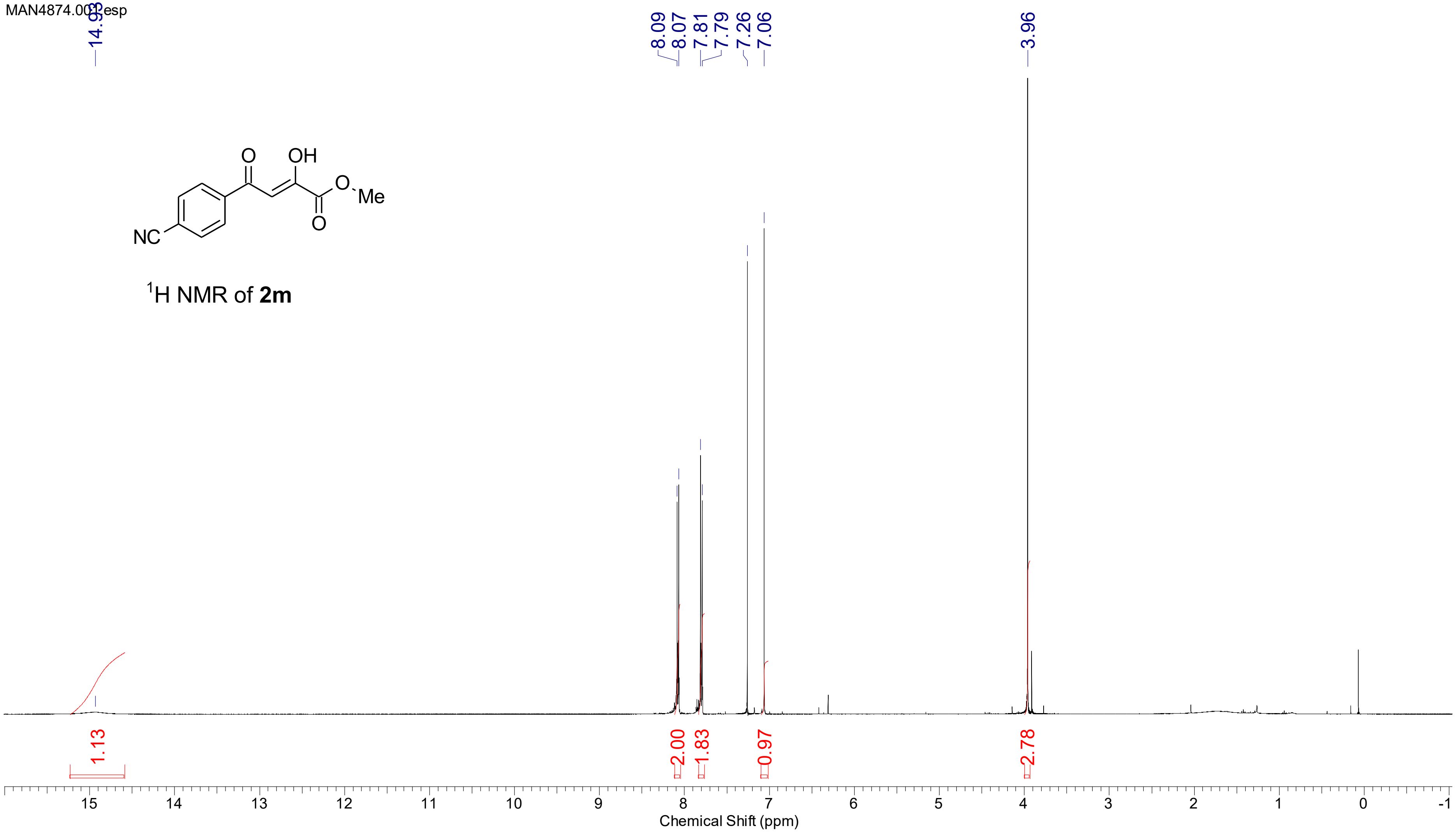

^{1}H NMR of **2m**

Acquisition Time (sec)	2.0447								
						Frequency (MHz)	400.17	***Nucleus***	1H
Number of Transients	8	***Origin***	spect	***Original Points Count***	16384	***Owner***	nmr	***Points Count***	16384
Pulse Sequence	zg	***Receiver Gain***	37.99	***SW(cyclical) (Hz)***	8012.82	***Solvent***	CHLOROFORM-d		
Spectrum Offset (Hz)	2461.4089	***Spectrum Type***	STANDARD	***Sweep Width (Hz)***	8012.33	***Temperature (degree C)***	39.999		

MAN7914.004.esp

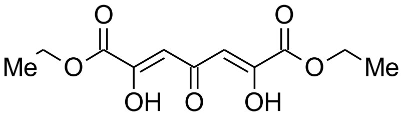

^{1}H NMR of **2n**

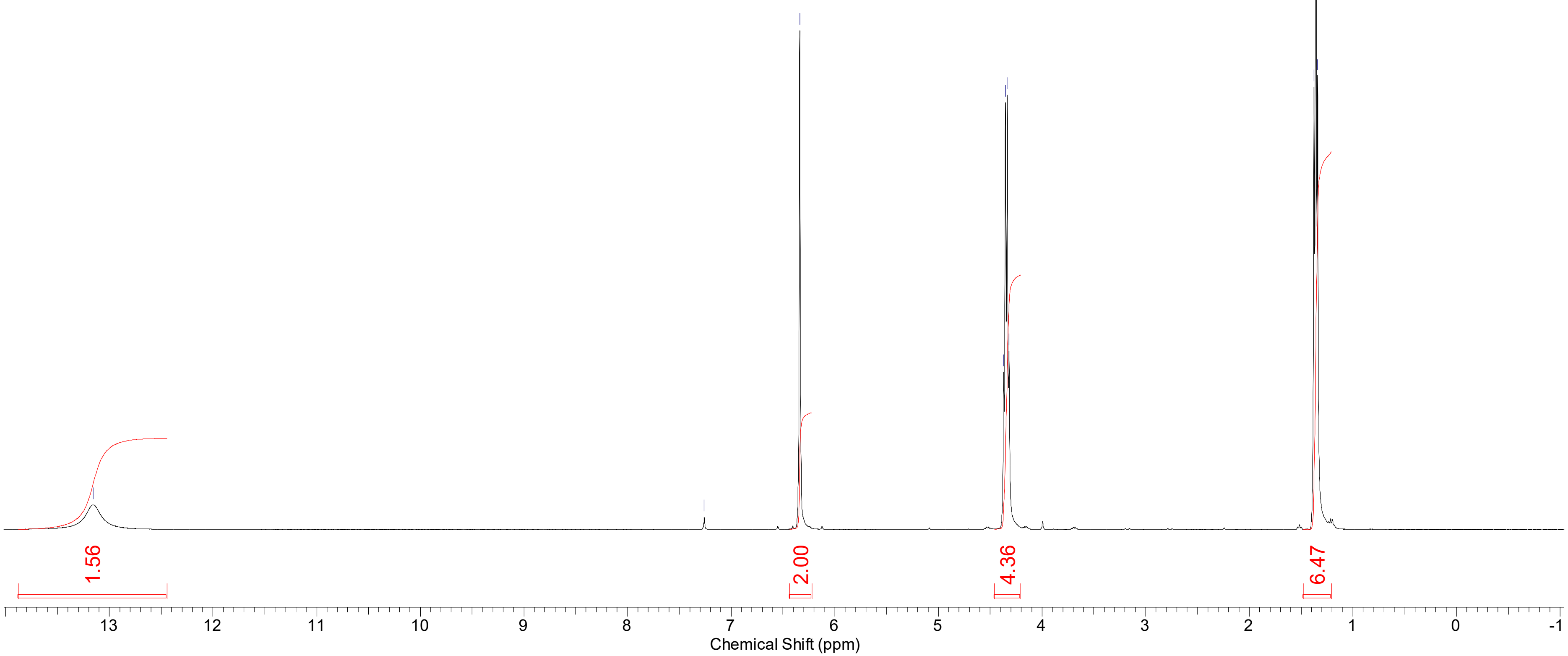

NMR charts of compounds 3a-i

Acquisition Time (sec)	2.0447	Comment	MAN7737 1H NS 32 CDCl3				
Frequency (MHz)	400.17	Nucleus	1H	Number of Transients	32	Origin	spect
Original Points Count	16384	Owner	nmr	Points Count	16384	Pulse Sequence	zg
Receiver Gain	95.56	SW(cyclical) (Hz)	8012.82	Solvent	CHLOROFORM-d		
Spectrum Offset (Hz)	2461.4089	Spectrum Type	STANDARD	Sweep Width (Hz)	8012.33	Temperature (degree C)	40.024

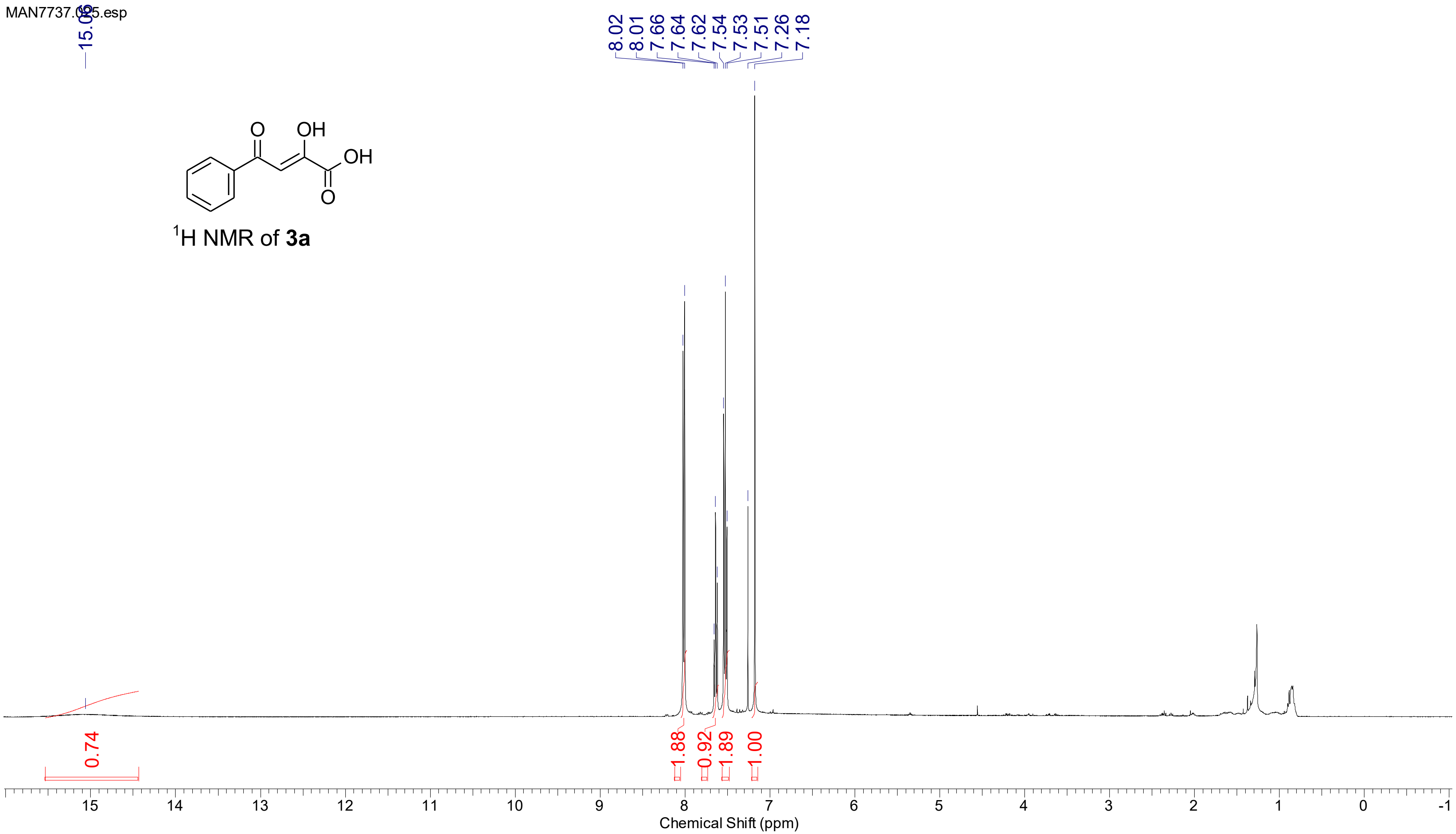

Acquisition Time (sec)	2.0447						
				Frequency (MHz)	400.17	Nucleus	1H
Number of Transients	8	Origin	spect	Original Points Count	16384	Owner	nmr	Points Count	16384
Pulse Sequence	zg	Receiver Gain	95.56	SW(cyclical) (Hz)	8012.82	Solvent	CHLOROFORM-d
Spectrum Offset (Hz)	2461.4089	Spectrum Type	STANDARD	Sweep Width (Hz)	8012.33	Temperature (degree C)	39.998

MAN9913.001.esp

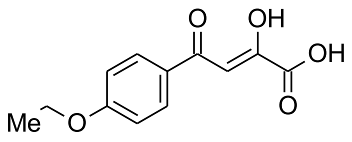

^{1}H NMR of **3b**

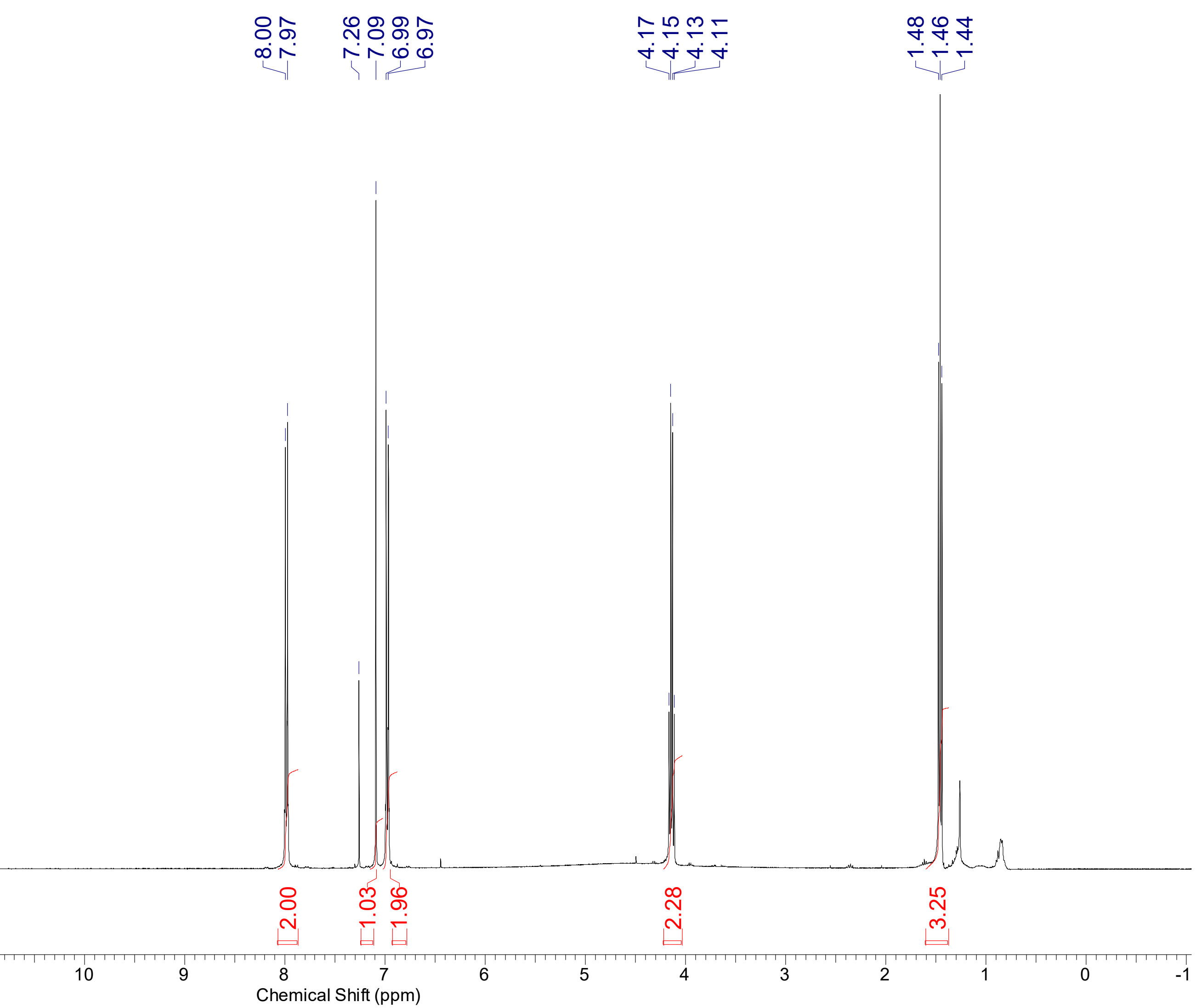

Acquisition Time (sec)	1.2976						
						Frequency (MHz)	100.62
Nucleus	13C	***Number of Transients***	512	***Origin***	spect	***Original Points Count***	32768
Owner	nmr	***Points Count***	1048576	***Pulse Sequence***	zgpg30	***Receiver Gain***	196.95
SW(cyclical) (Hz)	25252.53	***Solvent***	CHLOROFORM-d			***Spectrum Offset (Hz)***	11574.0342
Spectrum Type	STANDARD	***Sweep Width (Hz)***	25252.50	***Temperature (degree C)***	40.000		

MAN9913.002.esp

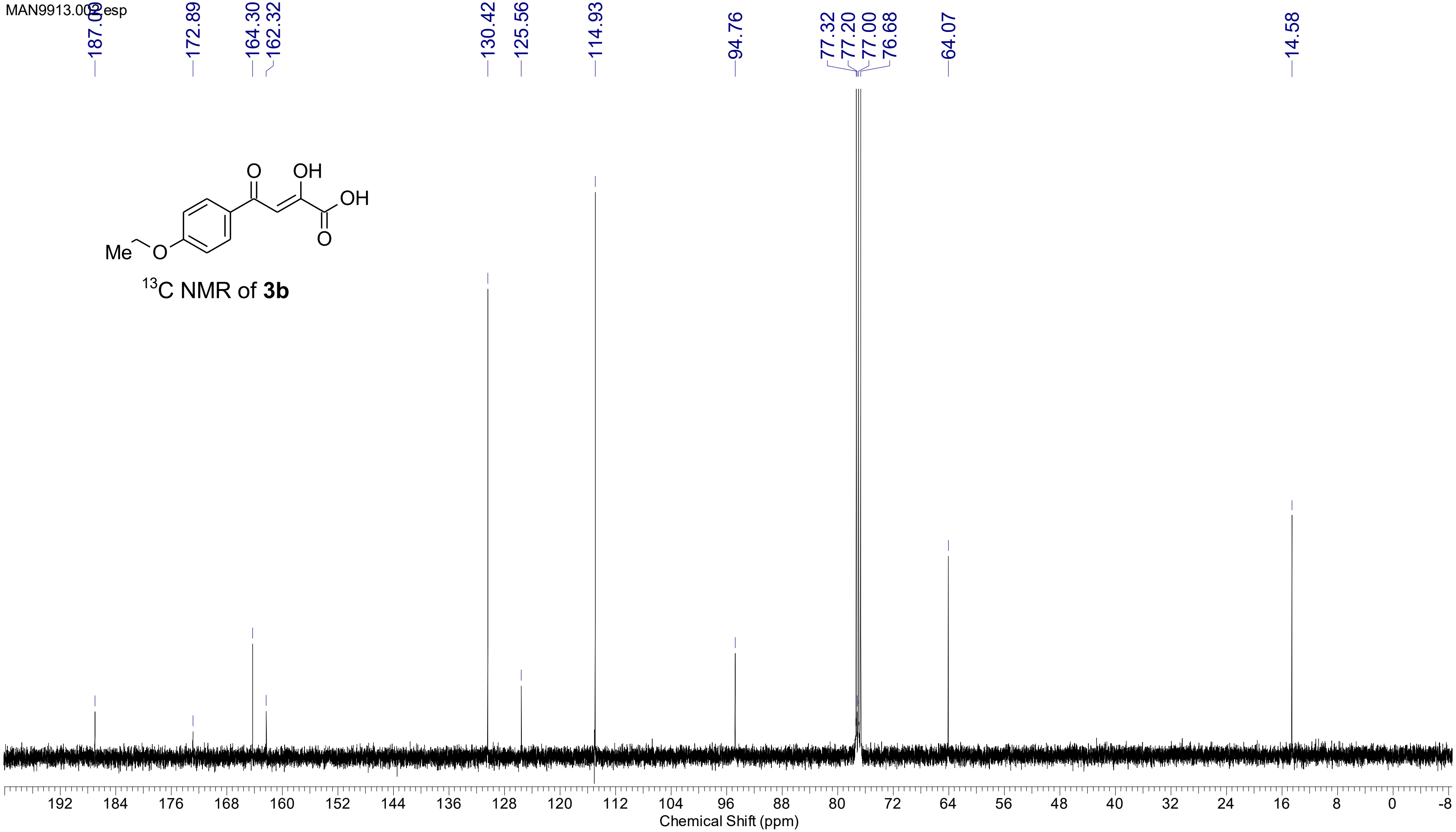

^{13}C NMR of **3b**

Acquisition Time (sec)	2.0447								
						Frequency (MHz)	400.17	*Nucleus*	1H
Number of Transients	8	*Origin*	spect	*Original Points Count*	16384	*Owner*	nmr	*Points Count*	16384
Pulse Sequence	zg	*Receiver Gain*	122.56	*SW(cyclical) (Hz)*	8012.82	*Solvent*	CHLOROFORM-d		
Spectrum Offset (Hz)	2461.4089	*Spectrum Type*	STANDARD	*Sweep Width (Hz)*	8012.33	*Temperature (degree C)*	39.998		

MAN9211.003.esp

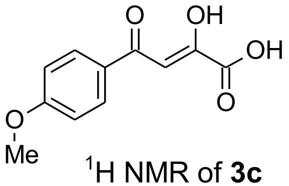

^{1}H NMR of **3c**

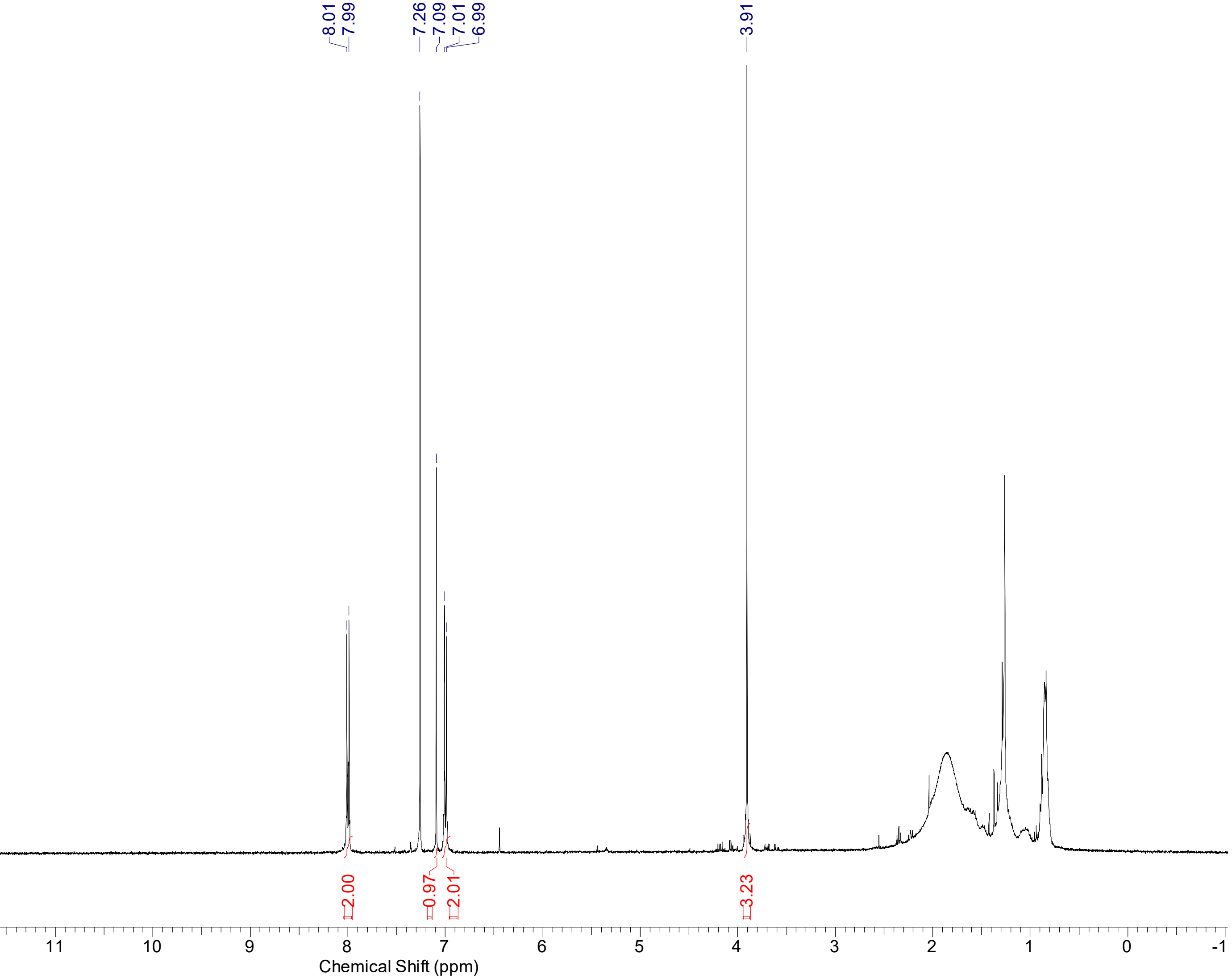

Acquisition Time (sec)	2.0447								
						Frequency (MHz)	400.17	Nucleus	1H
Number of Transients	8	Origin	spect	Original Points Count	16384	Owner	nmr	Points Count	16384
Pulse Sequence	zg	Receiver Gain	138.37	SW(cyclical) (Hz)	8012.82	Solvent	CHLOROFORM-d		
Spectrum Offset (Hz)	2461.8982	Spectrum Type	STANDARD	Sweep Width (Hz)	8012.33	Temperature (degree C)	40.000		

MAN9917.001.esp

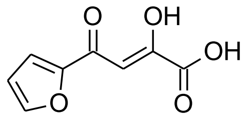

^{1}H NMR of **3d**

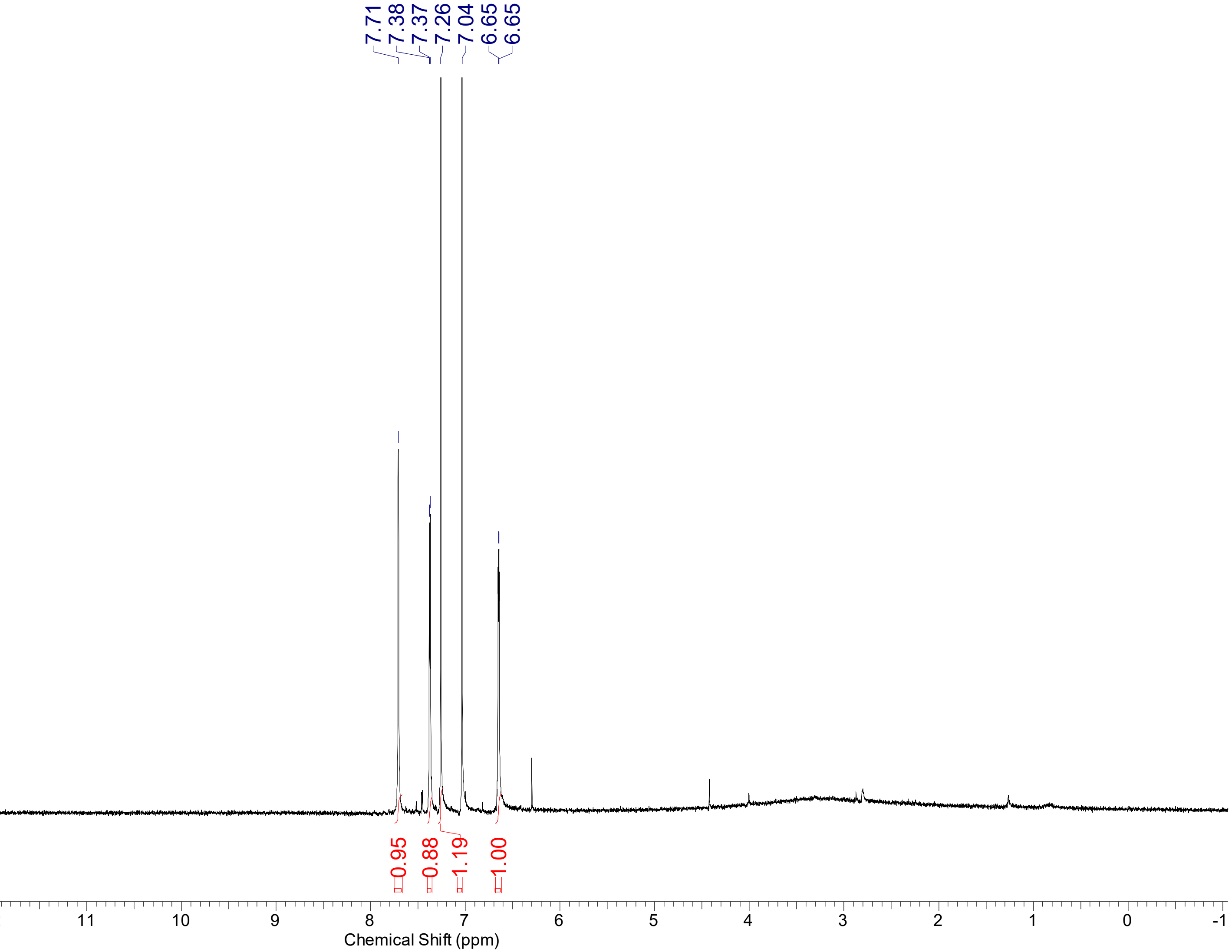

Acquisition Time (sec)	2.0447						
				Frequency (MHz)	400.17	*Nucleus*	1H
Number of Transients	8	*Origin*	spect	*Original Points Count*	16384	*Owner*	nmr
Points Count	16384	*Pulse Sequence*	zg	*Receiver Gain*	122.56	*SW(cyclical) (Hz)*	8012.82
Solvent	CHLOROFORM-d	*Spectrum Offset (Hz)*	2461.4089	*Spectrum Type*	STANDARD	*Sweep Width (Hz)*	8012.33
Temperature (degree C)	40.000						

MAN7524.003.esp

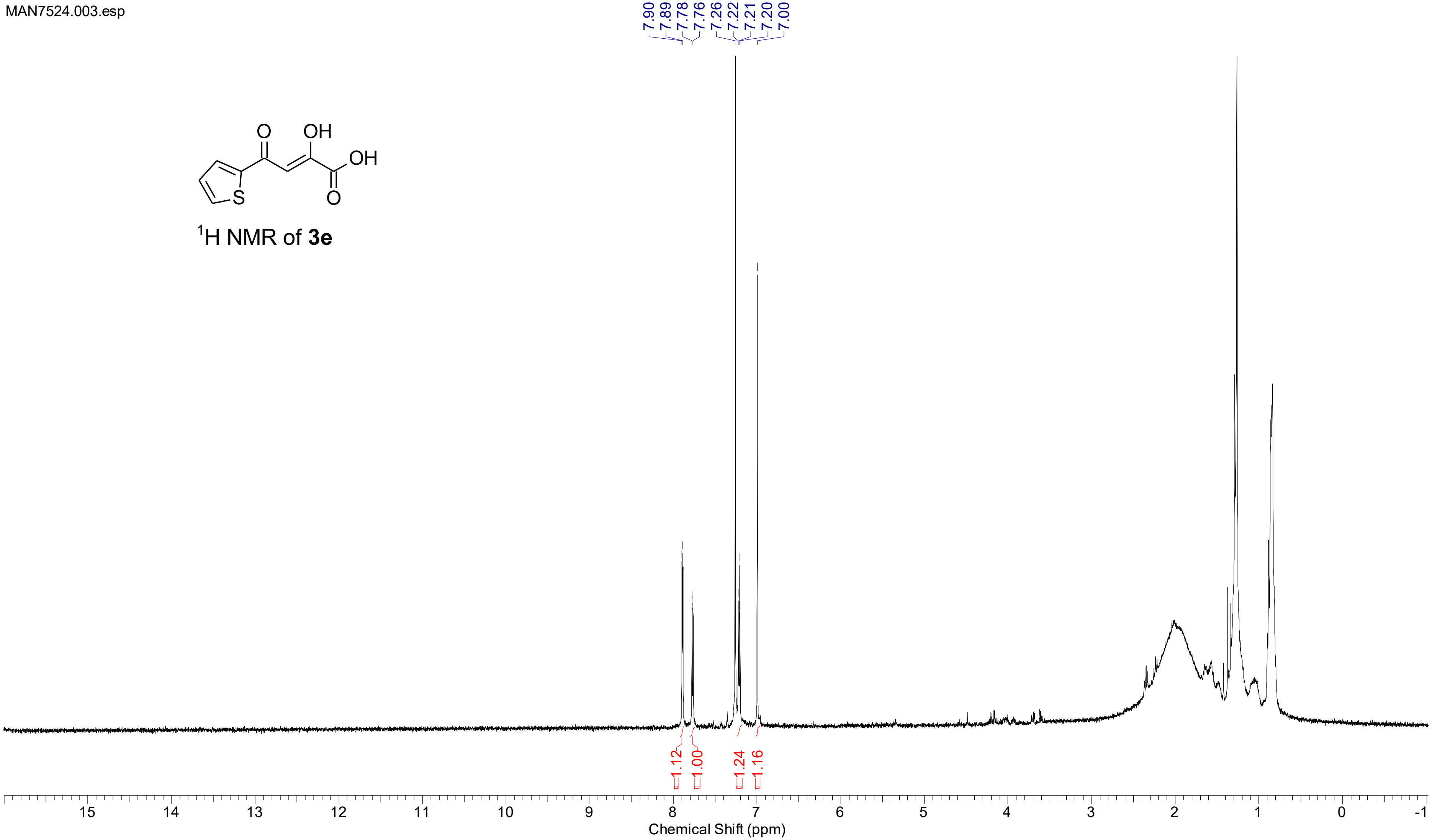

^{1}H NMR of **3e**

Acquisition Time (sec)	2.0447								
						Frequency (MHz)	400.17	***Nucleus***	1H
Number of Transients	8	***Origin***	spect	***Original Points Count***	16384	***Owner***	nmr	***Points Count***	16384
Pulse Sequence	zg	***Receiver Gain***	138.37	***SW(cyclical) (Hz)***	8012.82	***Solvent***	CHLOROFORM-d		
Spectrum Offset (Hz)	2461.4089	***Spectrum Type***	STANDARD	***Sweep Width (Hz)***	8012.33	***Temperature (degree C)***	39.998		

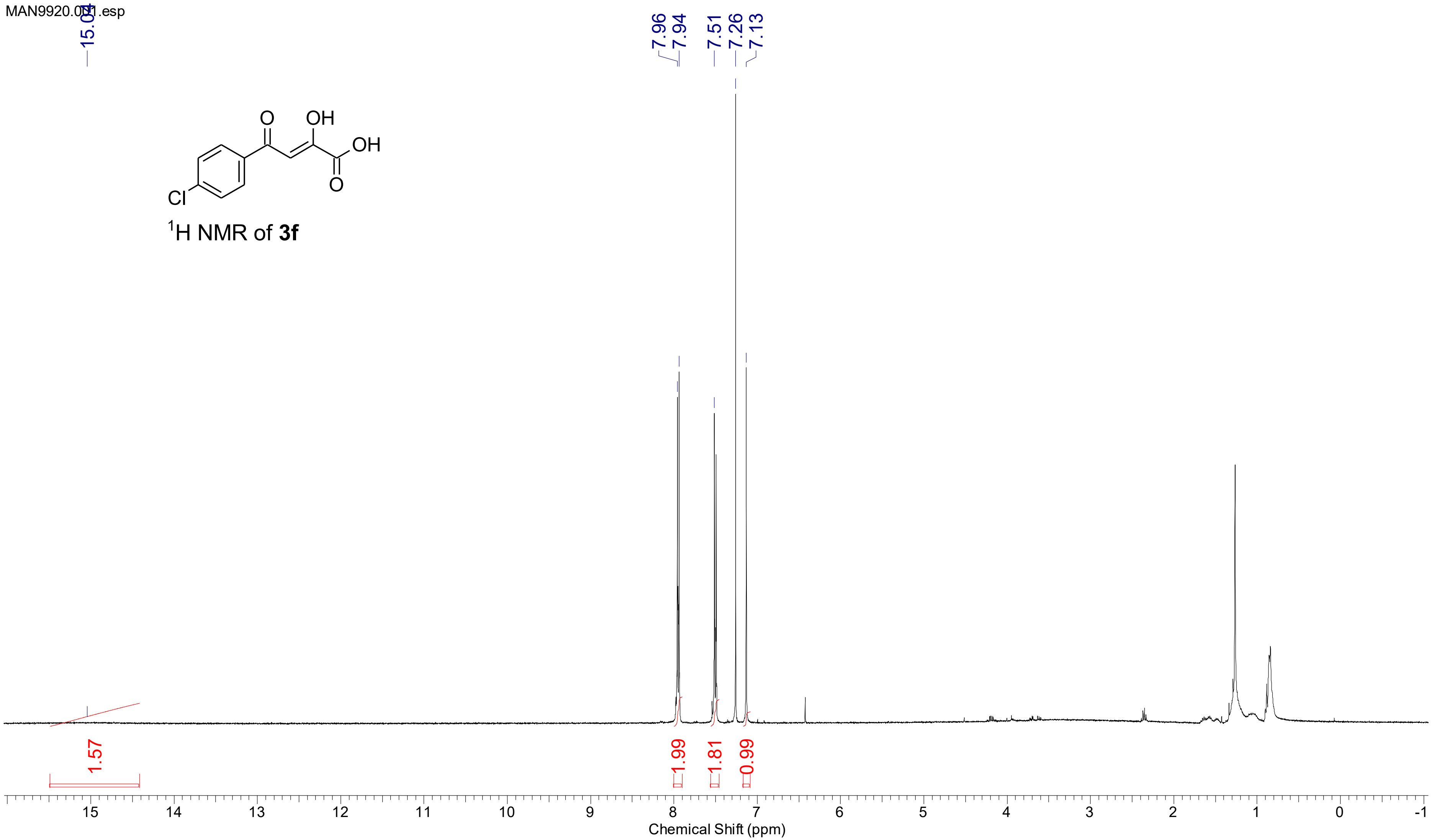

^{1}H NMR of **3f**

Acquisition Time (sec)	2.0447						
				Frequency (MHz)	400.17	*Nucleus*	1H
Number of Transients	8	*Origin*	spect	*Original Points Count*	16384	*Owner*	nmr
Points Count	16384						
Pulse Sequence	zg	*Receiver Gain*	122.56	*SW(cyclical) (Hz)*	8012.82	*Solvent*	CHLOROFORM-d
Spectrum Offset (Hz)	2461.4089	*Spectrum Type*	STANDARD	*Sweep Width (Hz)*	8012.33	*Temperature (degree C)*	39.999

MAN8371.001.esp

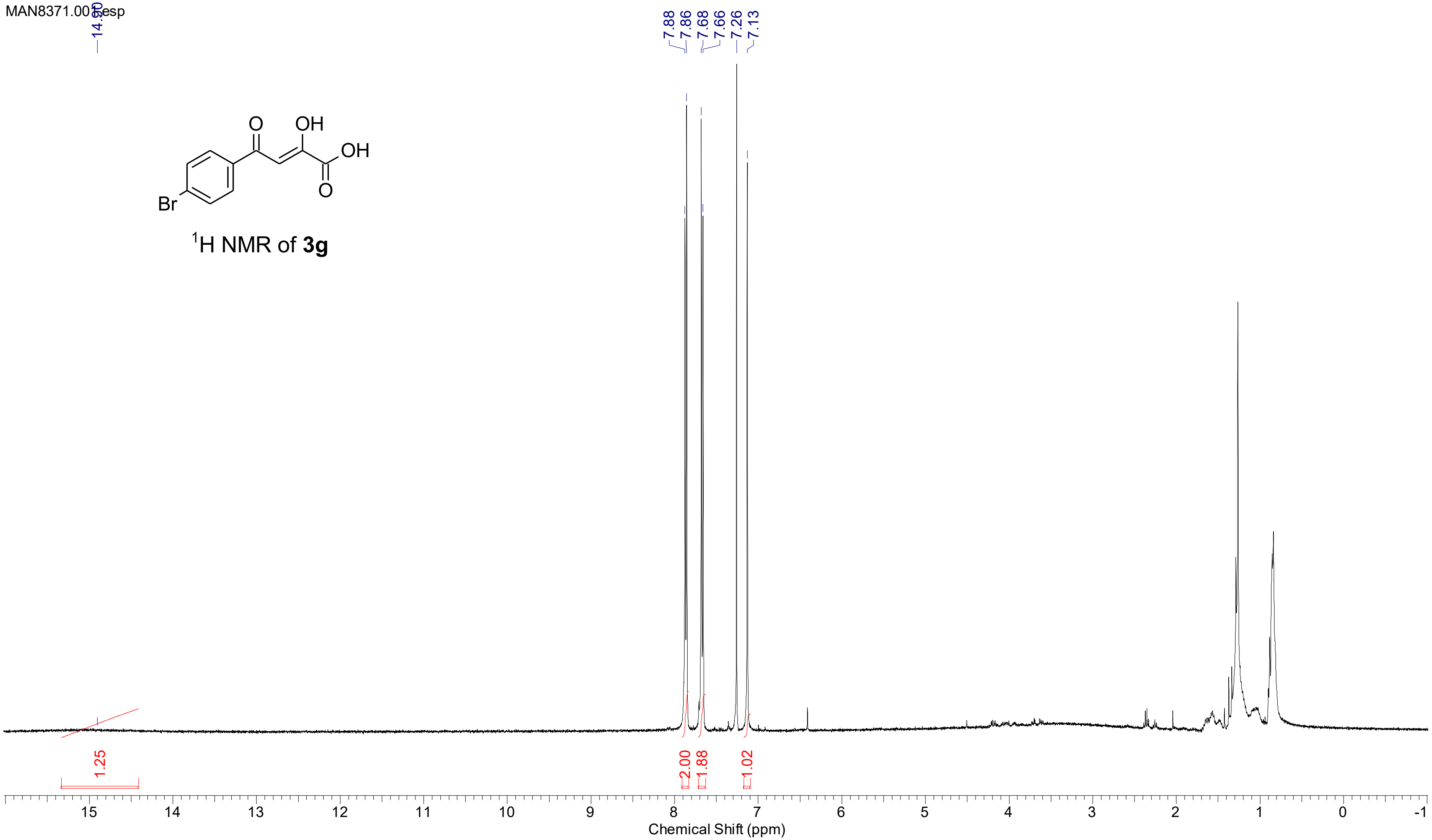

^{1}H NMR of **3g**

Acquisition Time (sec)	2.0447						
				Frequency (MHz)	400.17	***Nucleus***	1H
Number of Transients	8	***Origin***	spect	***Original Points Count***	16384	***Owner***	nmr
Points Count	16384	***Pulse Sequence***	zg	***Receiver Gain***	138.37	***SW(cyclical) (Hz)***	8012.82
Solvent	CHLOROFORM-d	***Spectrum Offset (Hz)***	2461.4089	***Spectrum Type***	STANDARD	***Sweep Width (Hz)***	8012.33
Temperature (degree C)	40.000						

MAN9921-001.esp

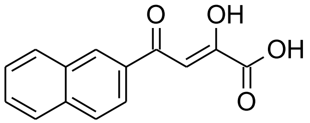

^{1}H NMR of **3h**

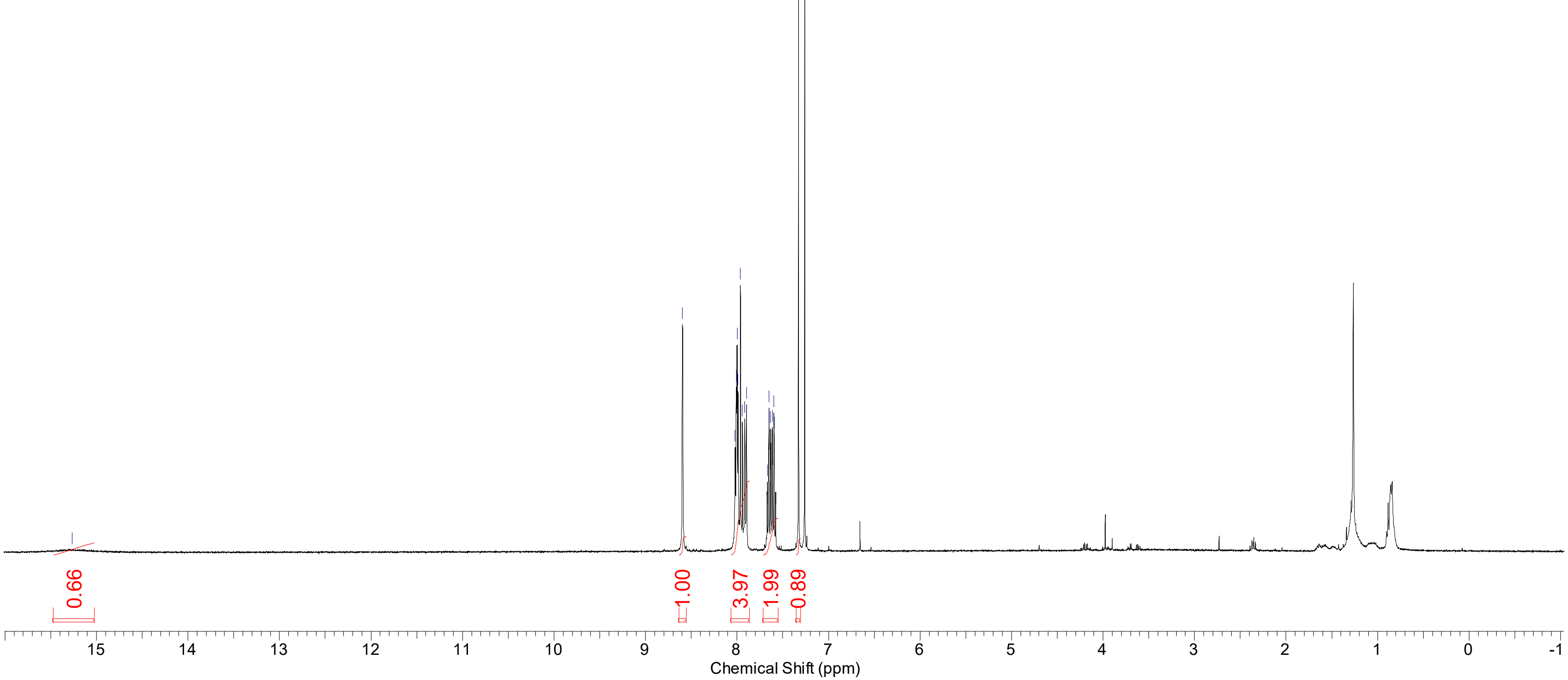

Acquisition Time (sec)	2.0447								
						Frequency (MHz)	400.17	Nucleus	1H
Number of Transients	8	Origin	spect	Original Points Count	16384	Owner	nmr	Points Count	16384
Pulse Sequence	zg	Receiver Gain	87.04	SW(cyclical) (Hz)	8012.82	Solvent	CHLOROFORM-d		
Spectrum Offset (Hz)	2461.4089	Spectrum Type	STANDARD	Sweep Width (Hz)	8012.33	Temperature (degree C)	39.999		

MAN9915.001.esp

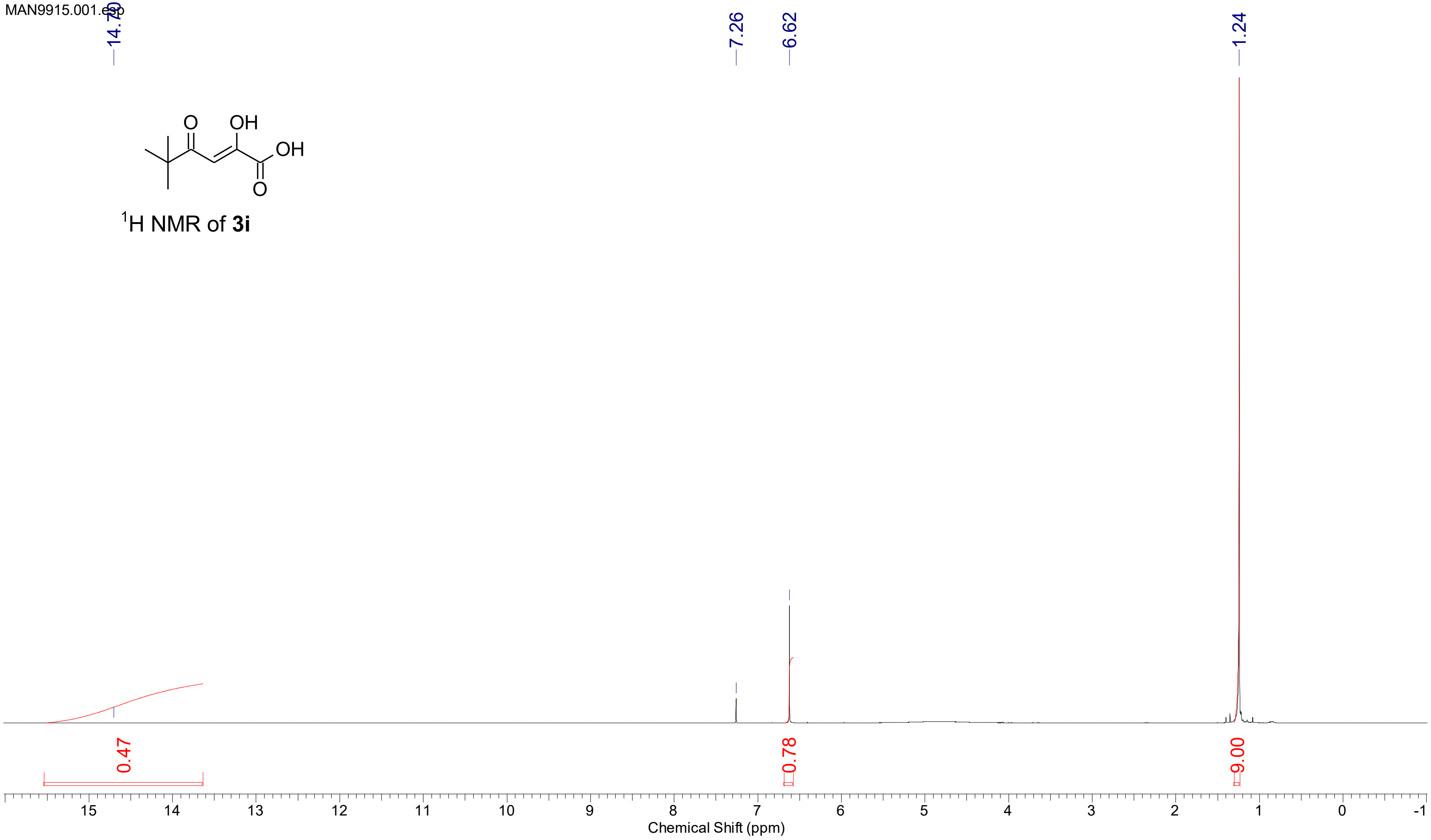

^{1}H NMR of **3i**

NMR chart of compound 4

Acquisition Time (sec)	2.0447						
				Frequency (MHz)	400.17	***Nucleus***	1H
Number of Transients	8	***Origin***	spect	***Original Points Count***	16384	***Owner***	nmr
Points Count	16384	***Pulse Sequence***	zg	***Receiver Gain***	109.22	***SW(cyclical) (Hz)***	8012.82
Solvent	DMSO-d6	***Spectrum Offset (Hz)***	2467.8608	***Spectrum Type***	STANDARD	***Sweep Width (Hz)***	8012.33
Temperature (degree C)	40.002						

MAN9914.001.esp

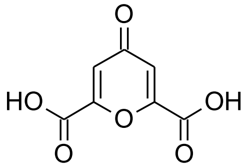

^{1}H NMR of **4**

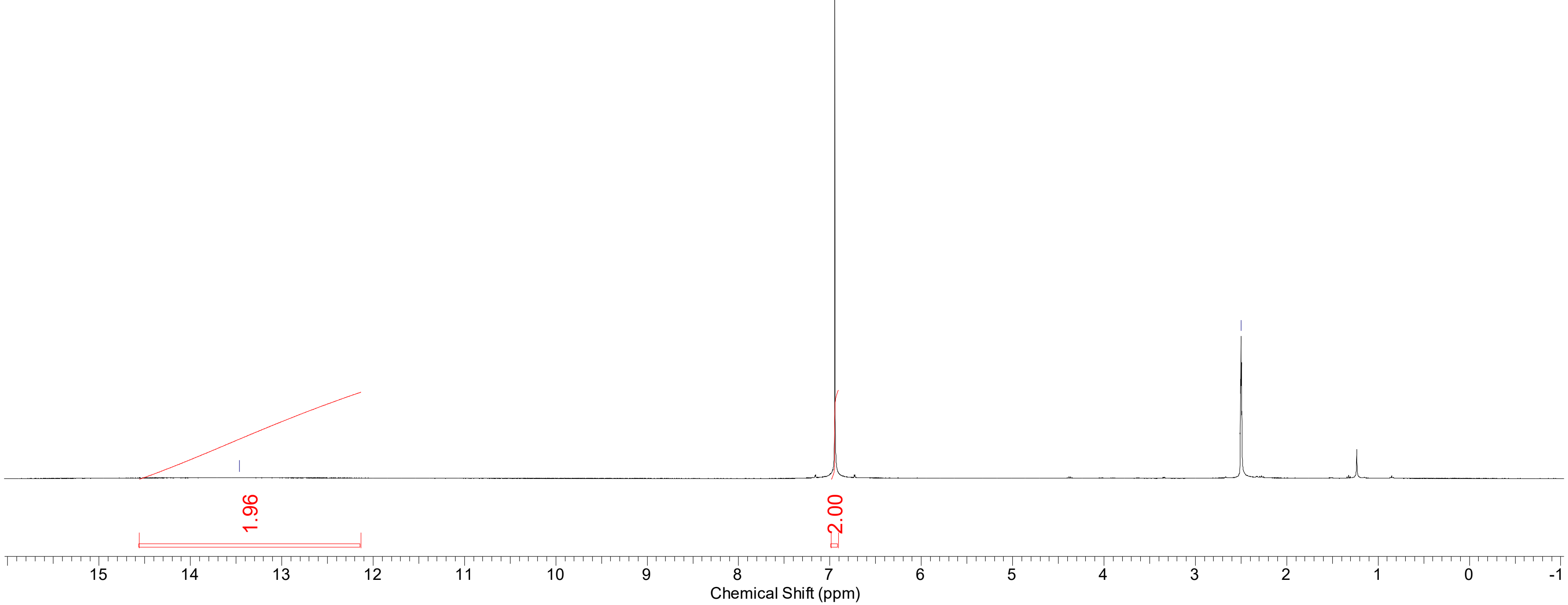

NMR charts of compounds 6a-g

Acquisition Time (sec)	4.5438										
						Frequency (MHz)	400.17	***Nucleus***	1H		
Number of Transients	32	***Origin***	spect	***Original Points Count***	32768	***Owner***	nmr	***Points Count***	32768		
Pulse Sequence	zg30	***Receiver Gain***	196.95	***SW(cyclical) (Hz)***	7211.54	***Solvent***	DMSO-d6	***Spectrum Offset (Hz)***	2797.9177		
Spectrum Type	STANDARD	***Sweep Width (Hz)***	7211.32	***Temperature (degree C)***	40.001						

MAN7892.001.esp

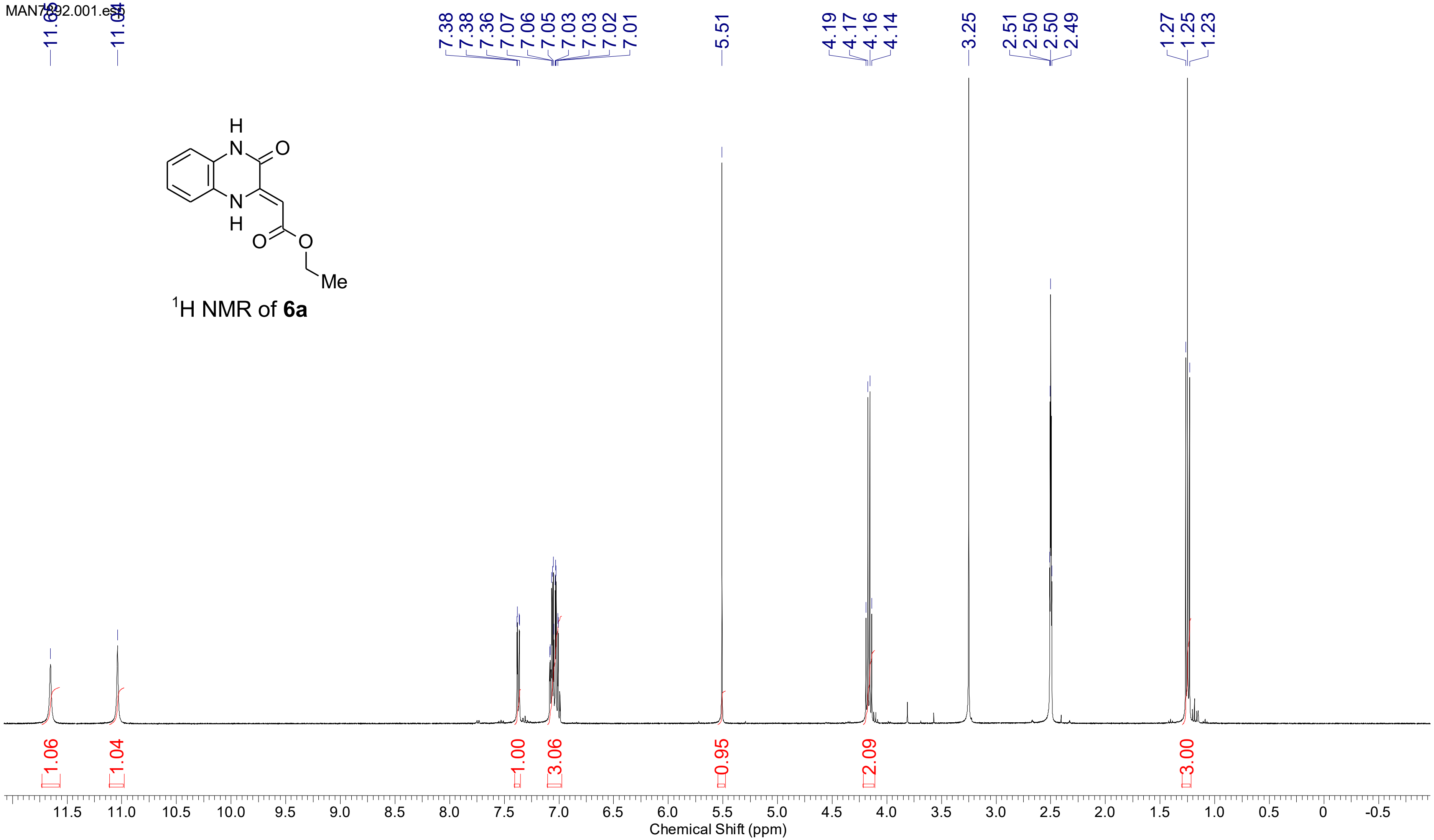

^{1}H NMR of **6a**

Acquisition Time (sec)	4.5438										
						Frequency (MHz)	400.17	***Nucleus***	1H		
Number of Transients	32	***Origin***	spect	***Original Points Count***	32768	***Owner***	nmr	***Points Count***	32768		
Pulse Sequence	zg30	***Receiver Gain***	196.95	***SW(cyclical) (Hz)***	7211.54	***Solvent***	DMSO-d6	***Spectrum Offset (Hz)***	2797.9177		
Spectrum Type	STANDARD	***Sweep Width (Hz)***	7211.32	***Temperature (degree C)***	40.001						

MAN7891.001.esp

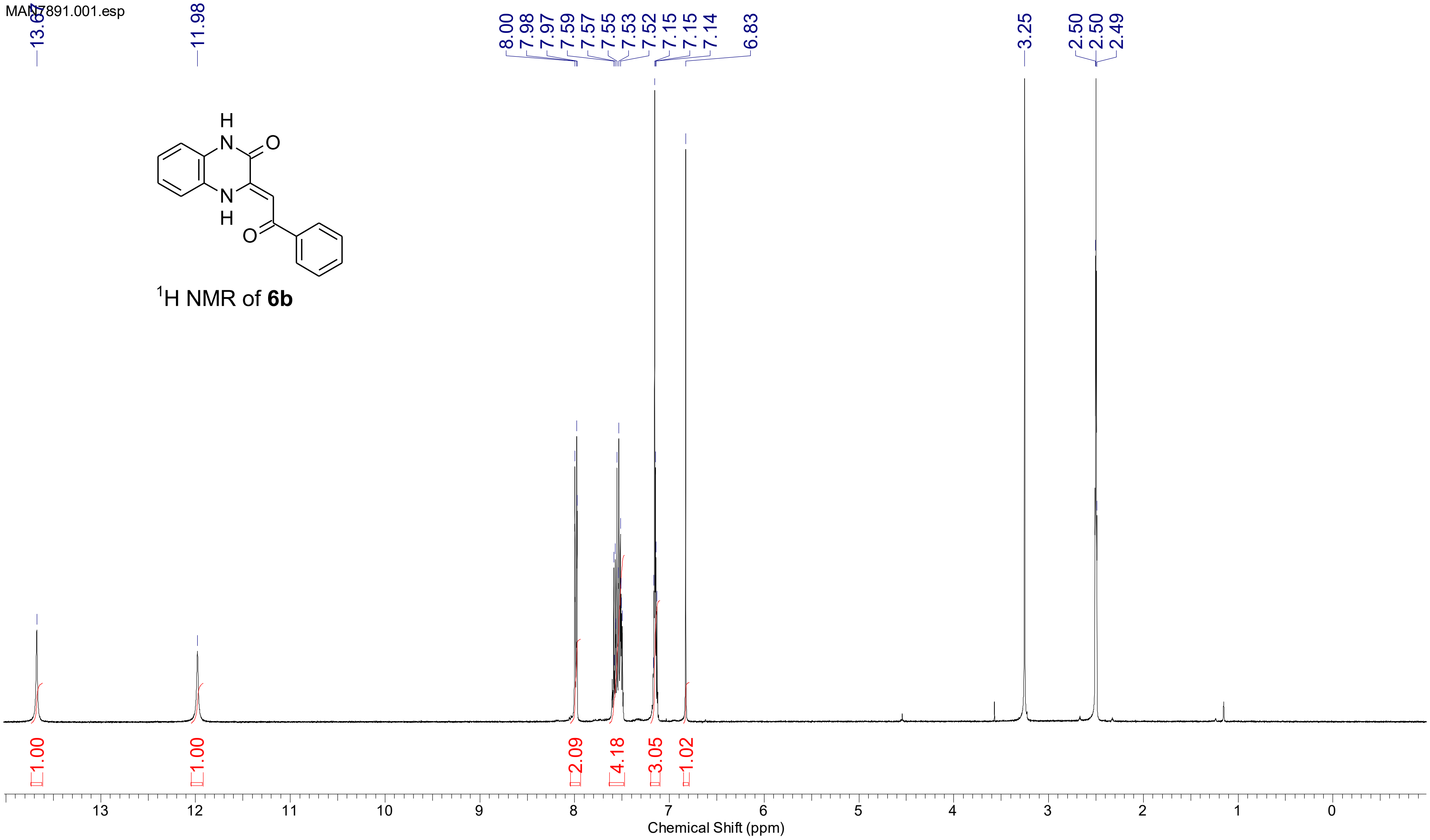

^{1}H NMR of **6b**

Acquisition Time (sec)	4.5438						
				Frequency (MHz)	400.17	***Nucleus***	1H
Number of Transients	32	***Origin***	spect	***Original Points Count***	32768	***Owner***	nmr	***Points Count***	32768
Pulse Sequence	zg30	***Receiver Gain***	196.95	***SW(cyclical) (Hz)***	7211.54	***Solvent***	DMSO-d6	***Spectrum Offset (Hz)***	2797.9177
Spectrum Type	STANDARD	***Sweep Width (Hz)***	7211.32	***Temperature (degree C)***	39.999				

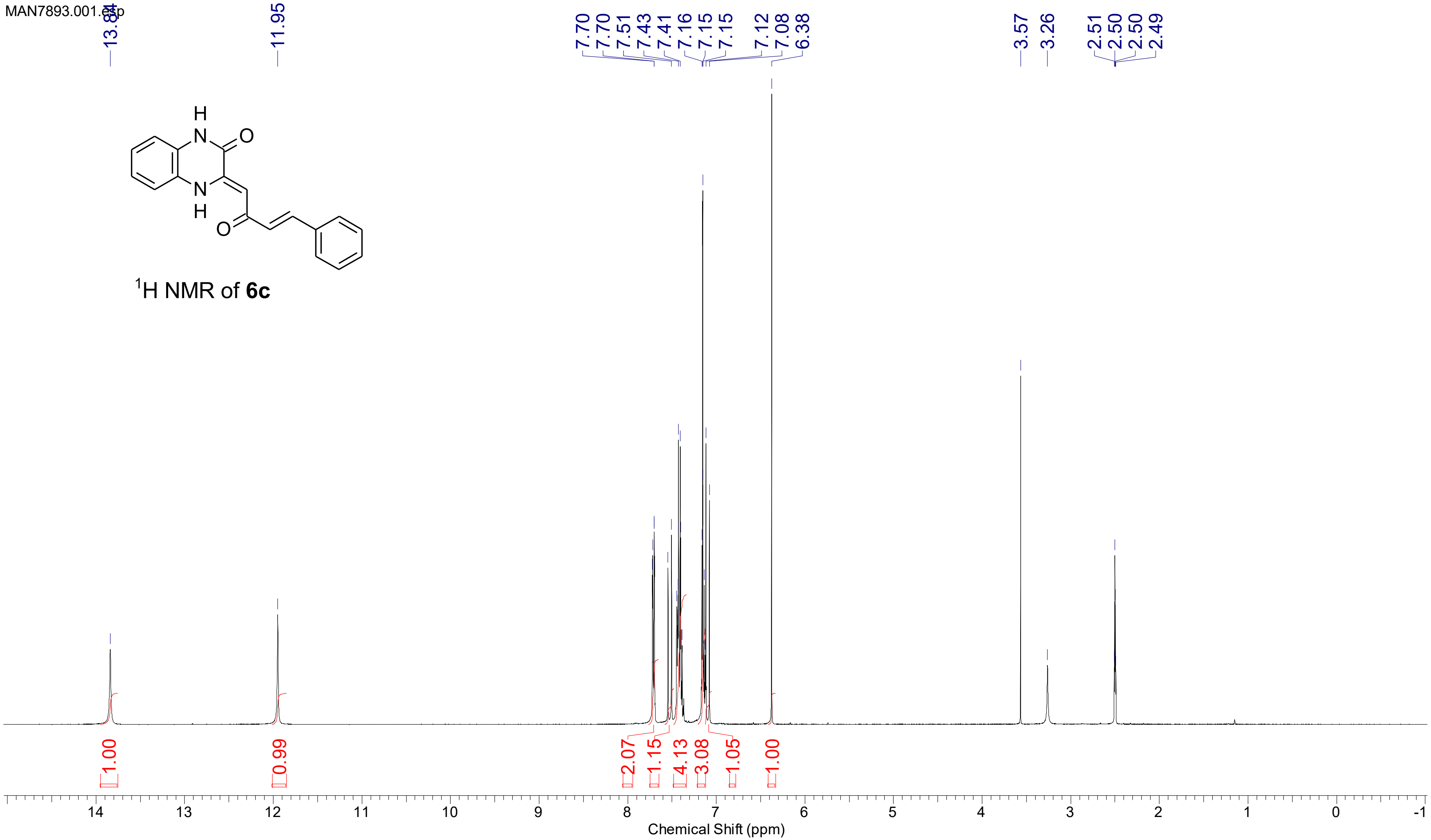

Acquisition Time (sec)	2.0447								
						Frequency (MHz)	400.17	***Nucleus***	1H
Number of Transients	8	***Origin***	spect	***Original Points Count***	16384	***Owner***	nmr	***Points Count***	16384
Pulse Sequence	zg	***Receiver Gain***	95.56	***SW(cyclical) (Hz)***	8012.82	***Solvent***	DMSO-d6	***Spectrum Offset (Hz)***	2467.8608
Spectrum Type	STANDARD	***Sweep Width (Hz)***	8012.33	***Temperature (degree C)***	40.000				

MAN9930.001.esp

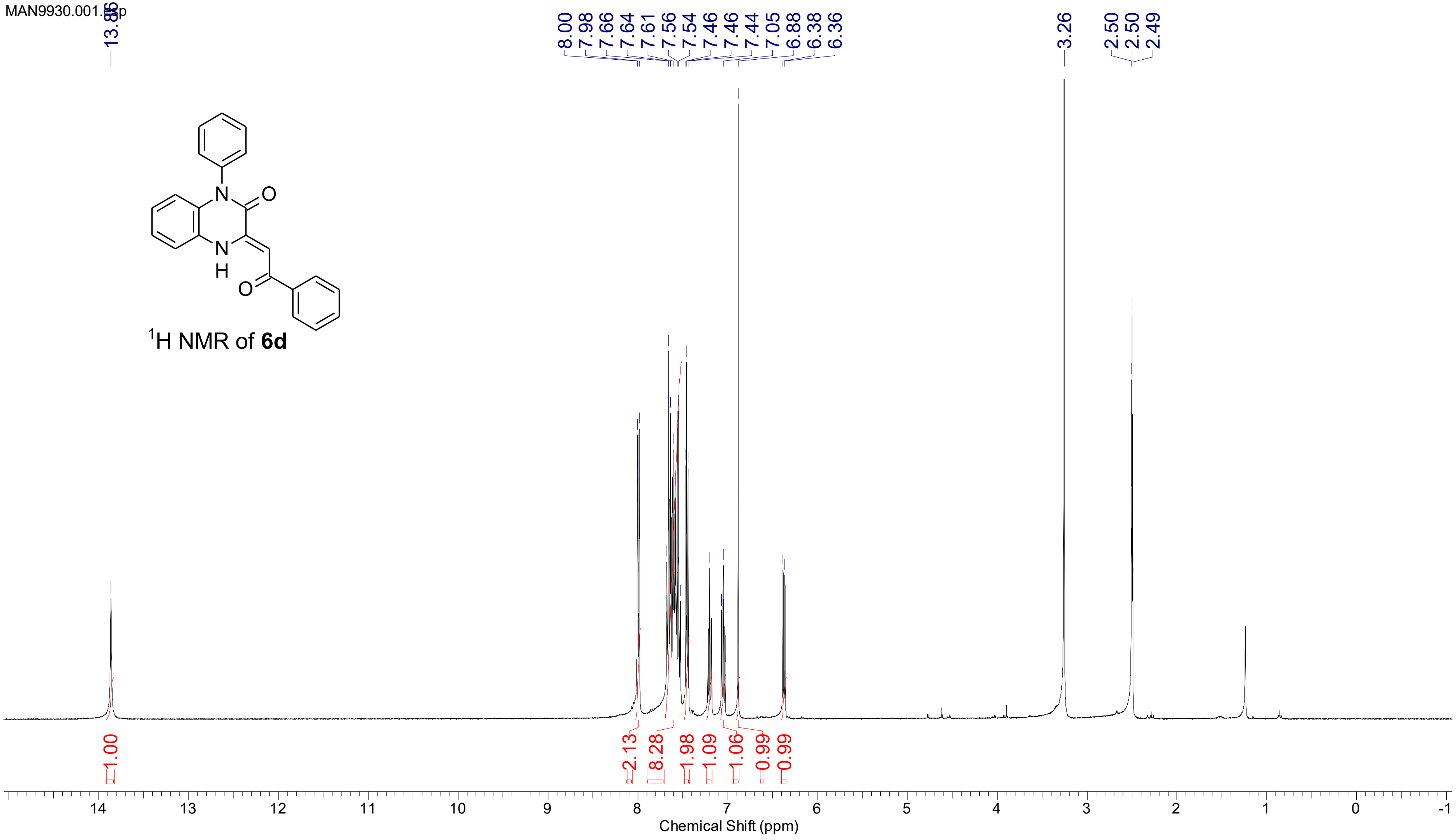

^{1}H NMR of **6d**

**Acquisition Time (sec)**	2.0447						
				**Frequency (MHz)**	400.17	**Nucleus**	1H
**Number of Transients**	8	**Origin**	spect	**Original Points Count**	16384	**Owner**	nmr
**Points Count**	16384						
**Pulse Sequence**	zg	**Receiver Gain**	87.04	**SW(cyclical) (Hz)**	8012.82	**Solvent**	DMSO-d6
**Spectrum Offset (Hz)**	2467.8608						
**Spectrum Type**	STANDARD	**Sweep Width (Hz)**	8012.33	**Temperature (degree C)**	39.996		

MAN9929.001.esp

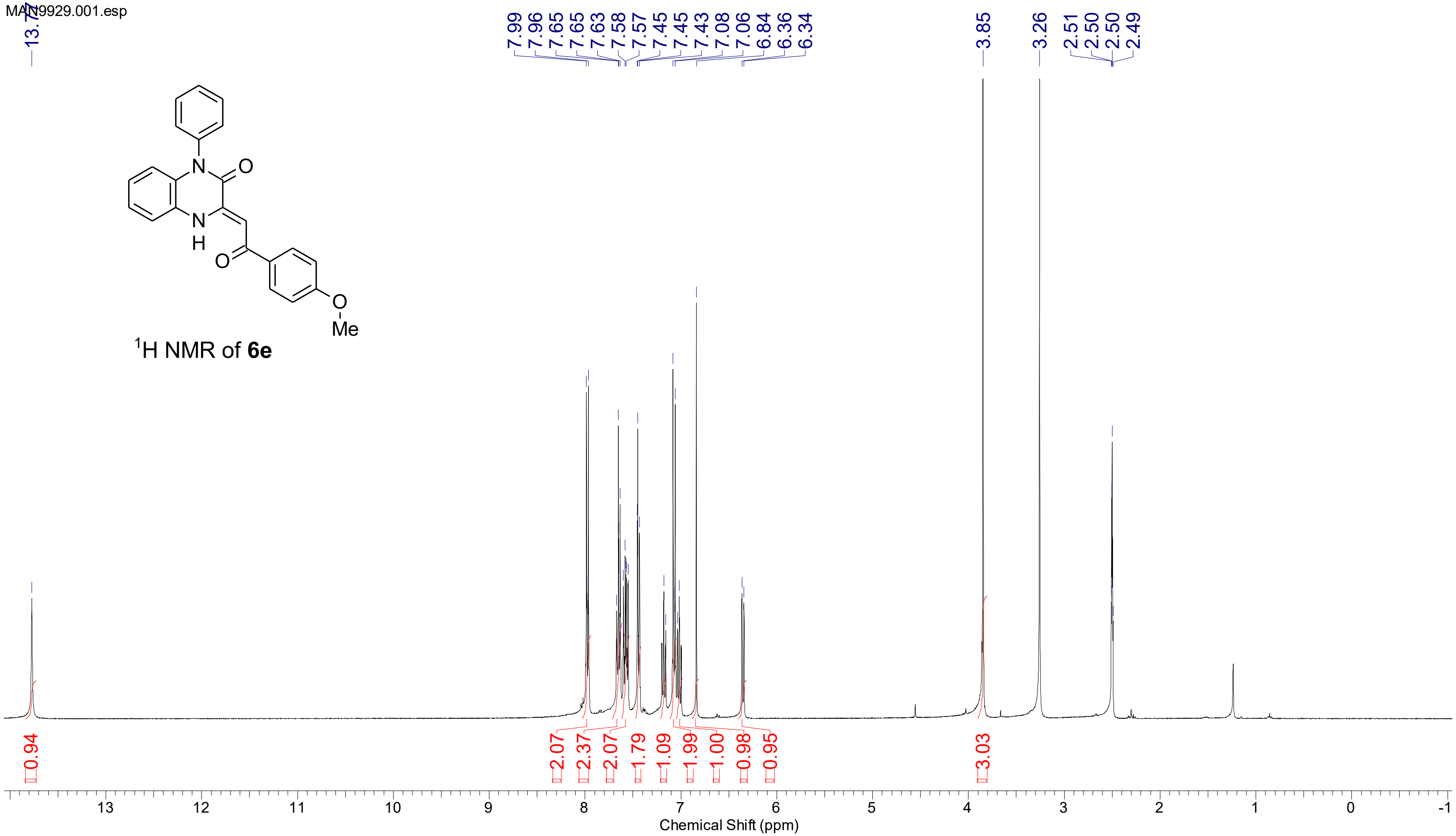

^{1}H NMR of **6e**

Acquisition Time (sec)	2.9999								
Frequency (MHz)	400.17	***Nucleus***	1H	***Number of Transients***	8	***Origin***	spect	***Original Points Count***	24038
Owner	nmr	***Points Count***	32768	***Pulse Sequence***	zg	***Receiver Gain***	47.43	***SW(cyclical) (Hz)***	8012.82
Solvent	DMSO-d6	***Spectrum Offset (Hz)***	2467.9829	***Spectrum Type***	STANDARD	***Sweep Width (Hz)***	8012.58	***Temperature (degree C)***	39.999

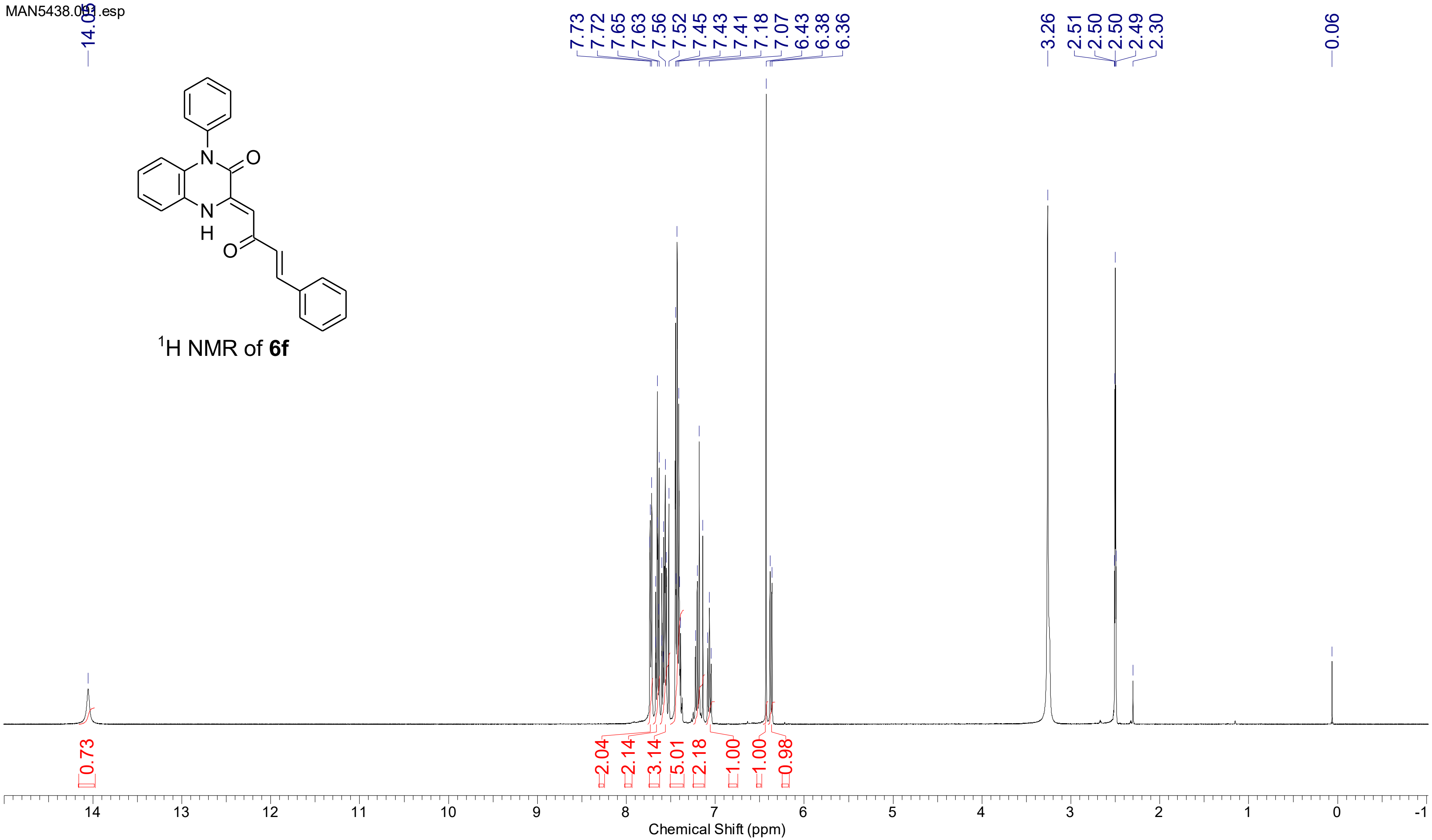

^{1}H NMR of **6f**

Acquisition Time (sec)	1.2976								
Frequency (MHz)	100.62	***Nucleus***	13C	***Number of Transients***	512	***Origin***	spect	***Original Points Count***	32768
Owner	nmr	***Points Count***	1048576	***Pulse Sequence***	zgpg30	***Receiver Gain***	196.95	***SW(cyclical) (Hz)***	25252.53
Solvent	DMSO-d6	***Spectrum Offset (Hz)***	11511.0947	***Spectrum Type***	STANDARD	***Sweep Width (Hz)***	25252.50	***Temperature (degree C)***	40.000

MAN5438.002.esp

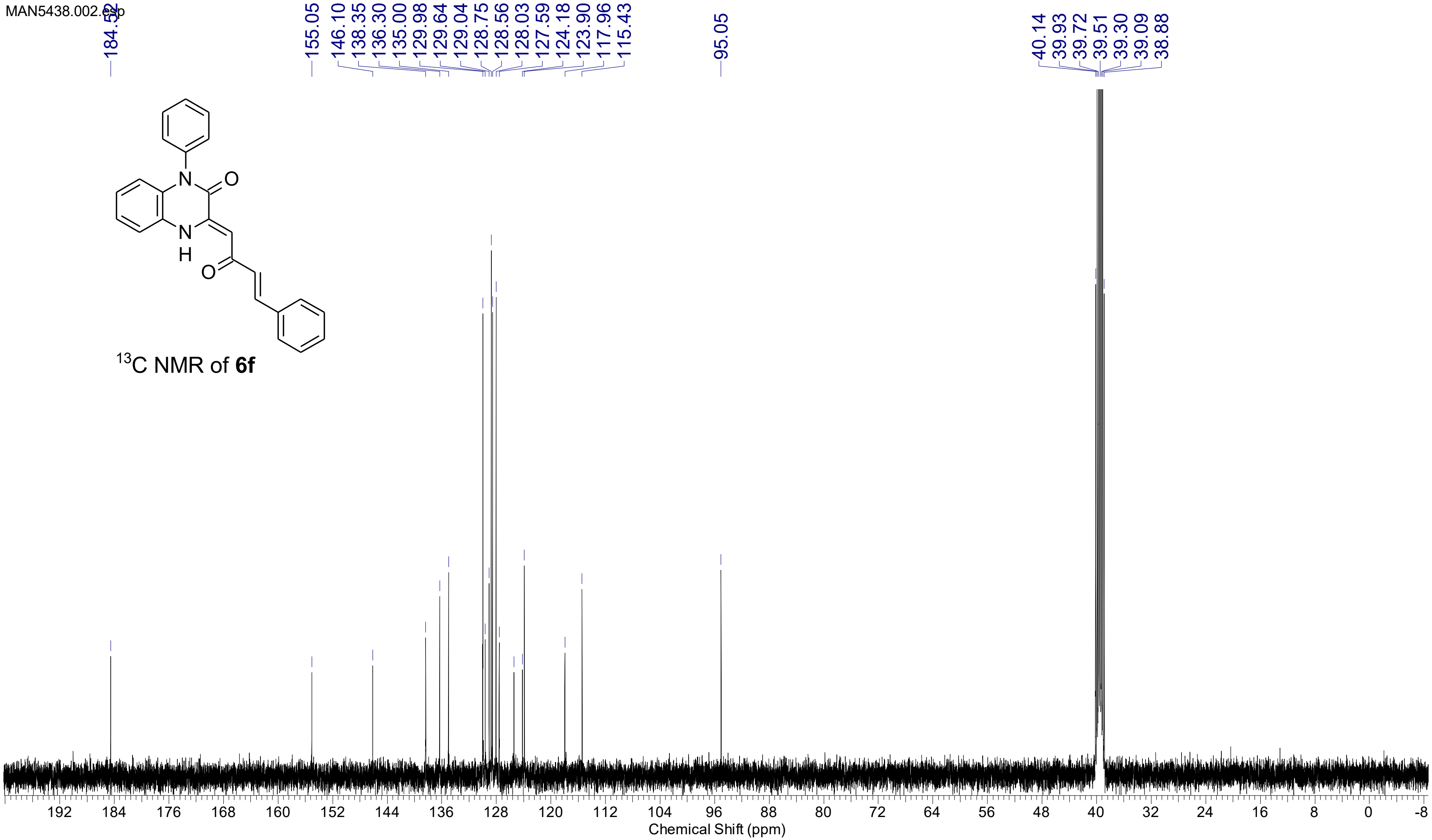

^{13}C NMR of **6f**

Acquisition Time (sec)	2.9999								
Frequency (MHz)	400.17	***Nucleus***	1H	***Number of Transients***	8	***Origin***	spect	***Original Points Count***	24038
Owner	nmr	***Points Count***	32768	***Pulse Sequence***	zg	***Receiver Gain***	95.56	***SW(cyclical) (Hz)***	8012.82
Solvent	DMSO-d6	***Spectrum Offset (Hz)***	2467.9829	***Spectrum Type***	STANDARD	***Sweep Width (Hz)***	8012.58	***Temperature (degree C)***	39.998

MAN5252.001.esp

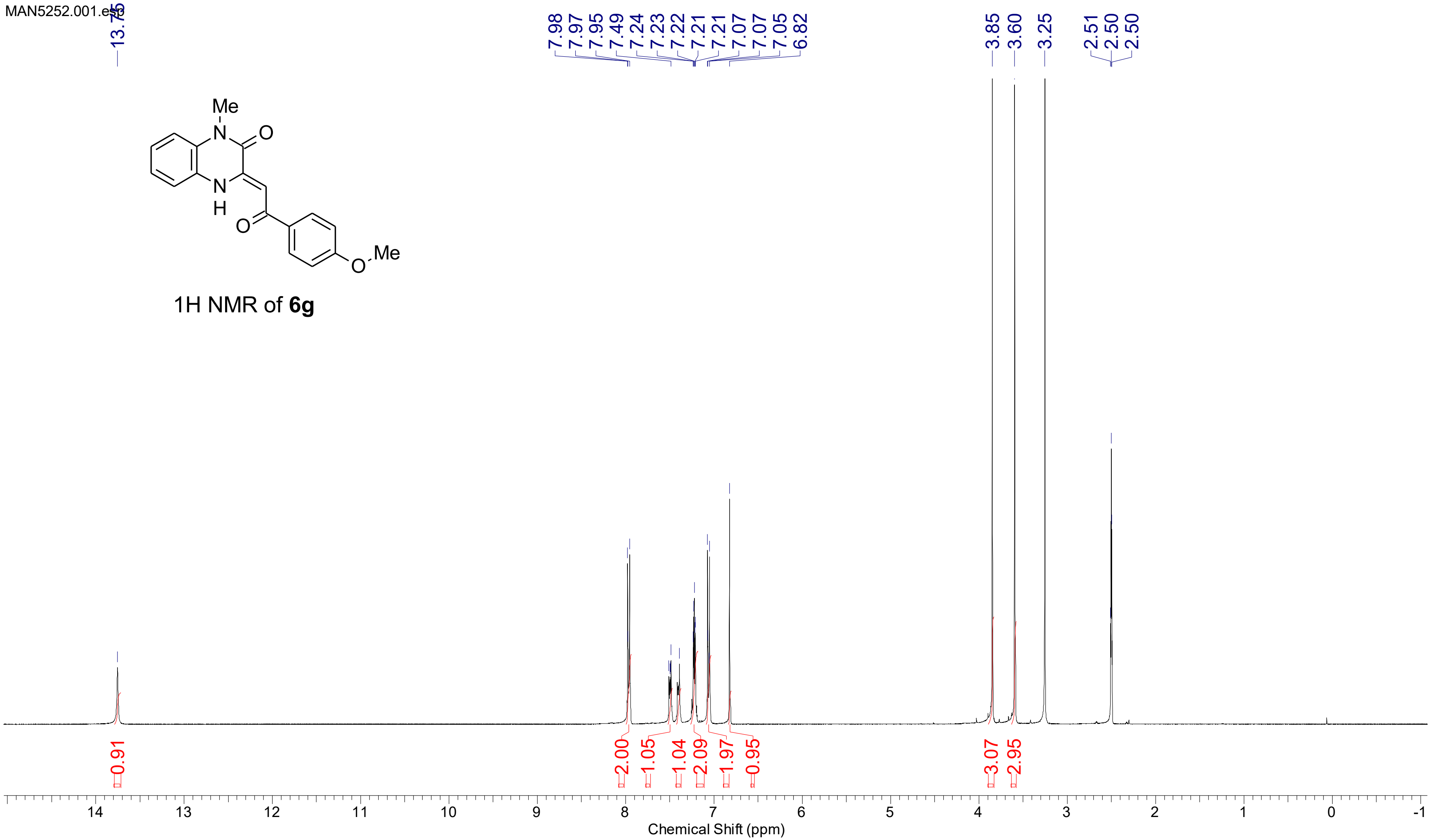

1H NMR of **6g**

NMR charts of compounds 7a-d

Acquisition Time (sec)	8.1789								
						Frequency (MHz)	400.17	***Nucleus***	1H
Number of Transients	1	***Origin***	spect	***Original Points Count***	65536	***Owner***	nmr	***Points Count***	65536
Pulse Sequence	zg	***Receiver Gain***	95.56	***SW(cyclical) (Hz)***	8012.82	***Solvent***	DMSO-d6	***Spectrum Offset (Hz)***	2467.4329
Spectrum Type	STANDARD	***Sweep Width (Hz)***	8012.70	***Temperature (degree C)***	39.956				

MAN6635_004.esp

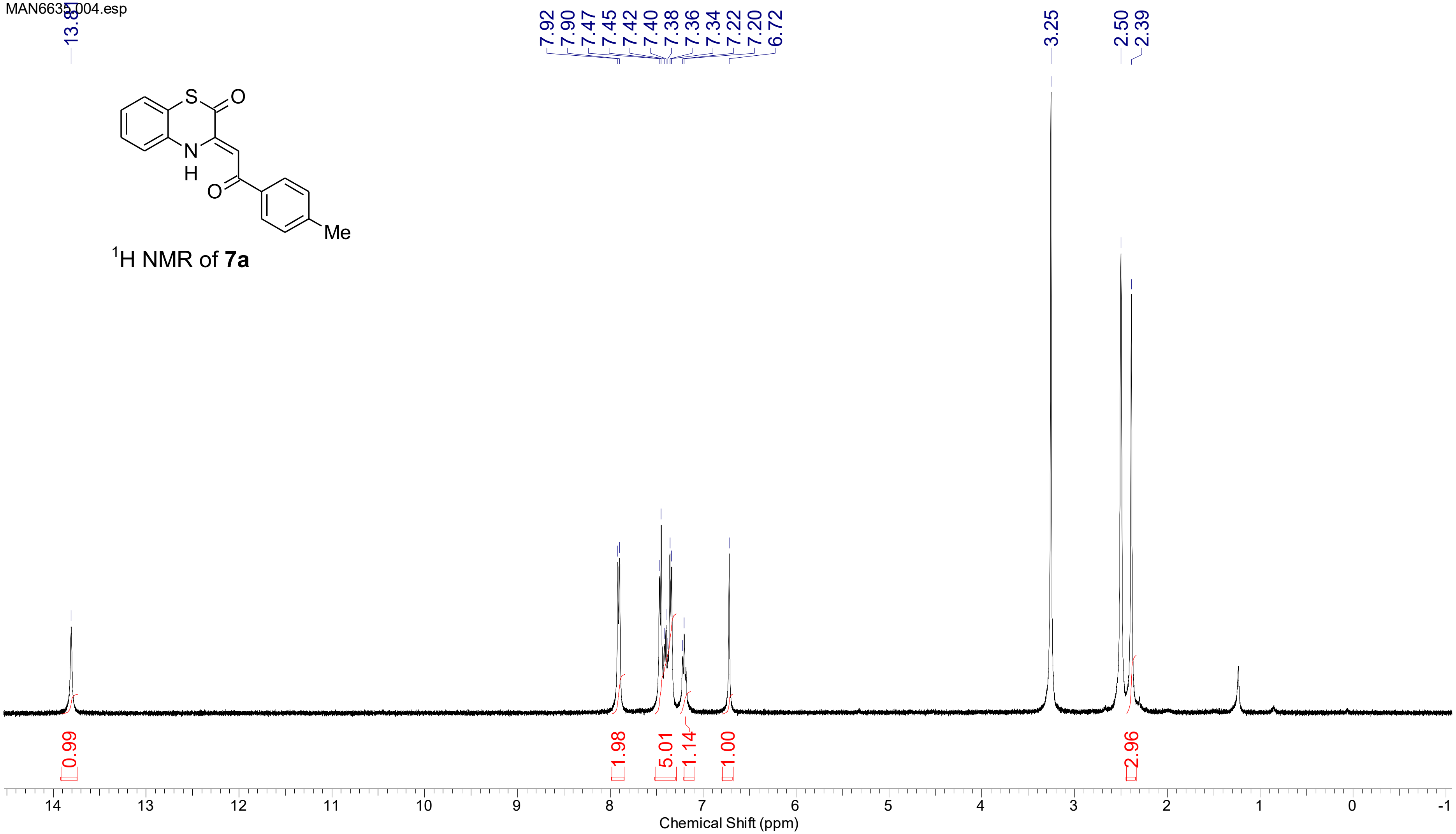

Acquisition Time (sec)	2.0447						
				Frequency (MHz)	400.17	Nucleus	1H
Number of Transients	8	Origin	spect	Original Points Count	16384	Owner	nmr	Points Count	16384
Pulse Sequence	zg	Receiver Gain	77.64	SW(cyclical) (Hz)	8012.82	Solvent	DMSO-d6	Spectrum Offset (Hz)	2467.8608
Spectrum Type	STANDARD	Sweep Width (Hz)	8012.33	Temperature (degree C)	39.994				

MAN769?001.esp

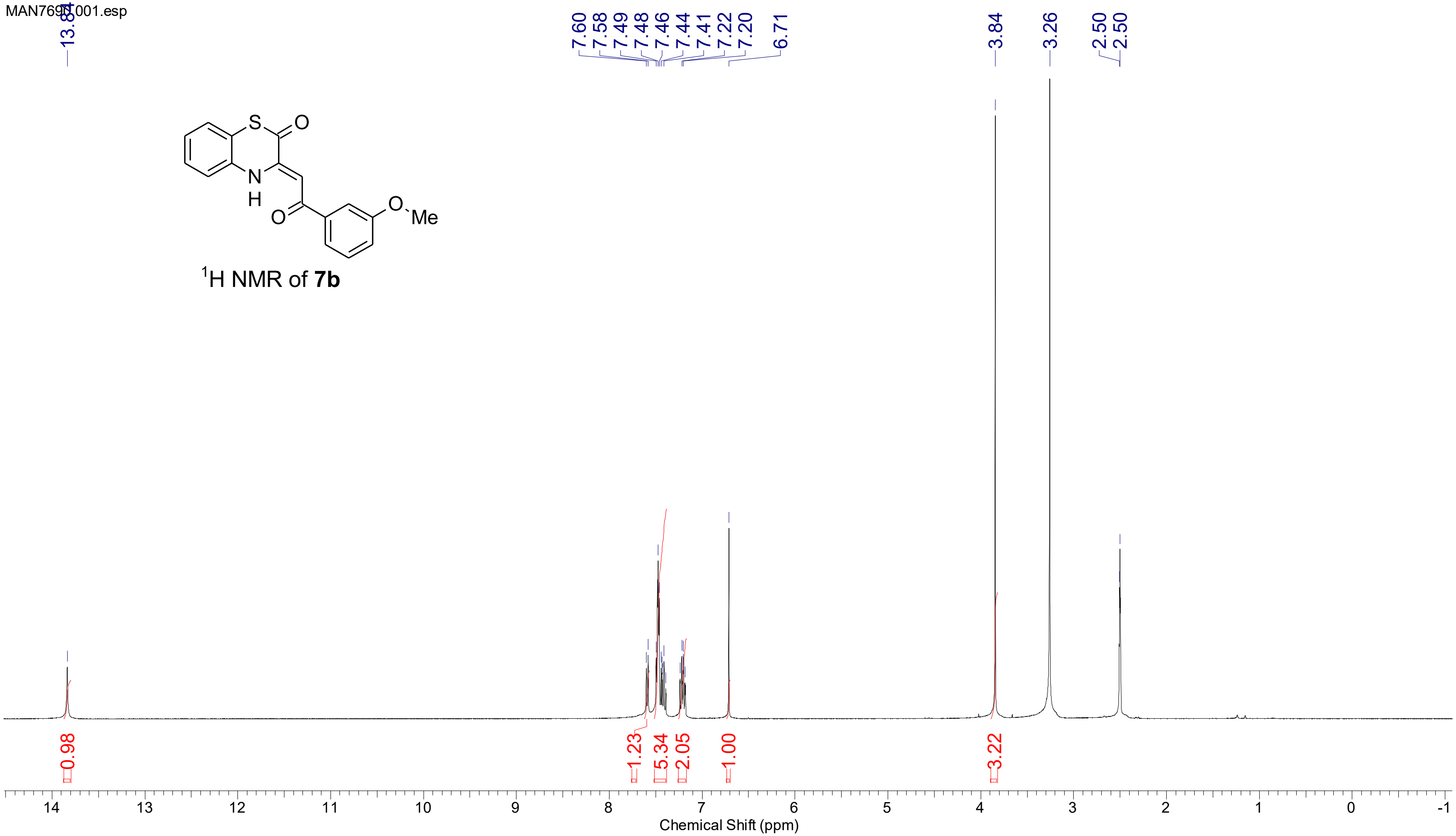

^{1}H NMR of **7b**

Acquisition Time (sec)	1.2976								
Frequency (MHz)	100.62	***Nucleus***	13C	***Number of Transients***	512	***Origin***	spect	***Original Points Count***	32768
Owner	nmr	***Points Count***	1048576	***Pulse Sequence***	zgpg30	***Receiver Gain***	196.95	***SW(cyclical) (Hz)***	25252.53
Solvent	DMSO-d6	***Spectrum Offset (Hz)***	11511.3809	***Spectrum Type***	STANDARD	***Sweep Width (Hz)***	25252.50	***Temperature (degree C)***	39.998

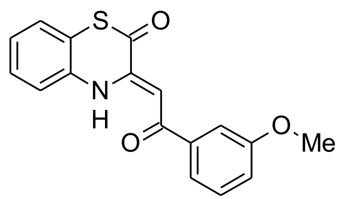

^{13}C NMR of **7b**

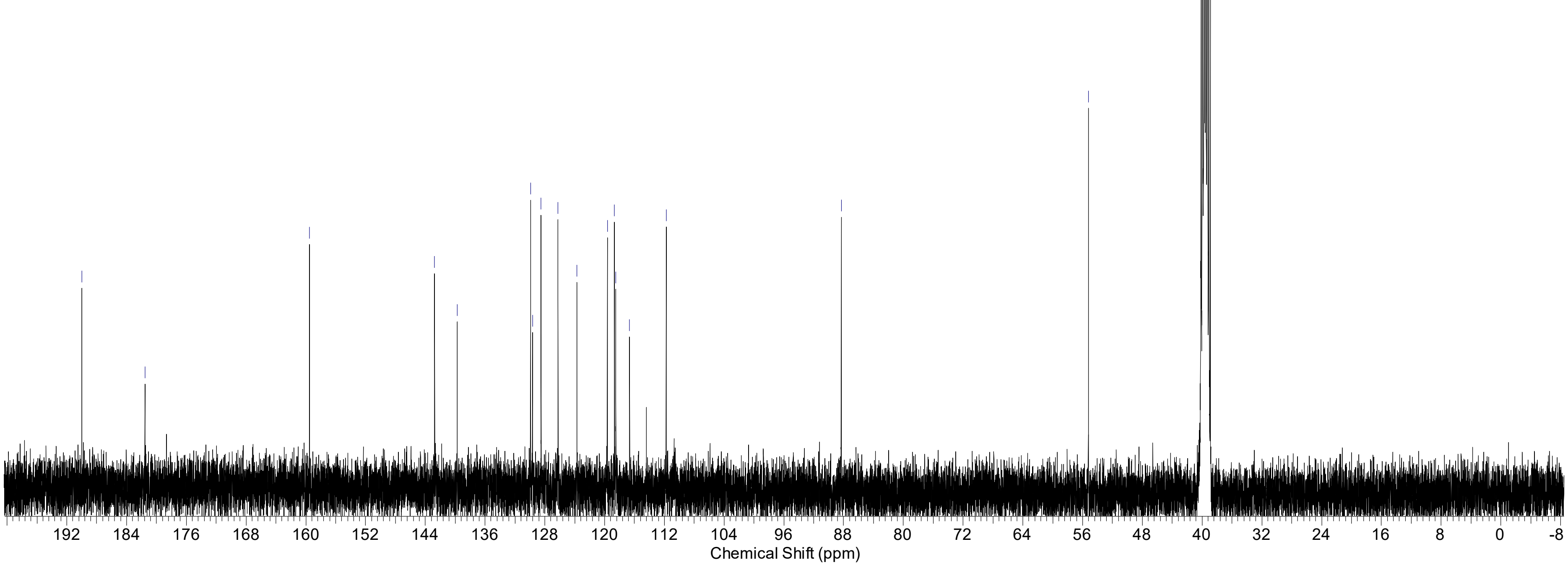

Acquisition Time (sec)	8.1789								
						Frequency (MHz)	400.17	Nucleus	1H
Number of Transients	8	Origin	spect	Original Points Count	65536	Owner	nmr	Points Count	65536
Pulse Sequence	zg	Receiver Gain	109.22	SW(cyclical) (Hz)	8012.82	Solvent	DMSO-d6	Spectrum Offset (Hz)	2467.6775
Spectrum Type	STANDARD	Sweep Width (Hz)	8012.70	Temperature (degree C)	40.006				

MAN6652010.esp

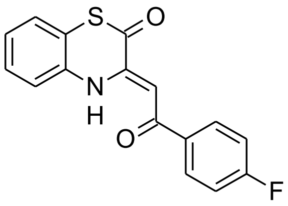

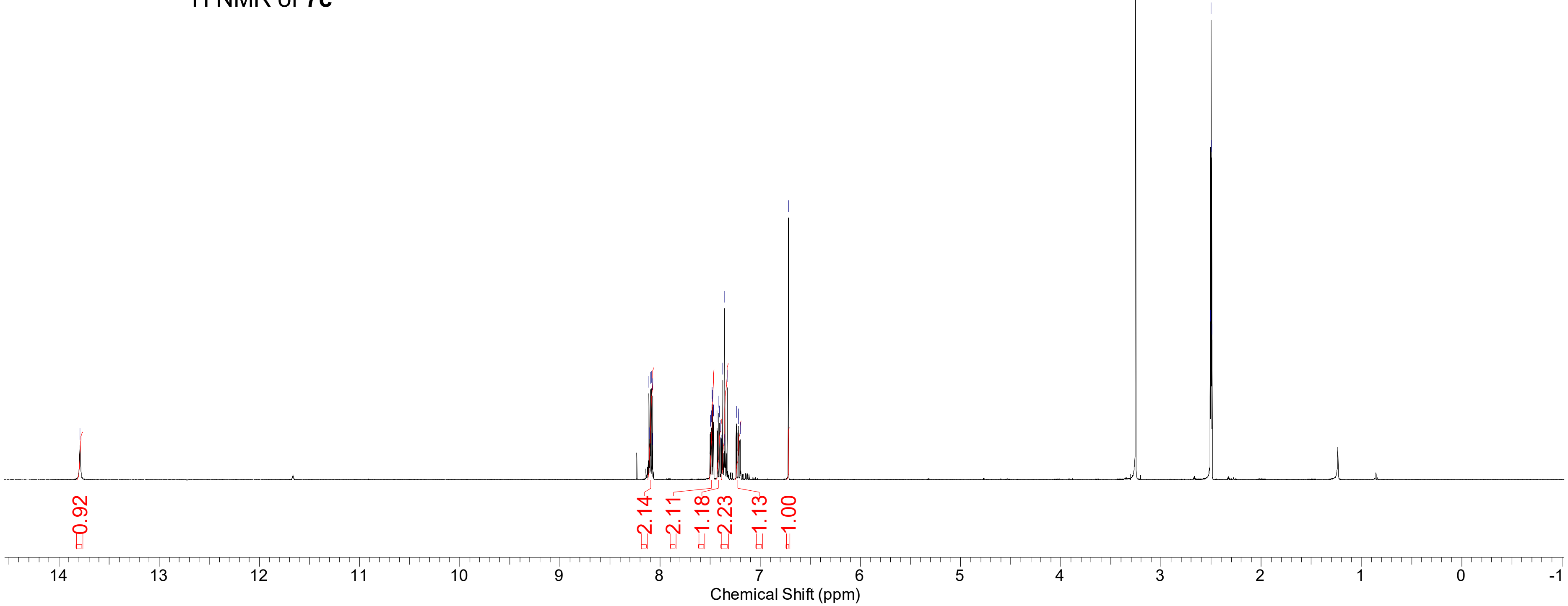

^{1}H NMR of **7c**

Acquisition Time (sec)	2.9999								
Frequency (MHz)	400.17	Nucleus	1H	Number of Transients	8	Origin	spect	Original Points Count	24038
Owner	nmr	Points Count	32768	Pulse Sequence	zg	Receiver Gain	109.22	SW(cyclical) (Hz)	8012.82
Solvent	DMSO-d6	Spectrum Offset (Hz)	2467.7385	Spectrum Type	STANDARD	Sweep Width (Hz)	8012.58	Temperature (degree C)	39.990

MAN62[illegible].001.esp

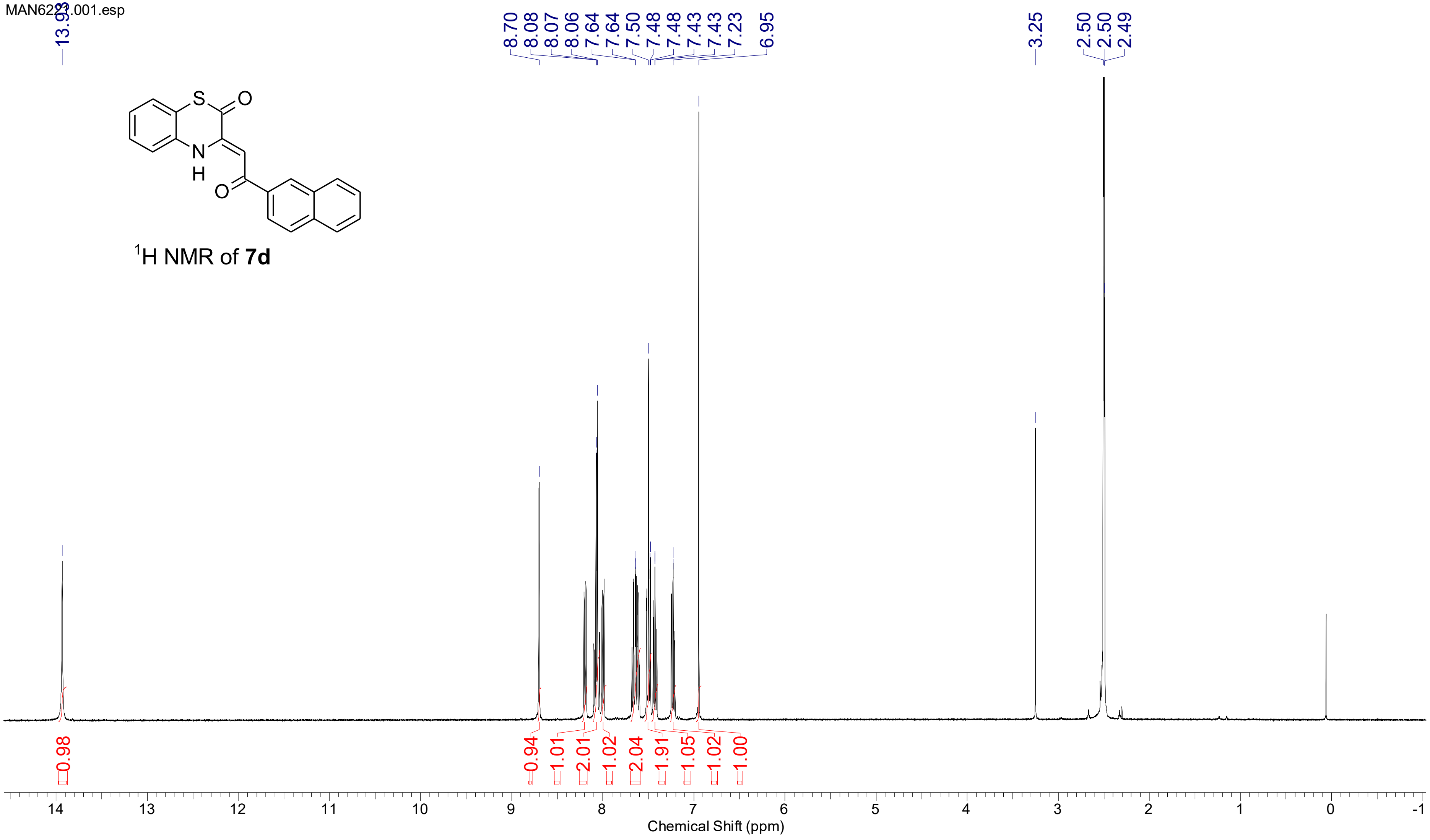

^{1}H NMR of **7d**

NMR chart of compound 8

Acquisition Time (sec)	4.5438								
Frequency (MHz)	400.17	***Nucleus***	1H	***Number of Transients***	64	***Origin***	spect	***Original Points Count***	32768
Owner	nmr	***Points Count***	32768	***Pulse Sequence***	zg30	***Receiver Gain***	196.95	***SW(cyclical) (Hz)***	7211.54
Solvent	CHLOROFORM-d			***Spectrum Offset (Hz)***	2791.7837	***Spectrum Type***	STANDARD	***Sweep Width (Hz)***	7211.32
Temperature (degree C)	40.001								

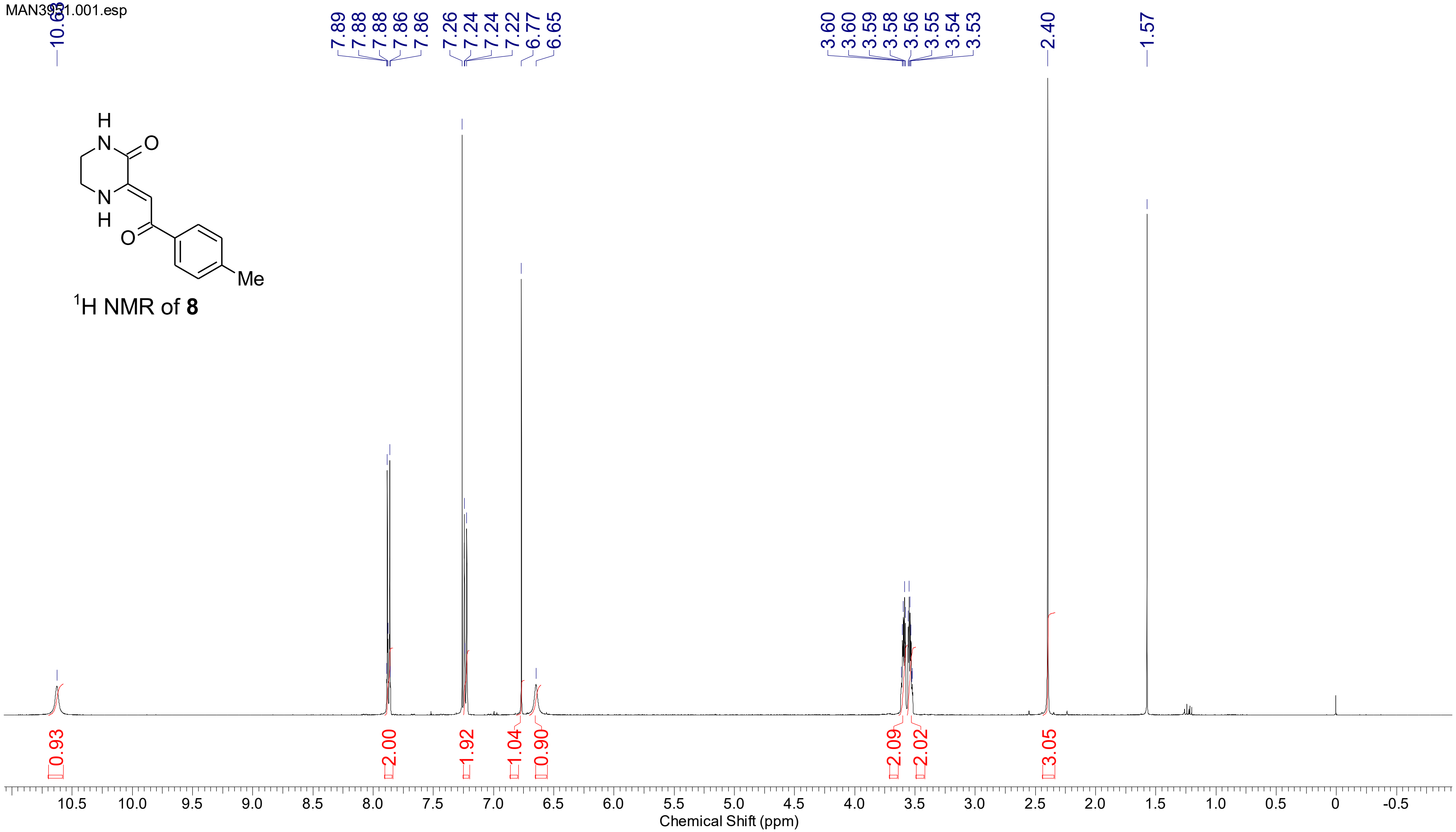

NMR charts of compounds 10a-q

Acquisition Time (sec)	2.9999								
Frequency (MHz)	400.17	*Nucleus*	1H	*Number of Transients*	8	*Origin*	spect	*Original Points Count*	24038
Owner	nmr	*Points Count*	32768	*Pulse Sequence*	zg	*Receiver Gain*	70.85	*SW(cyclical) (Hz)*	8012.82
Solvent	CHLOROFORM-d			*Spectrum Offset (Hz)*	2461.7759	*Spectrum Type*	STANDARD	*Sweep Width (Hz)*	8012.58
Temperature (degree C)	40.002								

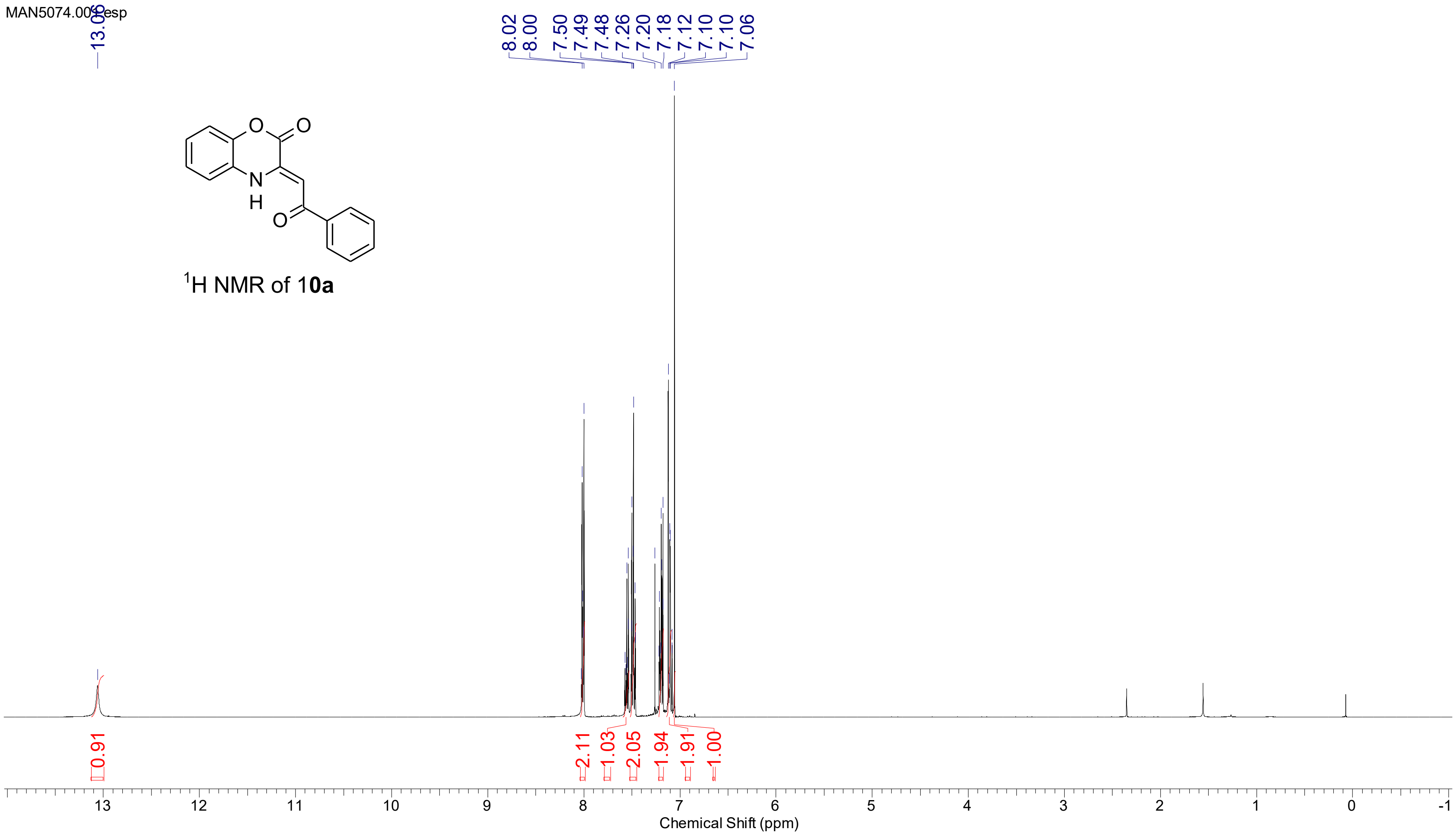

Acquisition Time (sec)	2.0447								
Frequency (MHz)	400.17	***Nucleus***	1H	***Number of Transients***	8	***Origin***	spect	***Original Points Count***	16384
Owner	nmr	***Points Count***	16384	***Pulse Sequence***	zg	***Receiver Gain***	43.76	***SW(cyclical) (Hz)***	8012.82
Solvent	DMSO-d6	***Spectrum Offset (Hz)***	2468.3499	***Spectrum Type***	STANDARD	***Sweep Width (Hz)***	8012.33	***Temperature (degree C)***	39.989

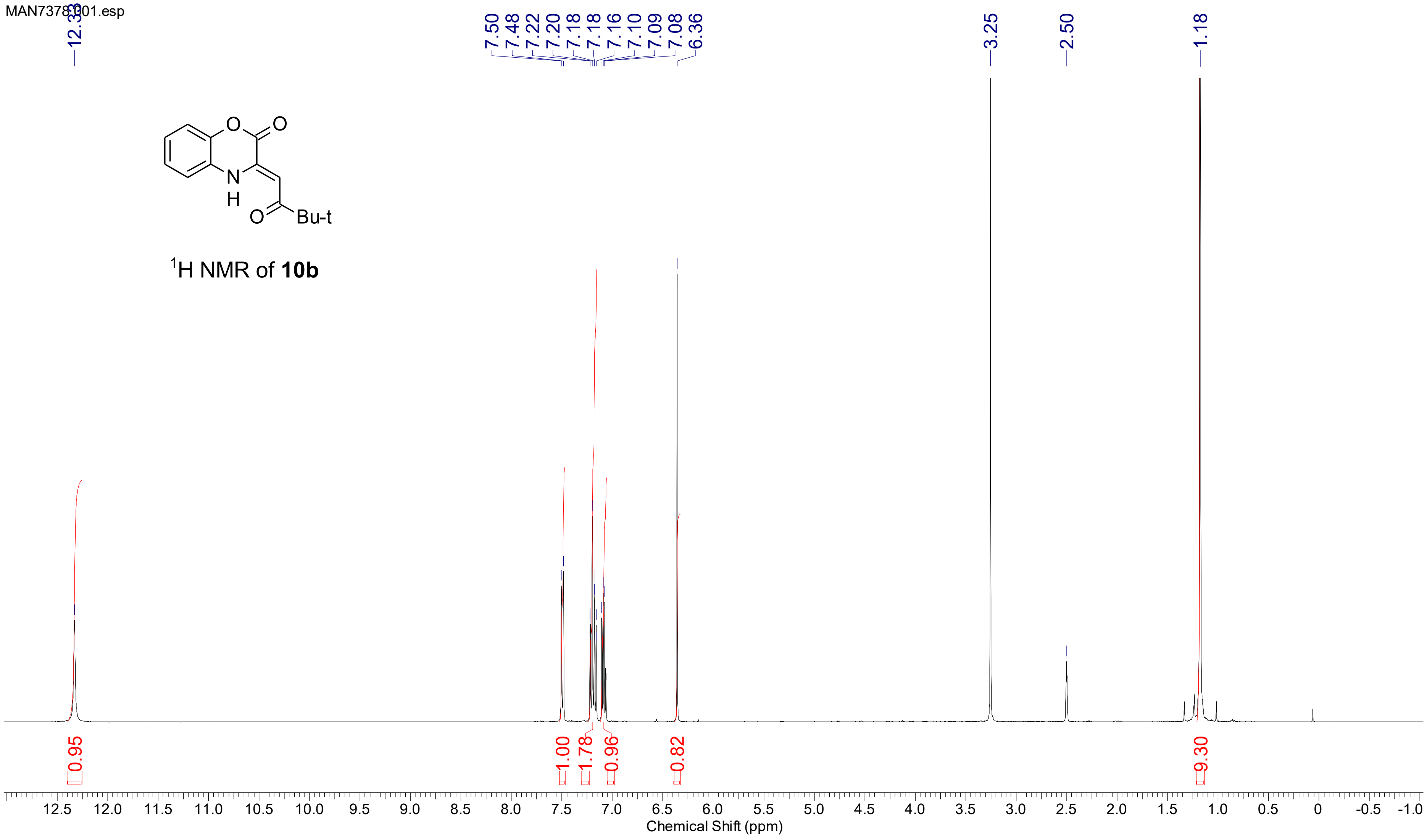

^{1}H NMR of **10b**

Acquisition Time (sec)	2.9999								
Frequency (MHz)	400.17	Nucleus	1H	Number of Transients	8	Origin	spect	Original Points Count	24038
Owner	nmr	Points Count	32768	Pulse Sequence	zg	Receiver Gain	47.43	SW(cyclical) (Hz)	8012.82
Solvent	DMSO-d6	Spectrum Offset (Hz)	2467.7385	Spectrum Type	STANDARD	Sweep Width (Hz)	8012.58	Temperature (degree C)	39.998

MAN5986.011.esp

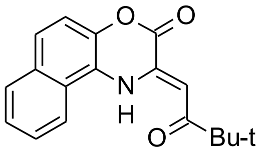

^{1}H NMR of **10c**

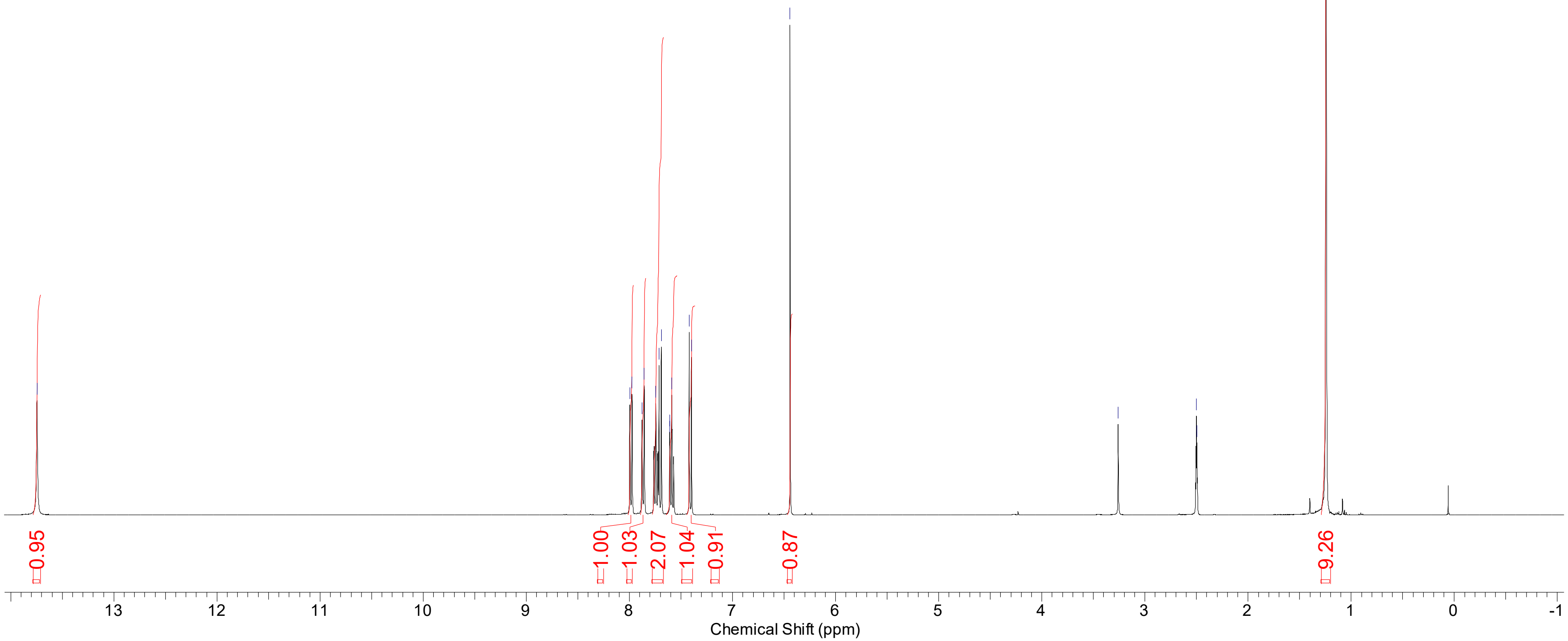

Acquisition Time (sec)	2.9999								
**Frequency (MHz)**	400.17	**Nucleus**	1H	**Number of Transients**	16	**Origin**	spect	**Original Points Count**	24038
**Owner**	nmr	**Points Count**	32768	**Pulse Sequence**	zg	**Receiver Gain**	70.85	**SW(cyclical) (Hz)**	8012.82
**Solvent**	DMSO-d6	**Spectrum Offset (Hz)**	2467.9829	**Spectrum Type**	STANDARD	**Sweep Width (Hz)**	8012.58	**Temperature (degree C)**	39.998

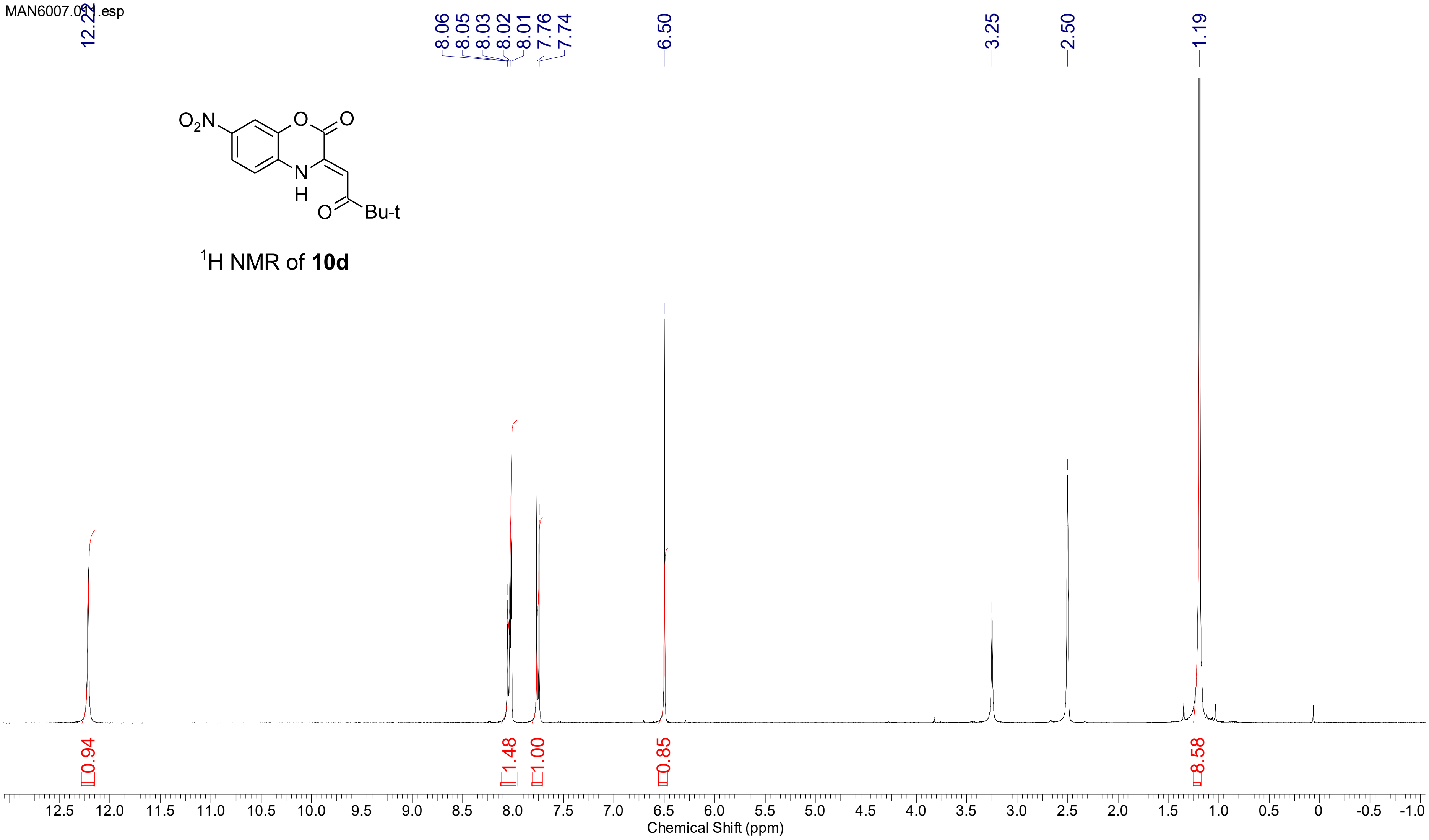

Acquisition Time (sec)	2.9999								
Frequency (MHz)	400.17	***Nucleus***	1H	***Number of Transients***	8	***Origin***	spect	***Original Points Count***	24038
Owner	nmr	***Points Count***	32768	***Pulse Sequence***	zg	***Receiver Gain***	77.64	***SW(cyclical) (Hz)***	8012.82
Solvent	DMSO-d6	***Spectrum Offset (Hz)***	2467.7385	***Spectrum Type***	STANDARD	***Sweep Width (Hz)***	8012.58	***Temperature (degree C)***	39.985

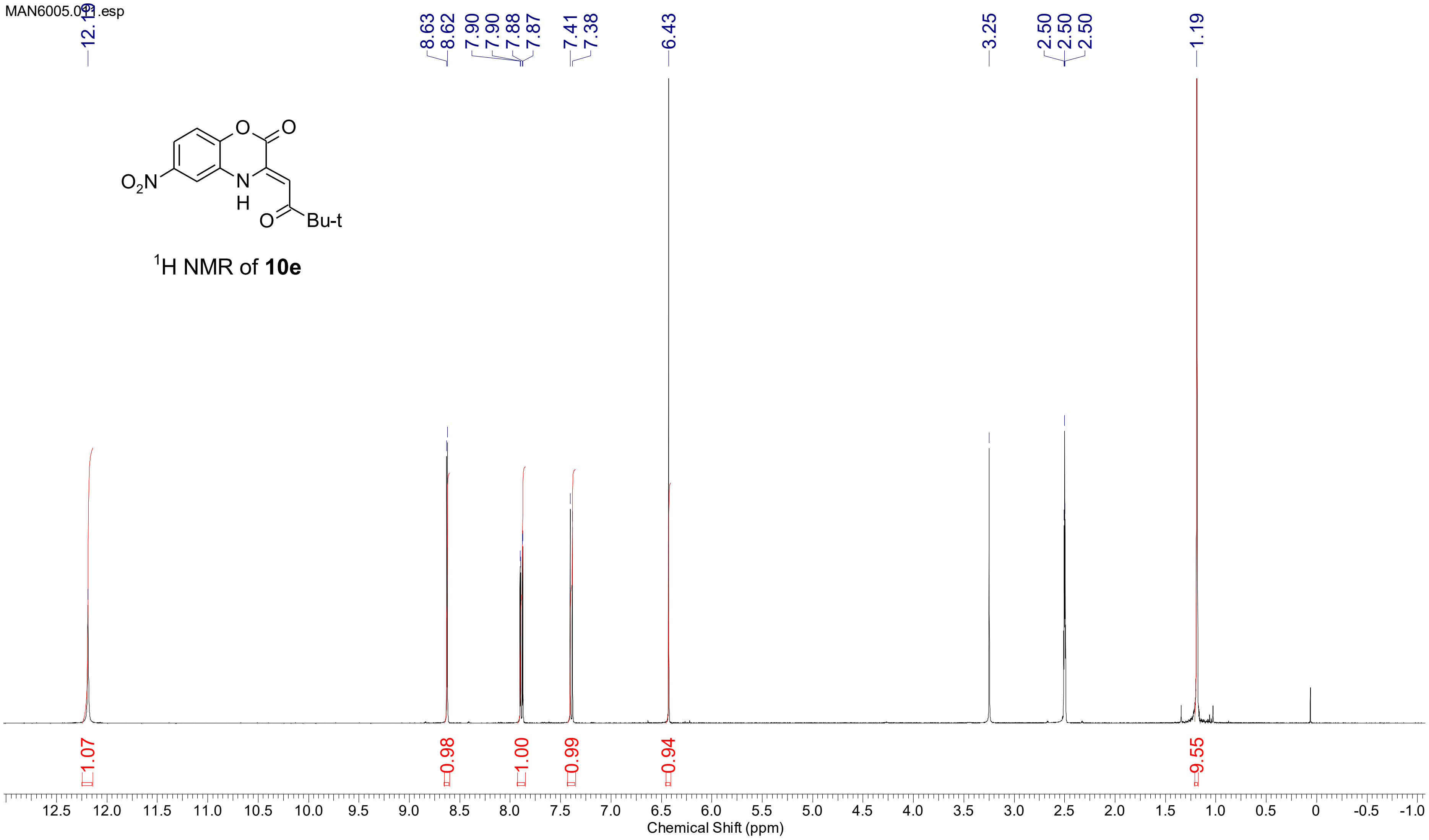

^{1}H NMR of **10e**

Acquisition Time (sec)	2.9999								
Frequency (MHz)	400.17	***Nucleus***	1H	***Number of Transients***	16	***Origin***	spect	***Original Points Count***	24038
Owner	nmr	***Points Count***	32768	***Pulse Sequence***	zg	***Receiver Gain***	47.43	***SW(cyclical) (Hz)***	8012.82
Solvent	DMSO-d6	***Spectrum Offset (Hz)***	2467.9829	***Spectrum Type***	STANDARD	***Sweep Width (Hz)***	8012.58	***Temperature (degree C)***	40.002

MAN5988.011.esp

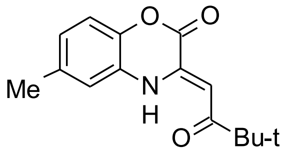

^{1}H NMR of **10f**

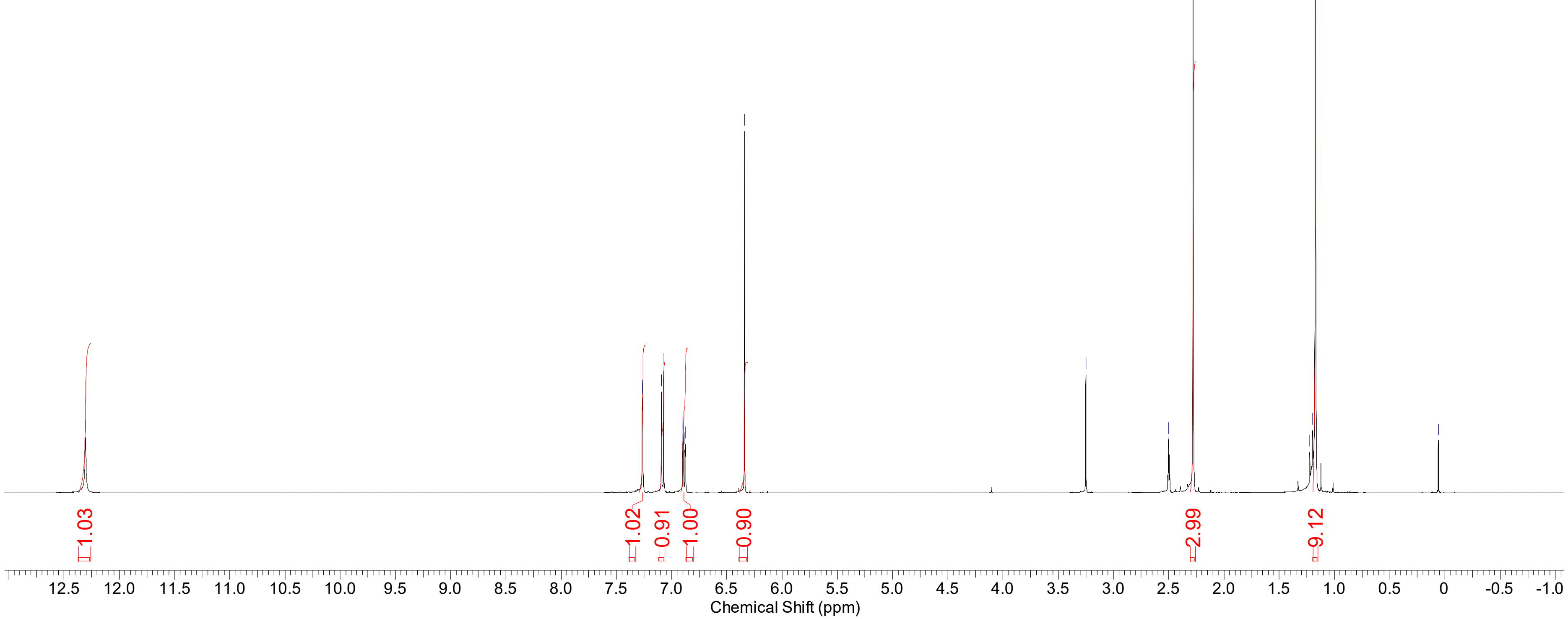

Acquisition Time (sec)	2.9999								
Frequency (MHz)	400.17	***Nucleus***	1H	***Number of Transients***	8	***Origin***	spect	***Original Points Count***	24038
Owner	nmr	***Points Count***	32768	***Pulse Sequence***	zg	***Receiver Gain***	37.99	***SW(cyclical) (Hz)***	8012.82
Solvent	DMSO-d6	***Spectrum Offset (Hz)***	2467.7385	***Spectrum Type***	STANDARD	***Sweep Width (Hz)***	8012.58	***Temperature (degree C)***	40.001

MAN5989.011.esp

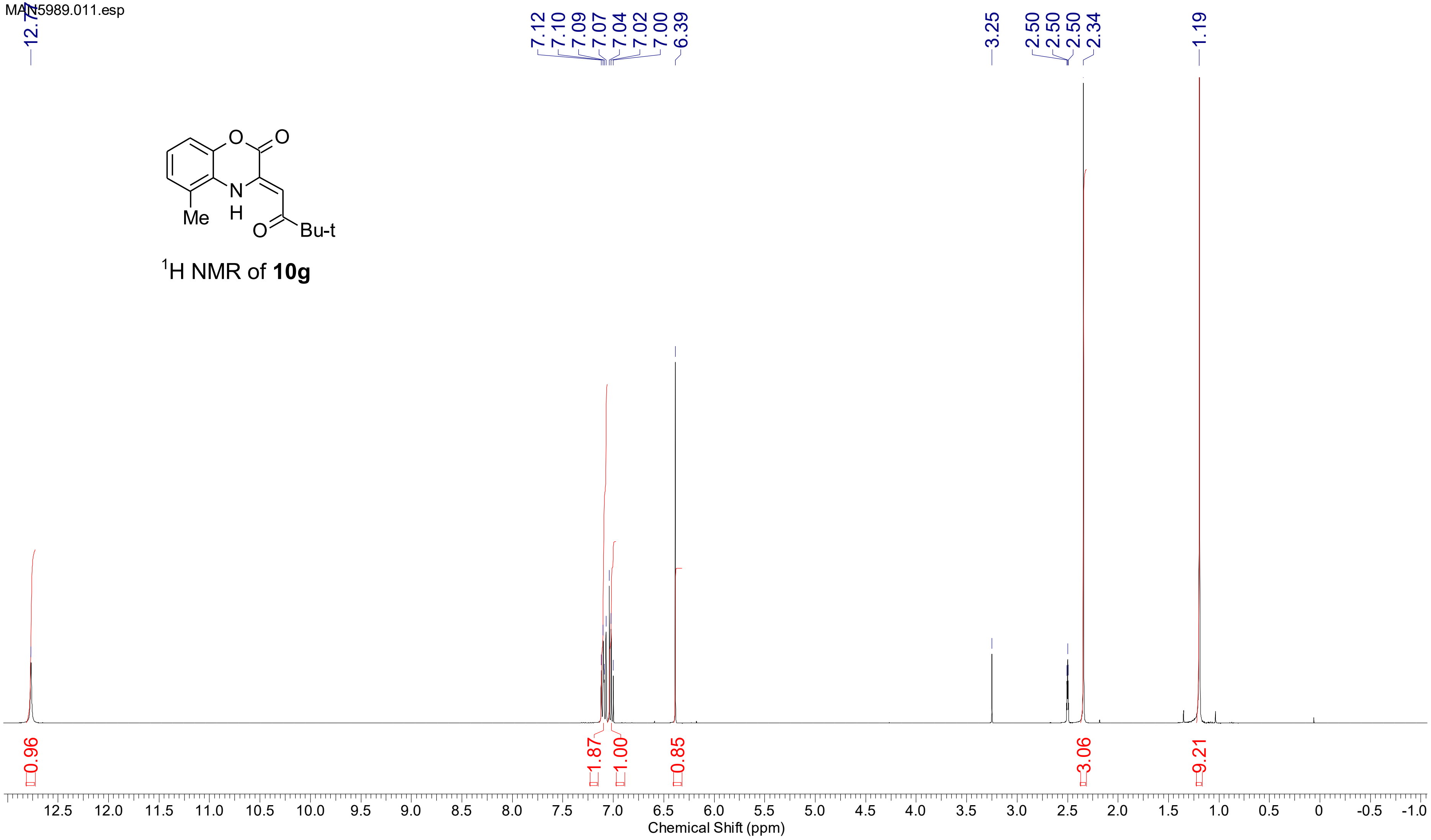

Acquisition Time (sec)	2.9999								
Frequency (MHz)	400.17	***Nucleus***	1H	***Number of Transients***	8	***Origin***	spect	***Original Points Count***	24038
Owner	nmr	***Points Count***	32768	***Pulse Sequence***	zg	***Receiver Gain***	47.43	***SW(cyclical) (Hz)***	8012.82
Solvent	DMSO-d6	***Spectrum Offset (Hz)***	2467.7385	***Spectrum Type***	STANDARD	***Sweep Width (Hz)***	8012.58	***Temperature (degree C)***	40.000

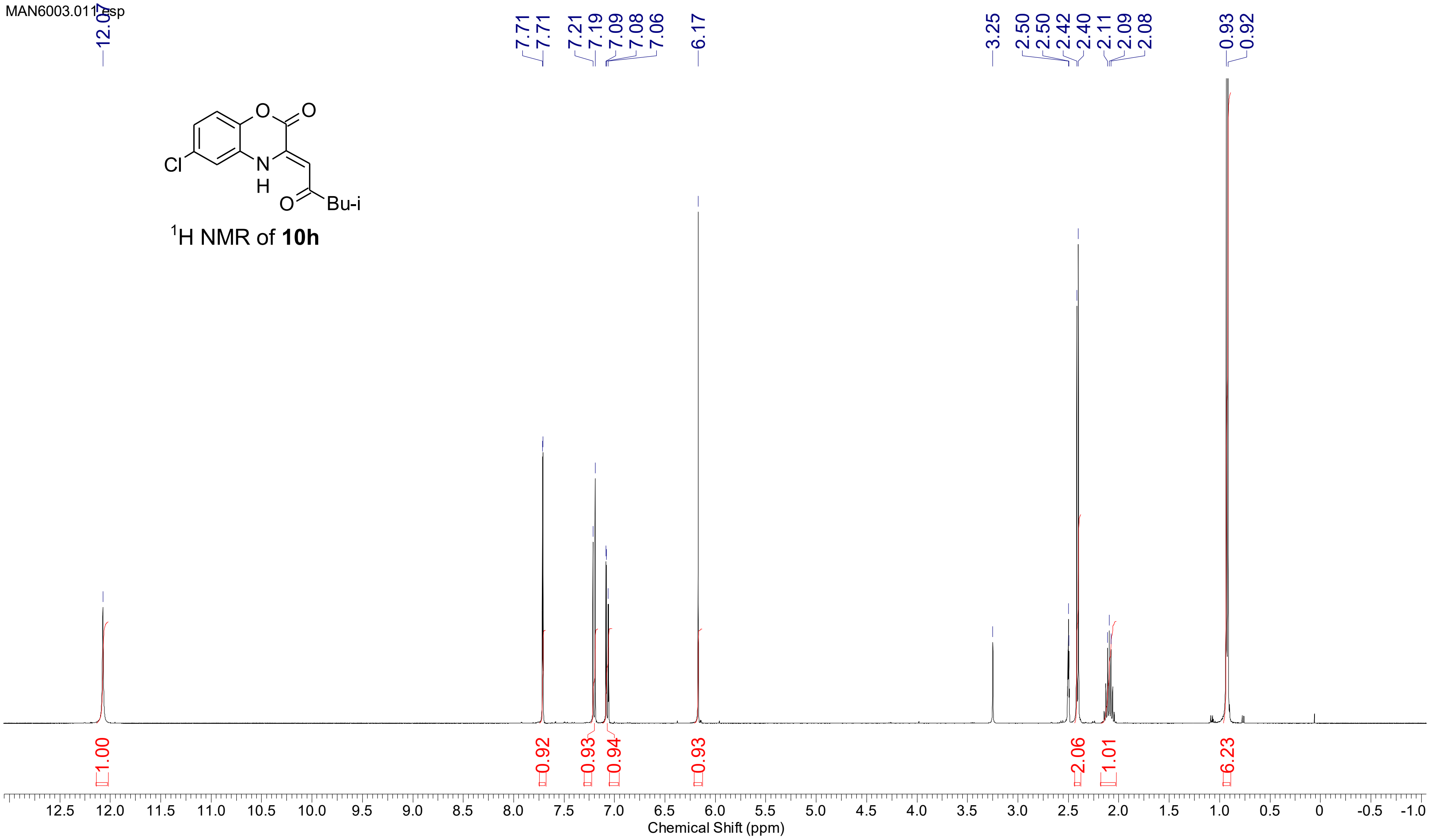

Acquisition Time (sec)	2.9999								
Frequency (MHz)	400.17	***Nucleus***	1H	***Number of Transients***	8	***Origin***	spect	***Original Points Count***	24038
Owner	nmr	***Points Count***	32768	***Pulse Sequence***	zg	***Receiver Gain***	62.95	***SW(cyclical) (Hz)***	8012.82
Solvent	DMSO-d6	***Spectrum Offset (Hz)***	2467.9829	***Spectrum Type***	STANDARD	***Sweep Width (Hz)***	8012.58	***Temperature (degree C)***	39.998

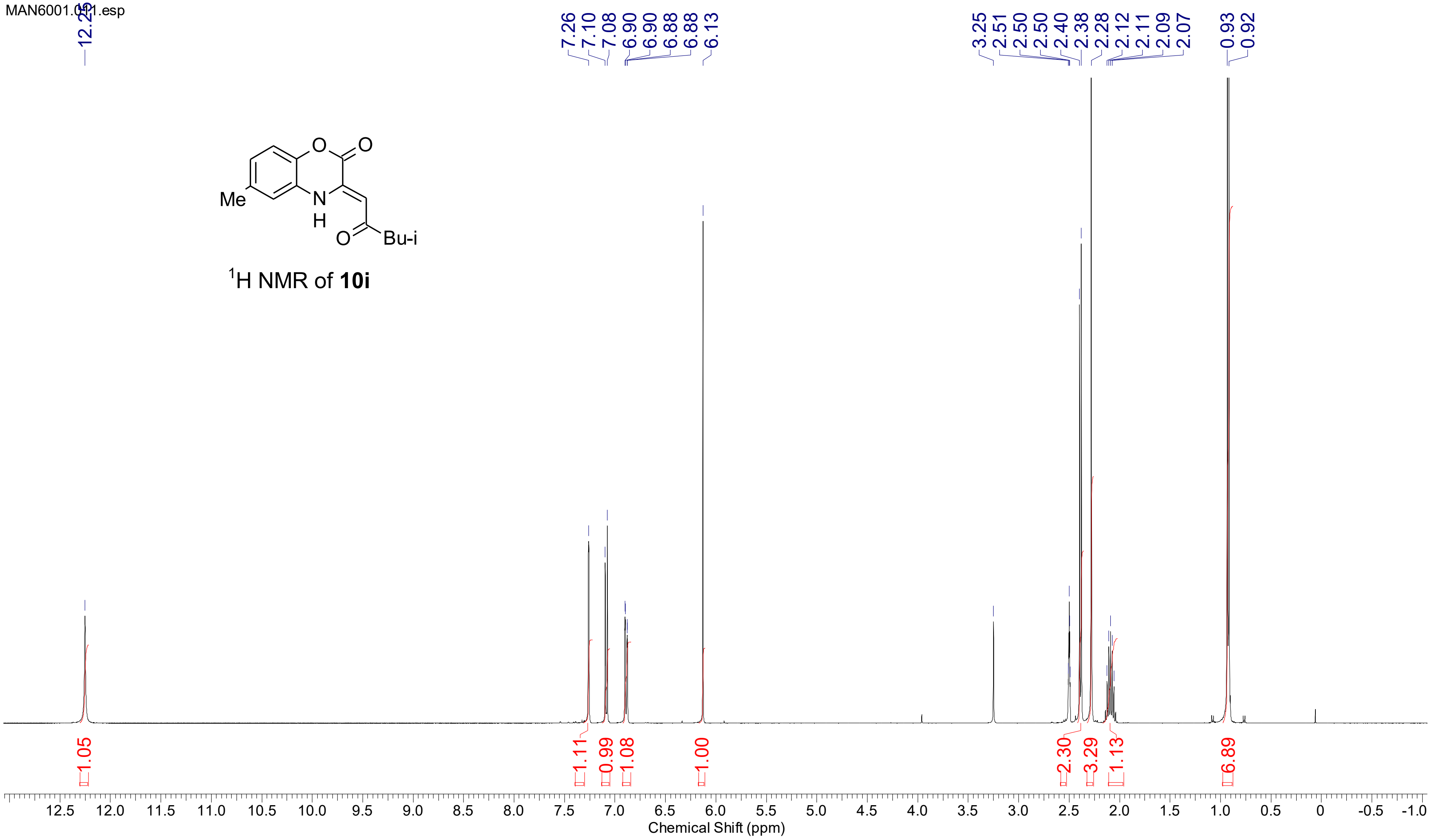

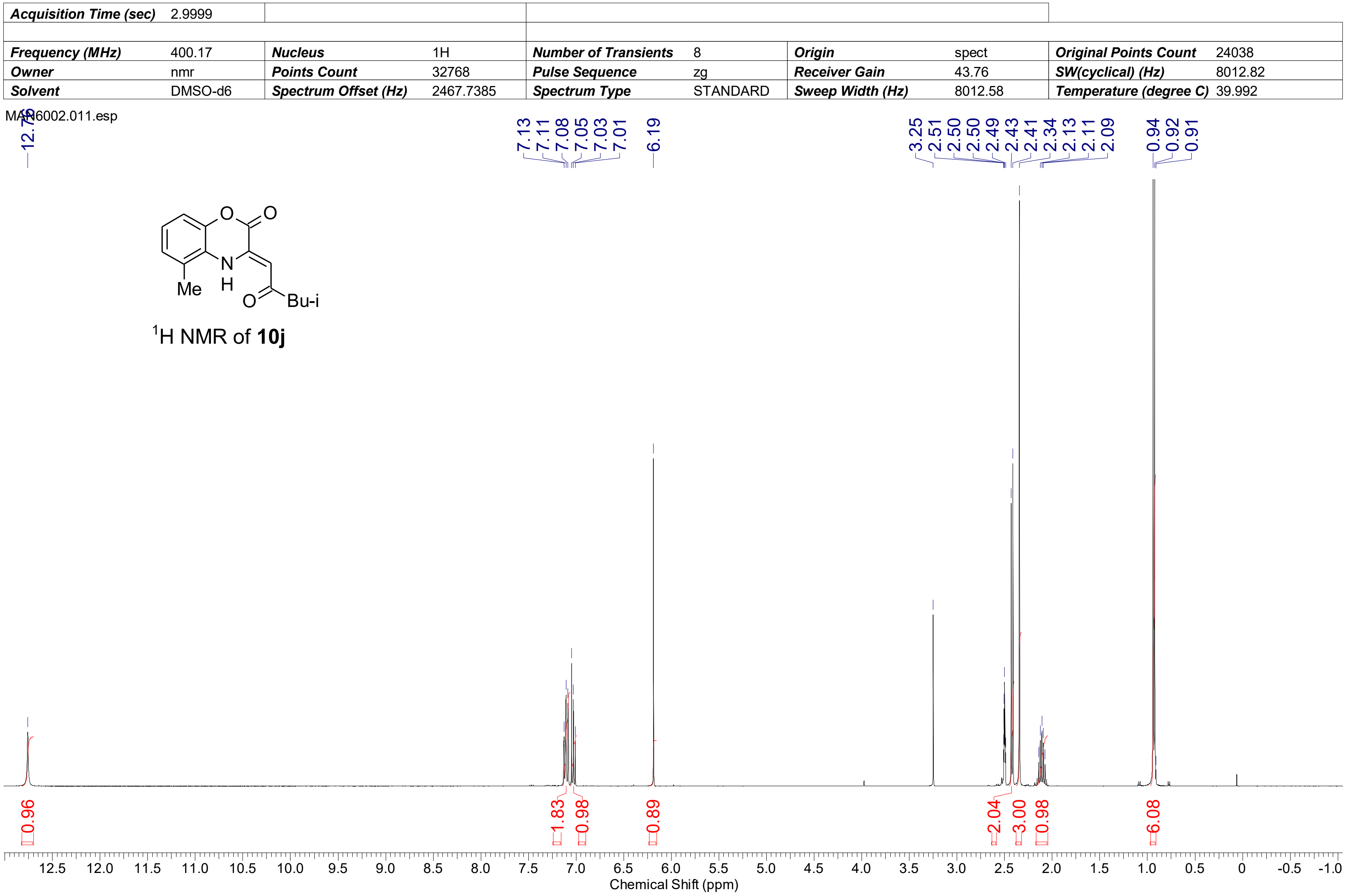

Acquisition Time (sec) 2.9999
Frequency (MHz) 400.17
Nucleus 1H
Number of Transients 8
Origin spect
Original Points Count 24038
Owner nmr
Points Count 32768
Pulse Sequence zg
Receiver Gain 43.76
SW(cyclical) (Hz) 8012.82
Solvent DMSO-d6
Spectrum Offset (Hz) 2467.7385
Spectrum Type STANDARD
Sweep Width (Hz) 8012.58
Temperature (degree C) 39.992
MAN6002.011.esp
12.76
7.13 7.11 7.08 7.05 7.03 7.01
6.19
3.25 2.51 2.50 2.50 2.49 2.43 2.41 2.34 2.13 2.11 2.09
0.94 0.92 0.91
Me
H
N
O
O
O
Bu-i
0.96 1.83 0.98 0.89 2.04 3.00 0.98 6.08
Chemical Shift (ppm)

^{1}H NMR of **10j**

Acquisition Time (sec)	2.9999								
Frequency (MHz)	400.17	***Nucleus***	1H	***Number of Transients***	8	***Origin***	spect	***Original Points Count***	24038
Owner	nmr	***Points Count***	32768	***Pulse Sequence***	zg	***Receiver Gain***	62.95	***SW(cyclical) (Hz)***	8012.82
Solvent	DMSO-d6	***Spectrum Offset (Hz)***	2467.4939	***Spectrum Type***	STANDARD	***Sweep Width (Hz)***	8012.58	***Temperature (degree C)***	39.983

MAN6004.011.esp

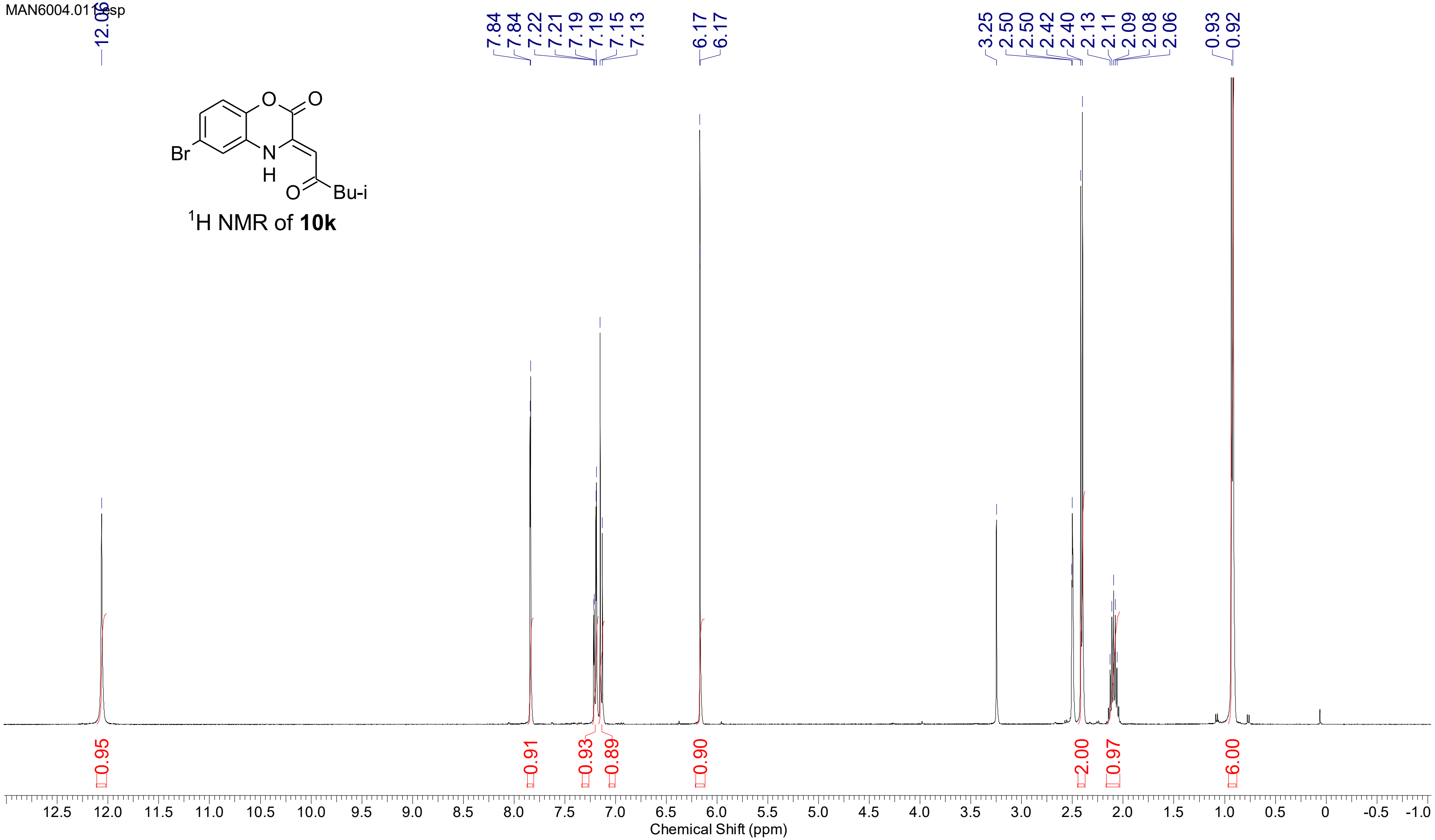

Acquisition Time (sec)	2.9999								
Frequency (MHz)	400.17	***Nucleus***	1H	***Number of Transients***	8	***Origin***	spect	***Original Points Count***	24038
Owner	nmr	***Points Count***	32768	***Pulse Sequence***	zg	***Receiver Gain***	62.95	***SW(cyclical) (Hz)***	8012.82
Solvent	DMSO-d6	***Spectrum Offset (Hz)***	2467.7385	***Spectrum Type***	STANDARD	***Sweep Width (Hz)***	8012.58	***Temperature (degree C)***	40.040

MAN5999.5?1.esp

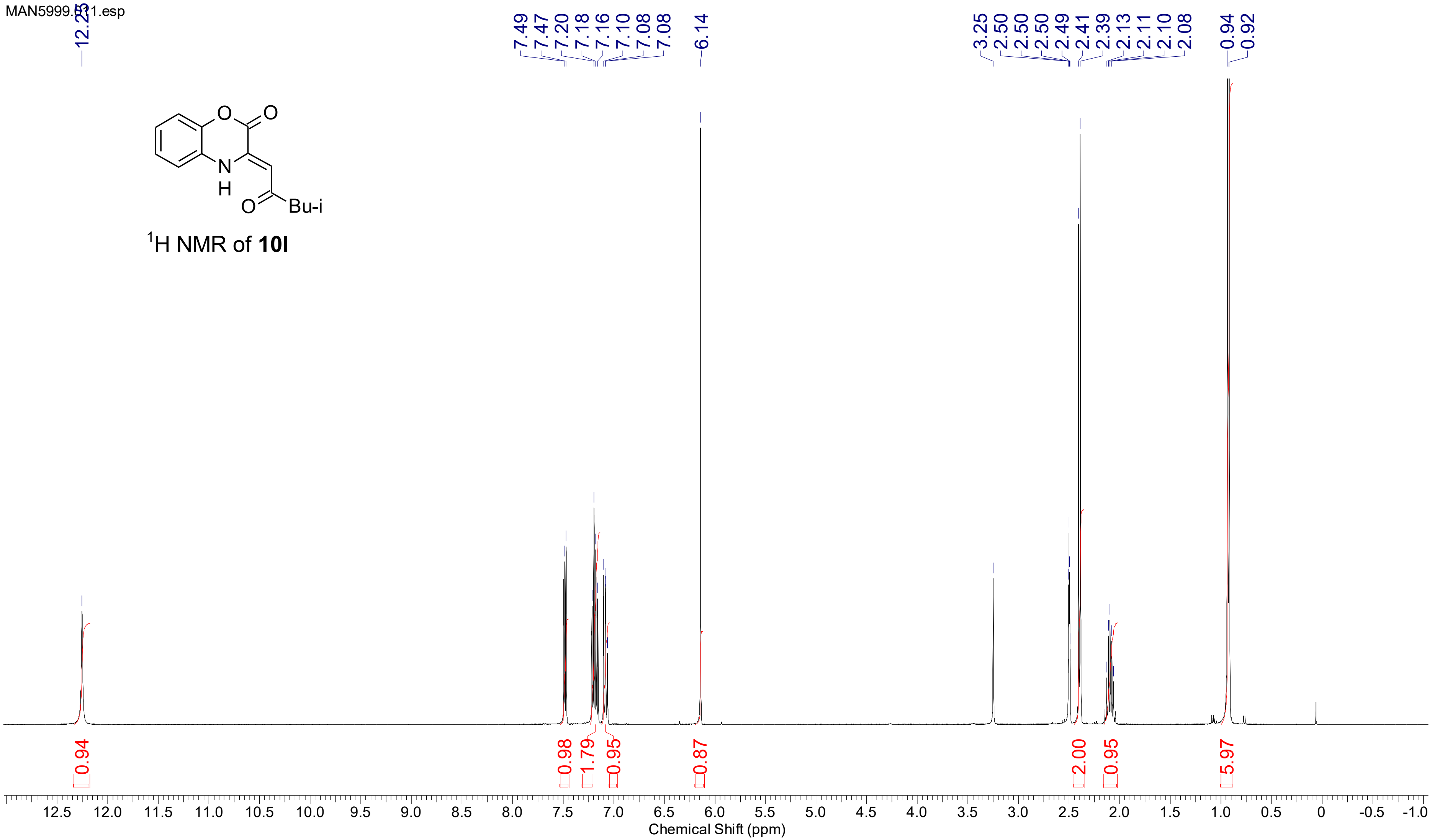

^{1}H NMR of **10l**

Acquisition Time (sec)	2.9295						
Frequency (MHz)	400.13	Nucleus	1H	Number of Transients	16	Origin	drx400
Original Points Count	16384	Owner	uralnmr	Points Count	16384	Pulse Sequence	zg30
Receiver Gain	256.00	SW(cyclical) (Hz)	5592.84	Solvent	CHLOROFORM-d		
Spectrum Offset (Hz)	2391.7268	Spectrum Type	STANDARD	Sweep Width (Hz)	5592.50	Temperature (degree C)	25.000

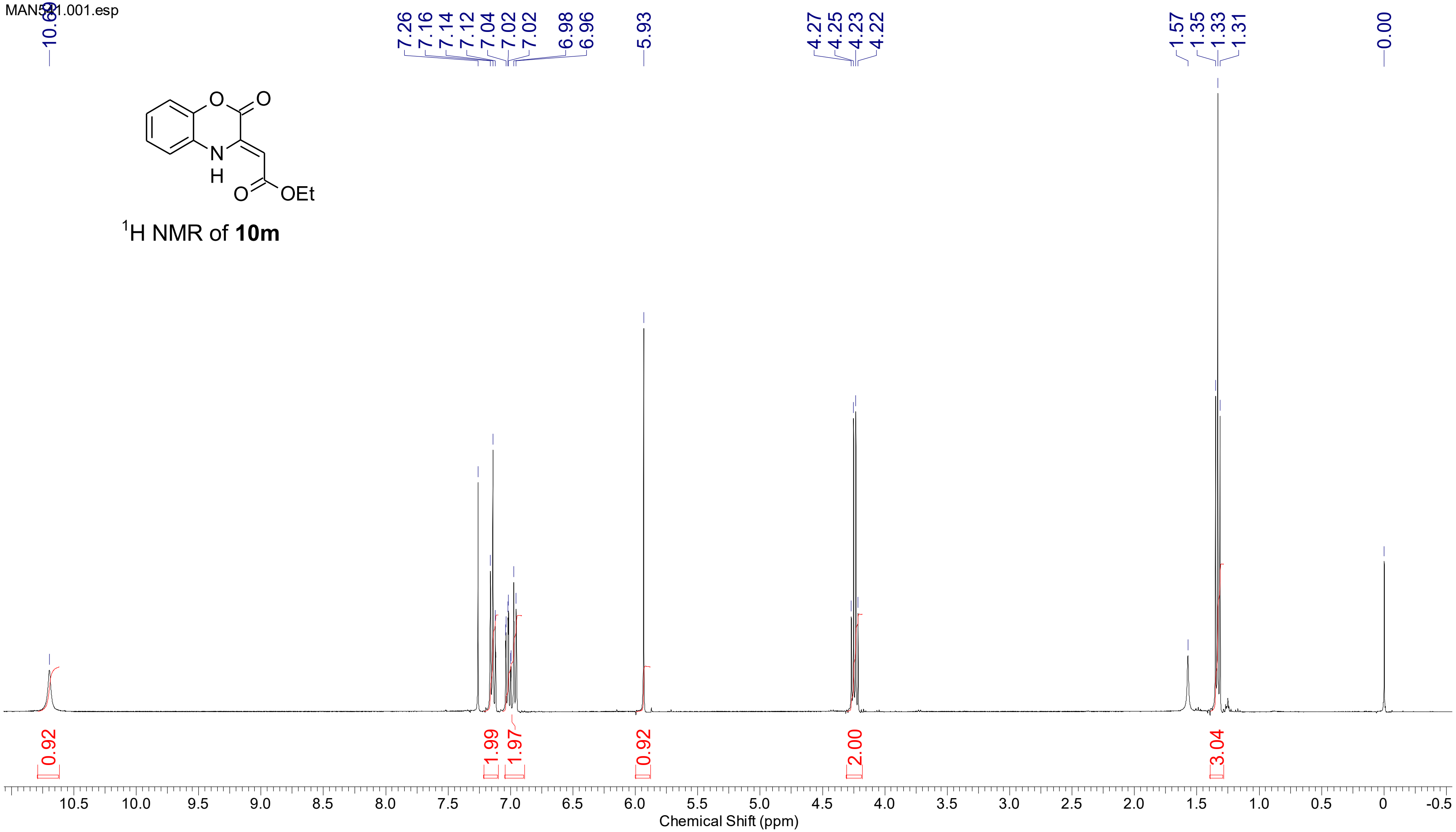

Acquisition Time (sec)	2.9999								
						Frequency (MHz)	400.17	***Nucleus***	1H
Number of Transients	8	***Origin***	spect	***Original Points Count***	24038	***Owner***	nmr	***Points Count***	32768
Pulse Sequence	zg	***Receiver Gain***	77.64	***SW(cyclical) (Hz)***	8012.82	***Solvent***	DMSO-d6	***Spectrum Offset (Hz)***	2467.7385
Spectrum Type	STANDARD	***Sweep Width (Hz)***	8012.58	***Temperature (degree C)***	39.978				

MAN5332.001.esp

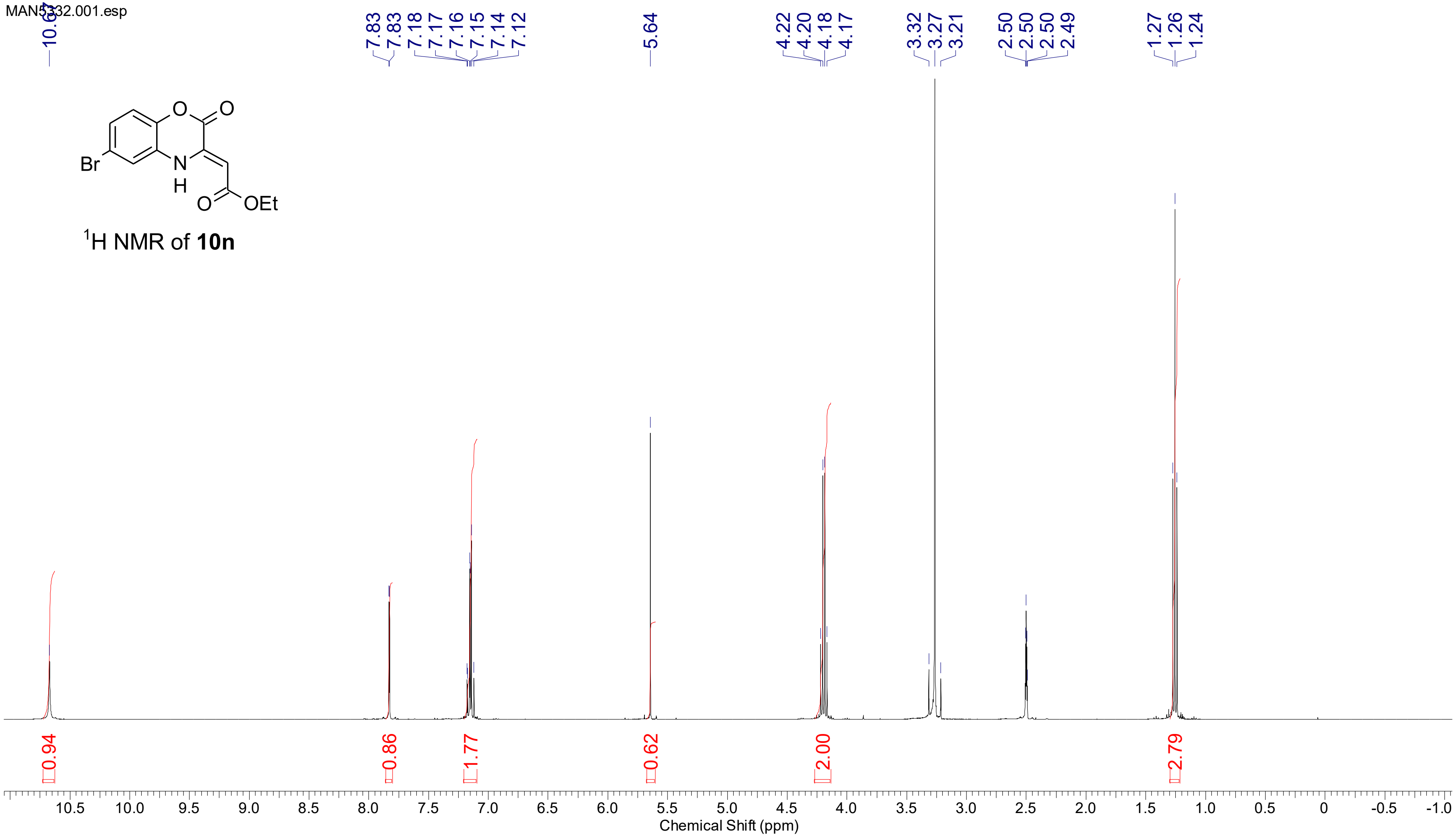

^{1}H NMR of **10n**

Acquisition Time (sec)	2.9999								
Frequency (MHz)	400.17	Nucleus	1H	Number of Transients	32	Origin	spect	Original Points Count	24038
Owner	nmr	Points Count	32768	Pulse Sequence	zg	Receiver Gain	87.04	SW(cyclical) (Hz)	8012.82
Solvent	DMSO-d6	Spectrum Offset (Hz)	2467.7385	Spectrum Type	STANDARD	Sweep Width (Hz)	8012.58	Temperature (degree C)	39.999

MAN5934.001.esp

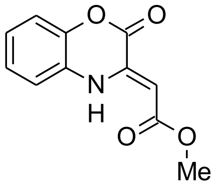

^{1}H NMR of **10o**

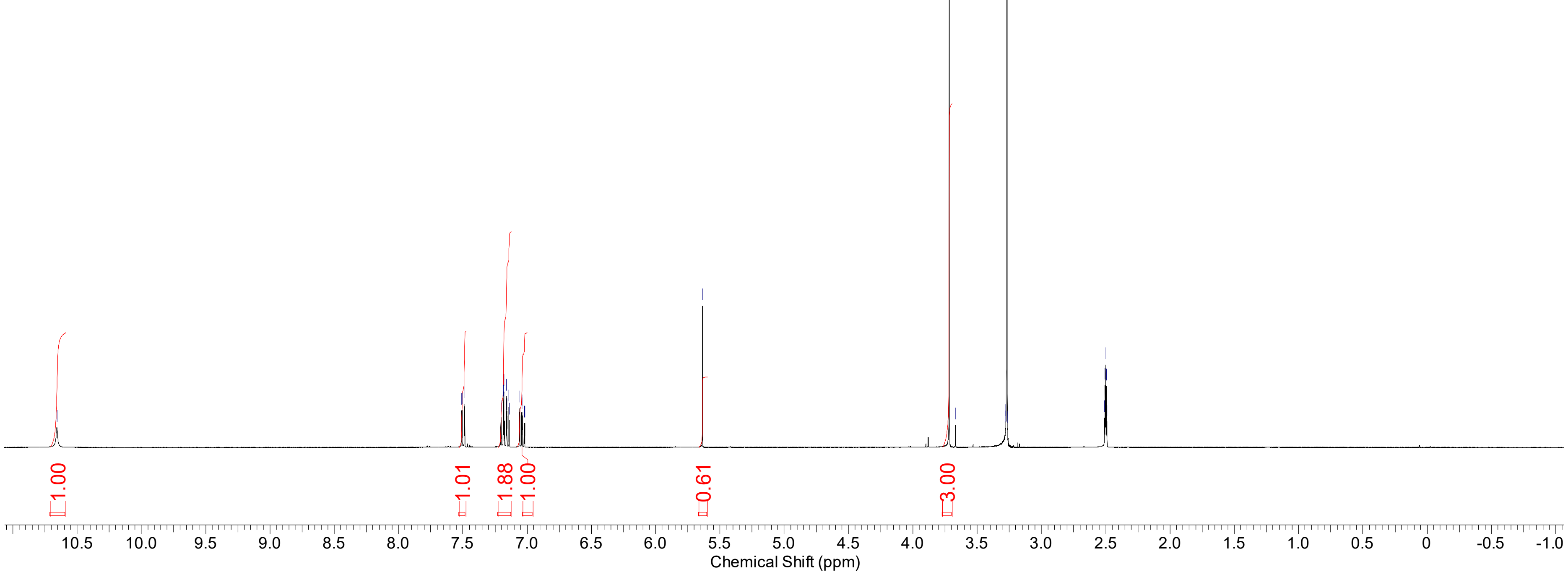

Acquisition Time (sec)	2.0447								
						Frequency (MHz)	400.17	***Nucleus***	1H
Number of Transients	8	***Origin***	spect	***Original Points Count***	16384	***Owner***	nmr	***Points Count***	16384
Pulse Sequence	zg	***Receiver Gain***	95.56	***SW(cyclical) (Hz)***	8012.82	***Solvent***	DMSO-d6	***Spectrum Offset (Hz)***	2467.3718
Spectrum Type	STANDARD	***Sweep Width (Hz)***	8012.33	***Temperature (degree C)***	39.999				

MAN9327.001.esp

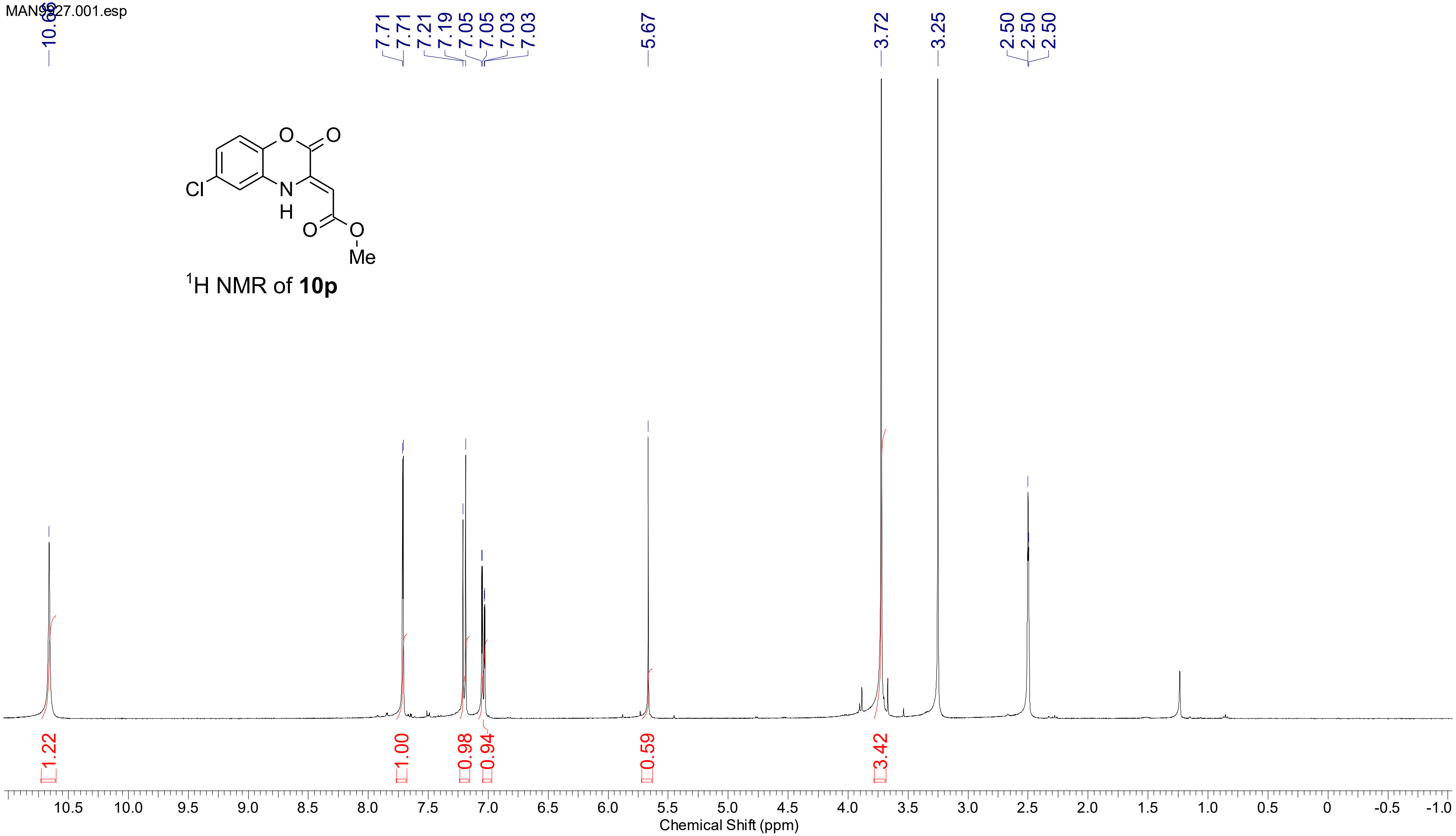

^{1}H NMR of **10p**

Acquisition Time (sec)	2.0447								
						Frequency (MHz)	400.17	***Nucleus***	1H
Number of Transients	8	***Origin***	spect	***Original Points Count***	16384	***Owner***	nmr	***Points Count***	16384
Pulse Sequence	zg	***Receiver Gain***	87.04	***SW(cyclical) (Hz)***	8012.82	***Solvent***	DMSO-d6	***Spectrum Offset (Hz)***	2467.8608
Spectrum Type	STANDARD	***Sweep Width (Hz)***	8012.33	***Temperature (degree C)***	39.996				

MAN9928.001.esp

^{1}H NMR of **10q**

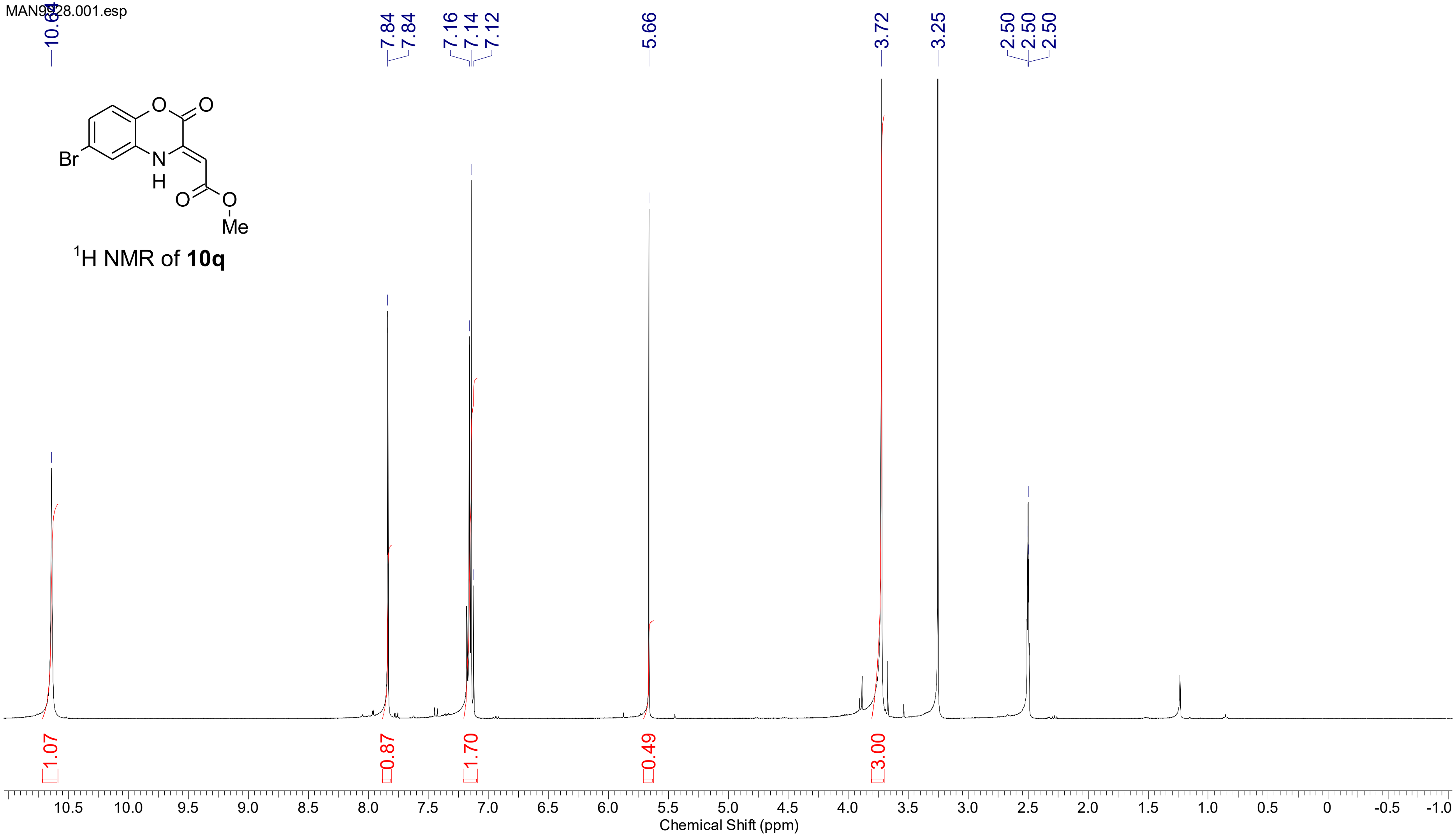

Acquisition Time (sec)	1.2976								
Frequency (MHz)	100.62	***Nucleus***	13C	***Number of Transients***	512	***Origin***	spect	***Original Points Count***	32768
Owner	nmr	***Points Count***	32768	***Pulse Sequence***	zgpg30	***Receiver Gain***	196.95	***SW(cyclical) (Hz)***	25252.53
Solvent	DMSO-d6	***Spectrum Offset (Hz)***	11510.5977	***Spectrum Type***	STANDARD	***Sweep Width (Hz)***	25251.75	***Temperature (degree C)***	40.003

MAN9928.002.esp

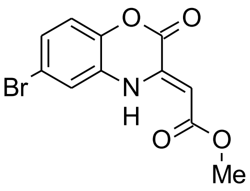

^{13}C NMR of **10q**

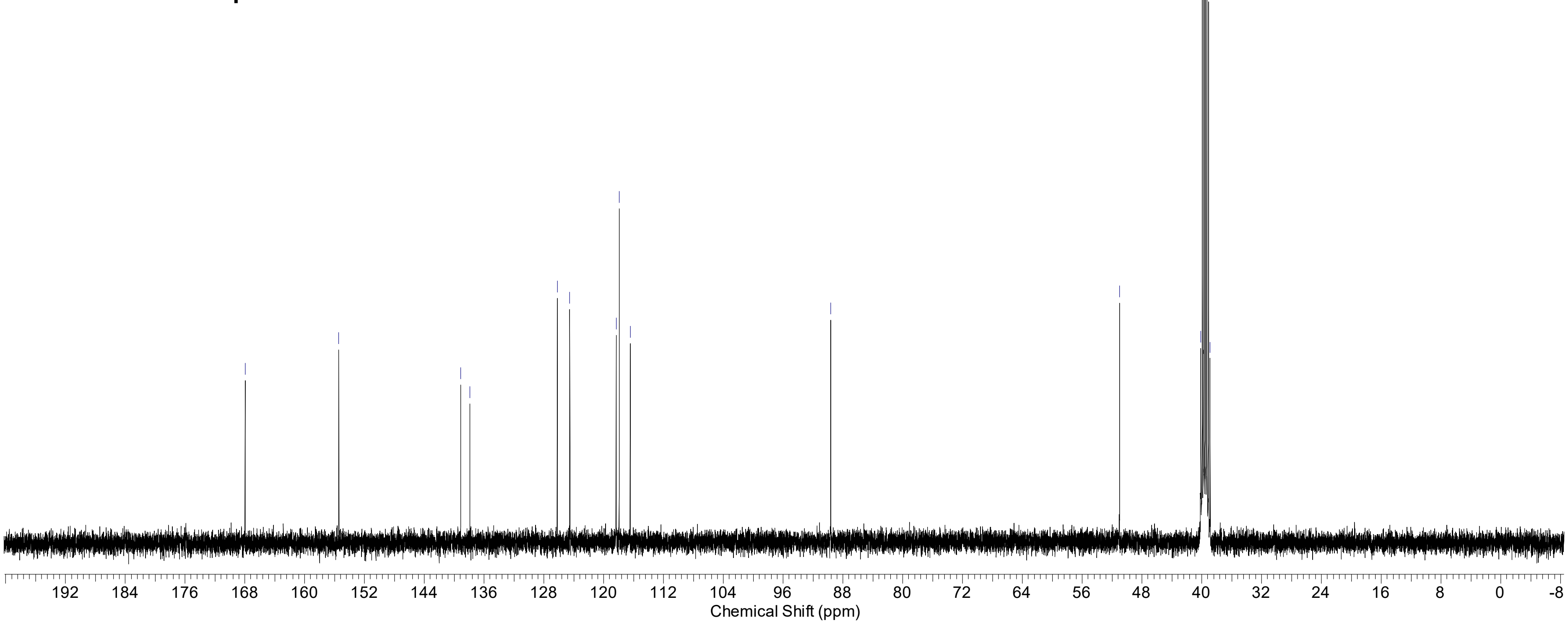

NMR chart of compound 11

Acquisition Time (sec)	2.9999								
Frequency (MHz)	400.17	***Nucleus***	1H	***Number of Transients***	8	***Origin***	spect	***Original Points Count***	24038
Owner	nmr	***Points Count***	32768	***Pulse Sequence***	zg	***Receiver Gain***	37.99	***SW(cyclical) (Hz)***	8012.82
Solvent	DMSO-d6	***Spectrum Offset (Hz)***	2467.9829	***Spectrum Type***	STANDARD	***Sweep Width (Hz)***	8012.58	***Temperature (degree C)***	40.001

MA35659.001.esp

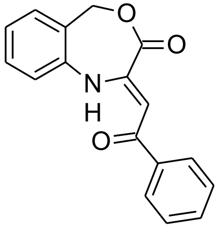

^{1}H NMR of **11**

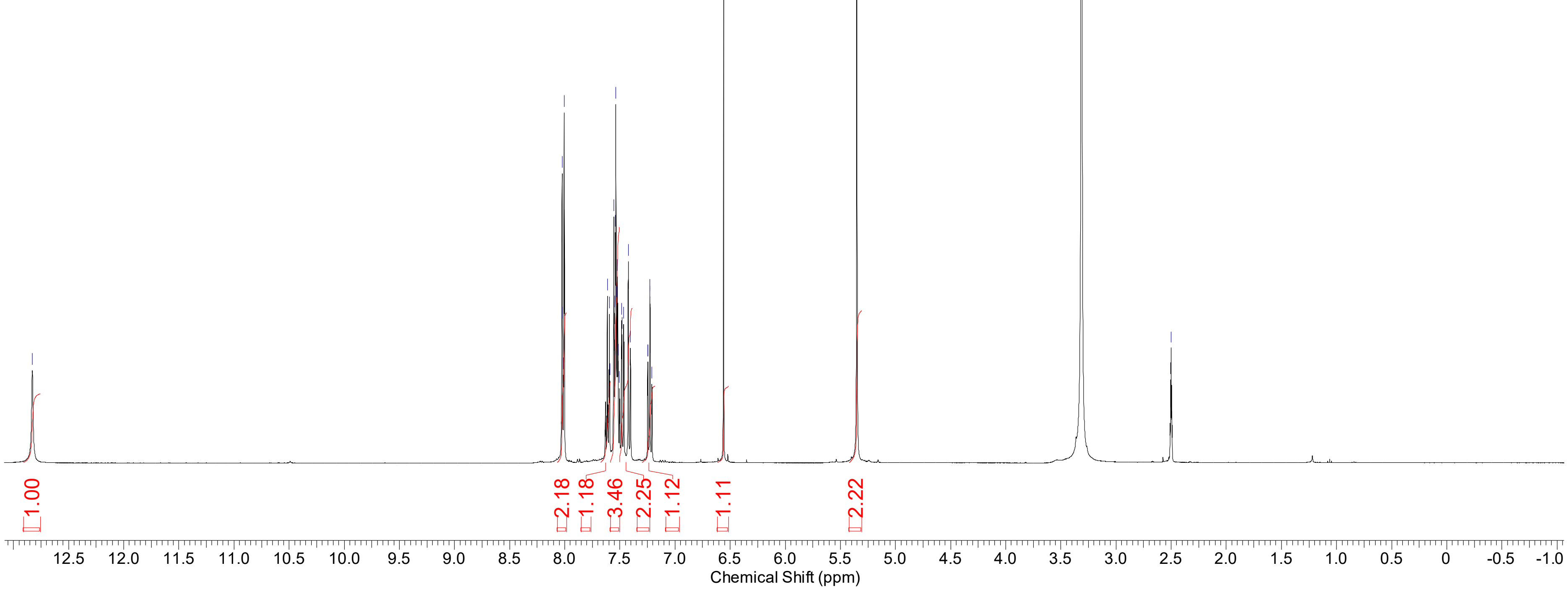

NMR chart of compound 13a

Acquisition Time (sec)	8.1789						
				Frequency (MHz)	400.17	*Nucleus*	1H
Number of Transients	1	*Origin*	spect	*Original Points Count*	65536	*Owner*	nmr
Points Count	65536						
Pulse Sequence	zg	*Receiver Gain*	35.09	*SW(cyclical) (Hz)*	8012.82	*Solvent*	DMSO-d6
Spectrum Offset (Hz)	2467.9219						
Spectrum Type	STANDARD	*Sweep Width (Hz)*	8012.70	*Temperature (degree C)*	39.996		

MAN6522.001.esp

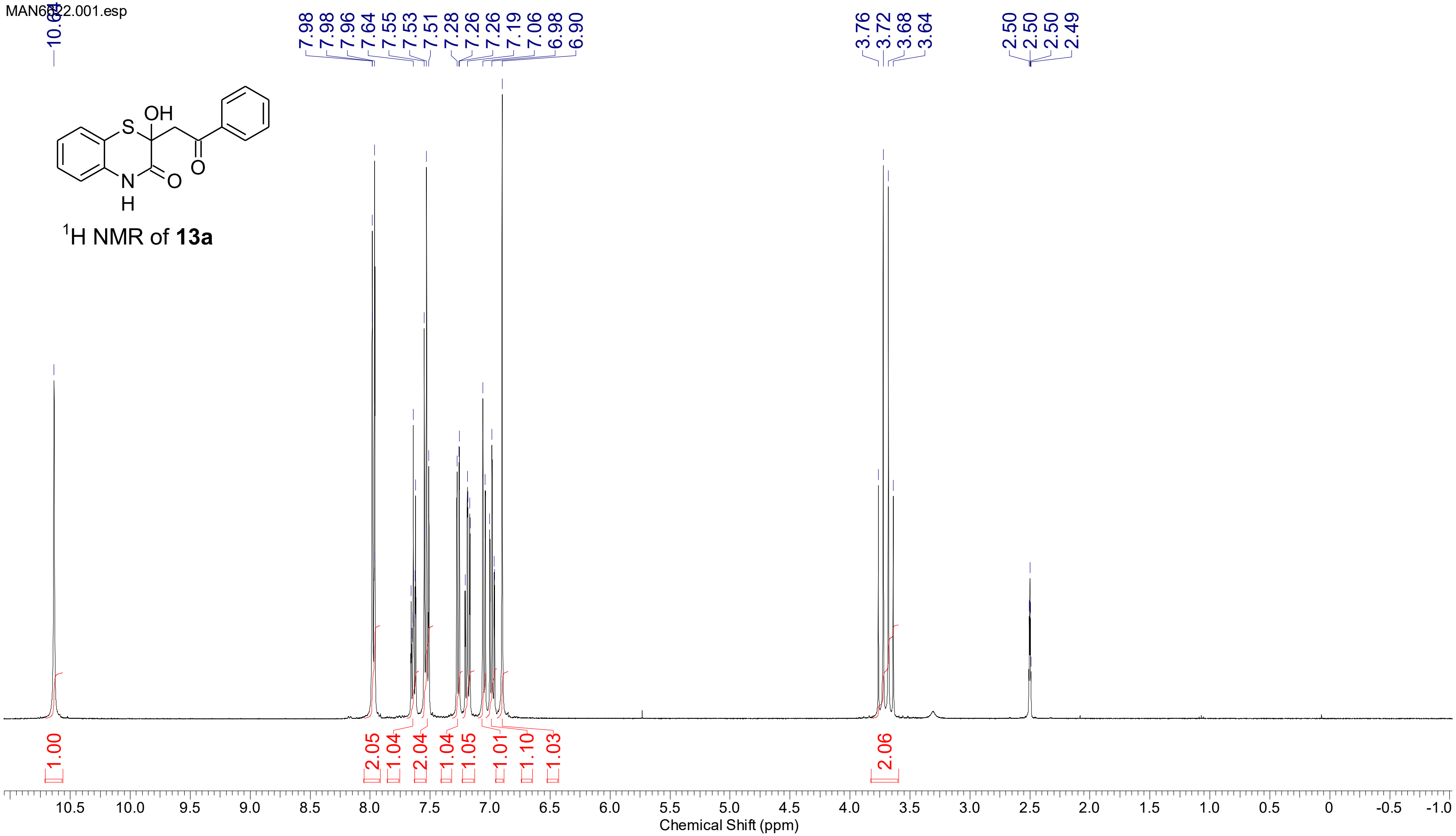

NMR charts of compounds 14

Acquisition Time (sec)	4.9999								
Frequency (MHz)	400.17	***Nucleus***	1H	***Number of Transients***	32	***Origin***	spect	***Original Points Count***	40063
Owner	nmr	***Points Count***	65536	***Pulse Sequence***	zg	***Receiver Gain***	87.04	***SW(cyclical) (Hz)***	8012.82
Solvent	DMSO-d6	***Spectrum Offset (Hz)***	2467.9219	***Spectrum Type***	STANDARD	***Sweep Width (Hz)***	8012.70	***Temperature (degree C)***	40.001

MAN5076.001.esp

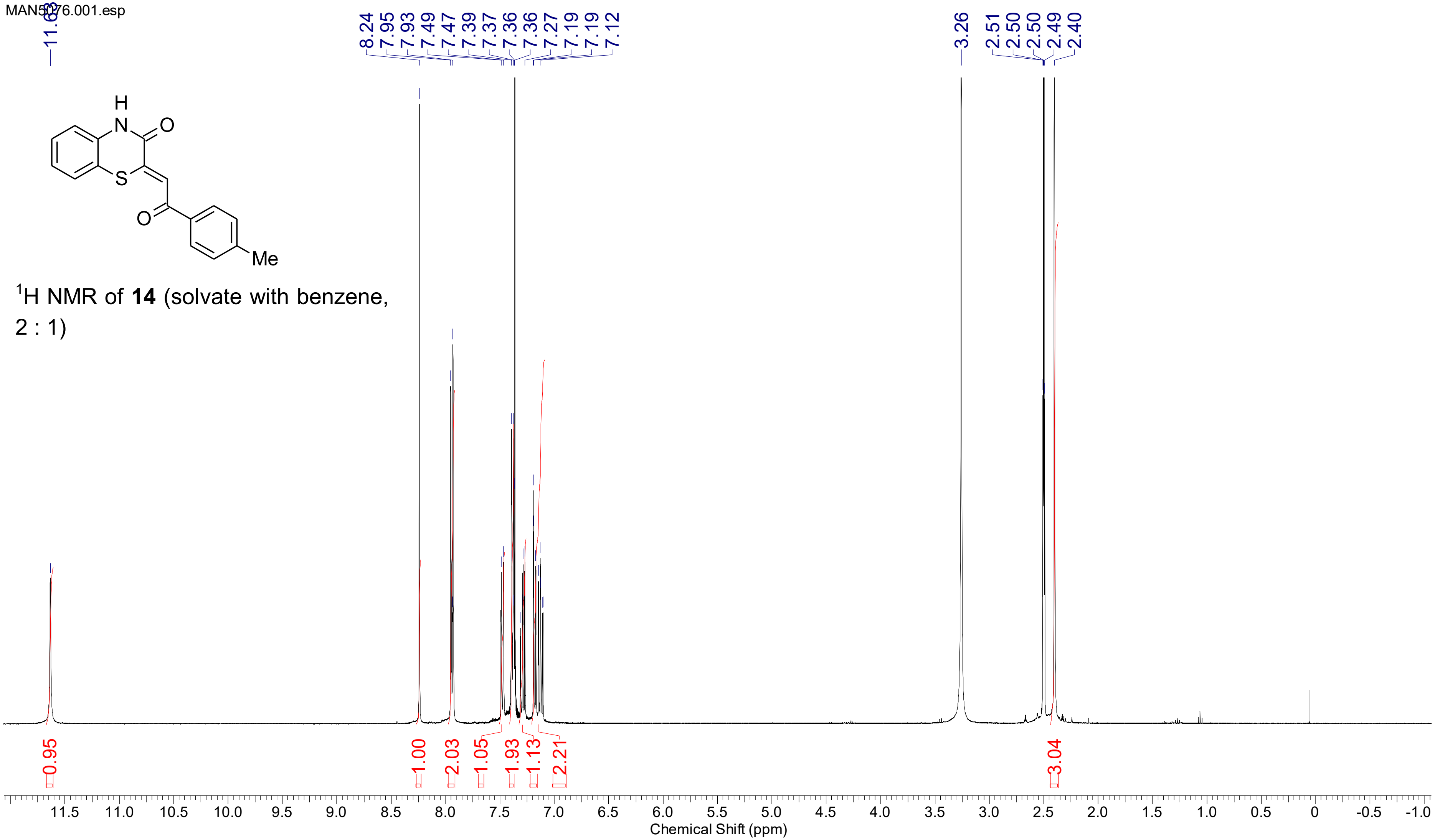

^{1}H NMR of **14** (solvate with benzene, 2 : 1)

Acquisition Time (sec)	1.2976						
Frequency (MHz)	100.62	Nucleus	13C	Number of Transients	1024	Origin	spect
Original Points Count	32768	Owner	nmr	Points Count	1048576	Pulse Sequence	zgpg30
Receiver Gain	196.95	SW(cyclical) (Hz)	25252.53	Solvent	DMSO-d6	Spectrum Offset (Hz)	11510.8535
Spectrum Type	STANDARD	Sweep Width (Hz)	25252.50	Temperature (degree C)	40.004		

MAN5076.002.esp

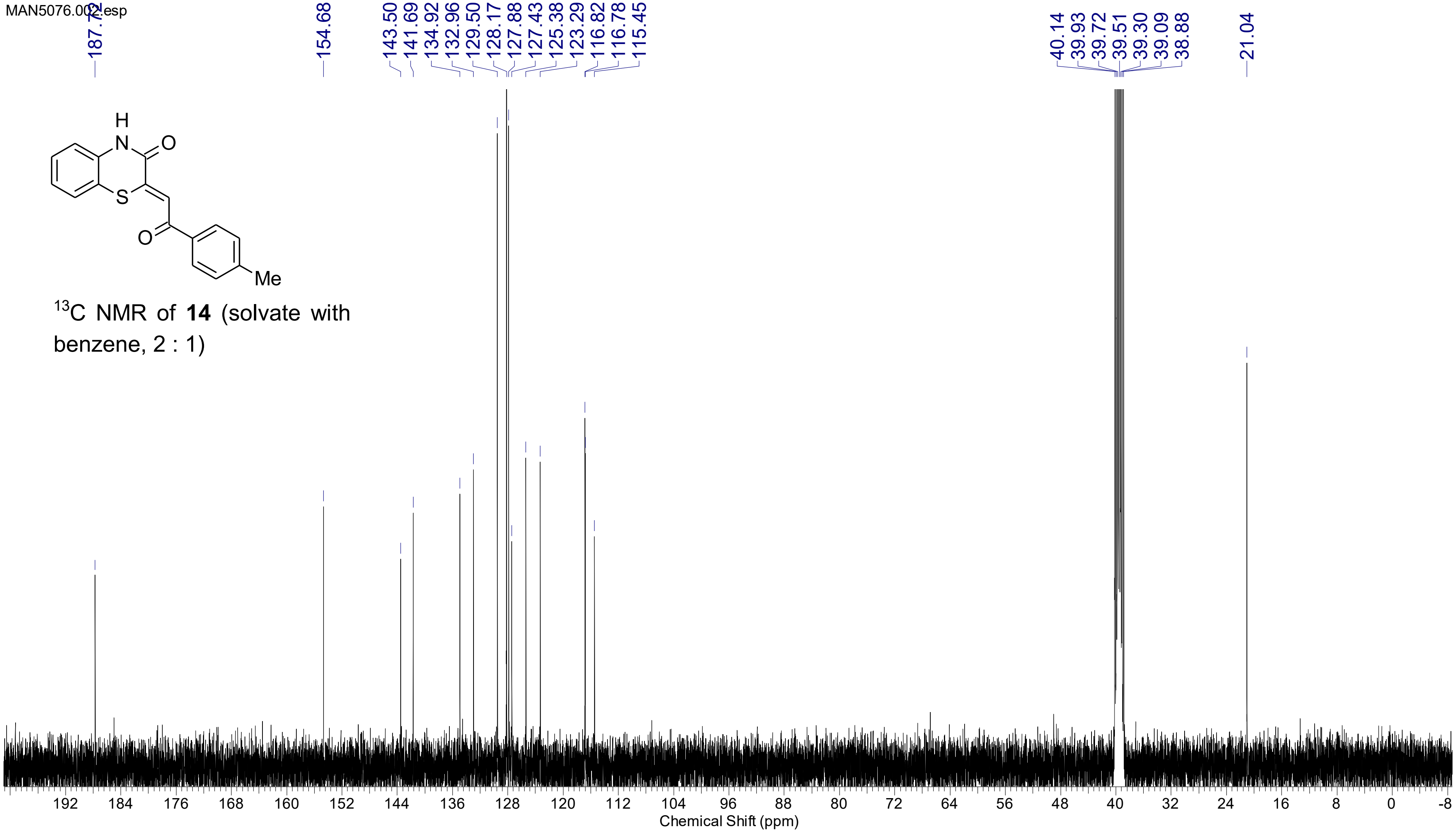

^{13}C NMR of **14** (solvate with benzene, 2 : 1)

NMR chart of compound 15

Acquisition Time (sec)	2.9999								
Frequency (MHz)	400.17	***Nucleus***	1H	***Number of Transients***	8	***Origin***	spect	***Original Points Count***	24038
Owner	nmr	***Points Count***	32768	***Pulse Sequence***	zg	***Receiver Gain***	47.43	***SW(cyclical) (Hz)***	8012.82
Solvent	DMSO-d6	***Spectrum Offset (Hz)***	2467.7385	***Spectrum Type***	STANDARD	***Sweep Width (Hz)***	8012.58	***Temperature (degree C)***	40.005

MAN5889.001.esp

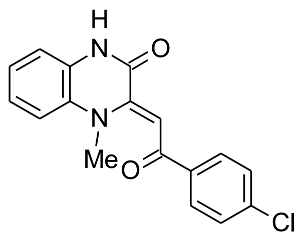

^{1}H NMR of **15**

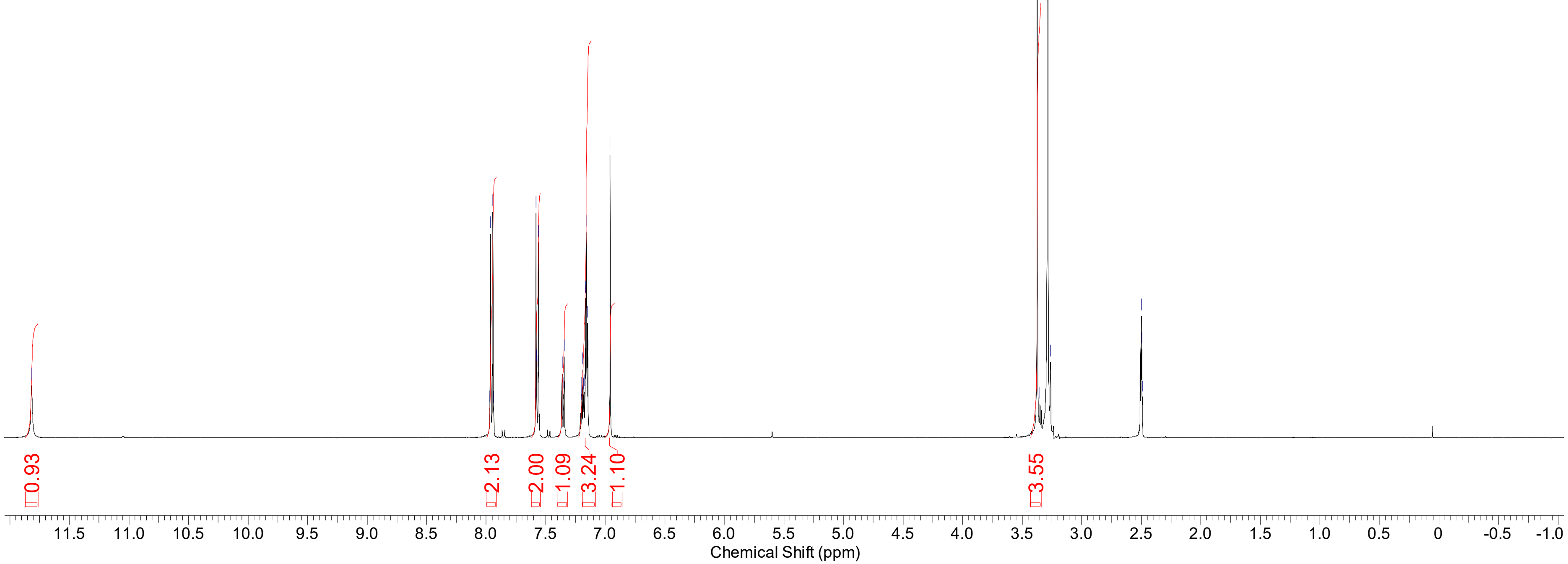

NMR chart of compound 16

Acquisition Time (sec)	2.9999								
Frequency (MHz)	400.17	Nucleus	1H	Number of Transients	32	Origin	spect	Original Points Count	24038
Owner	nmr	Points Count	32768	Pulse Sequence	zg	Receiver Gain	62.95	SW(cyclical) (Hz)	8012.82
Solvent	DMSO-d6	Spectrum Offset (Hz)	2467.9829	Spectrum Type	STANDARD	Sweep Width (Hz)	8012.58	Temperature (degree C)	40.000

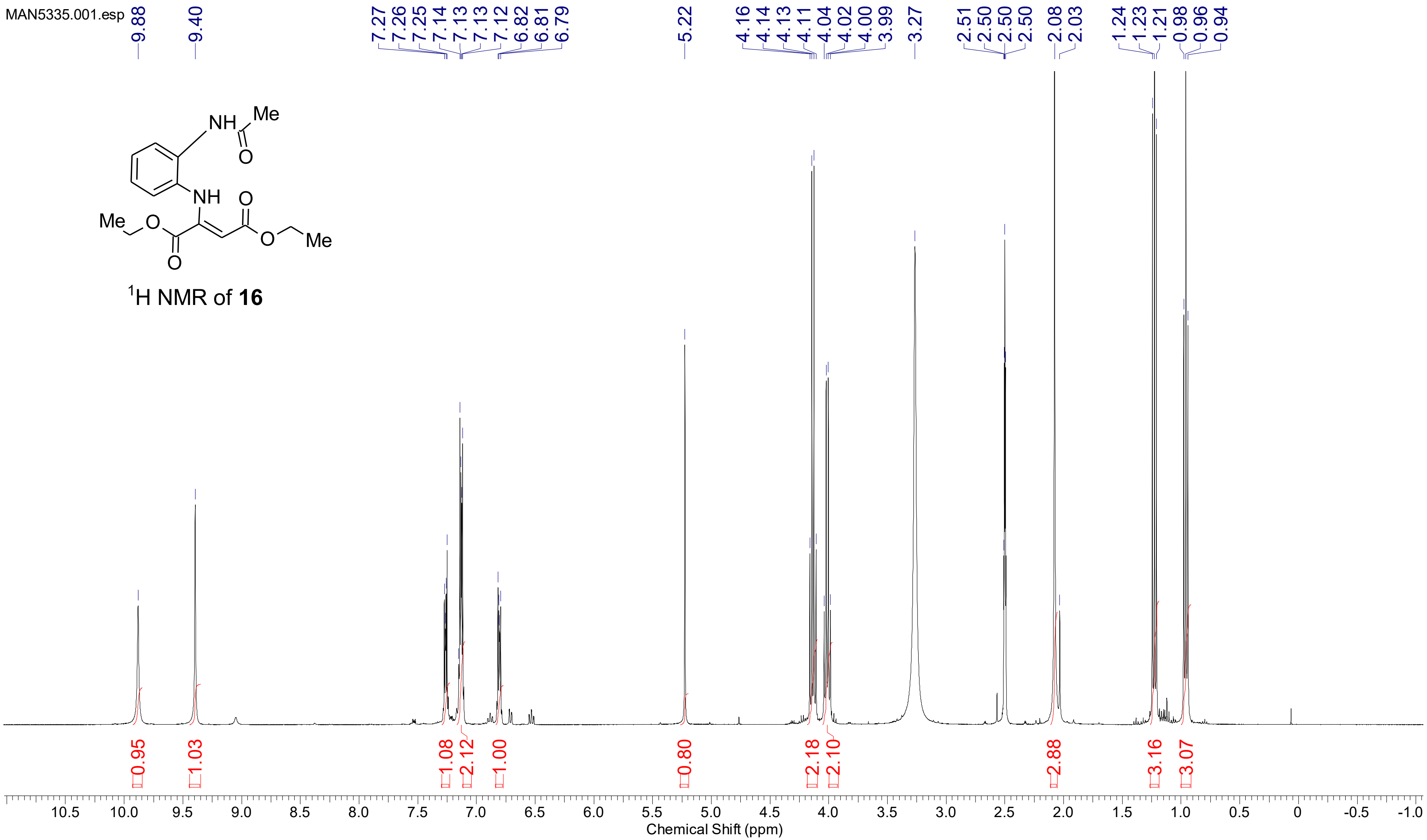

^{1}H NMR of **16**

ORTEP images of X-ray crystal structures 2n, 6f, 14

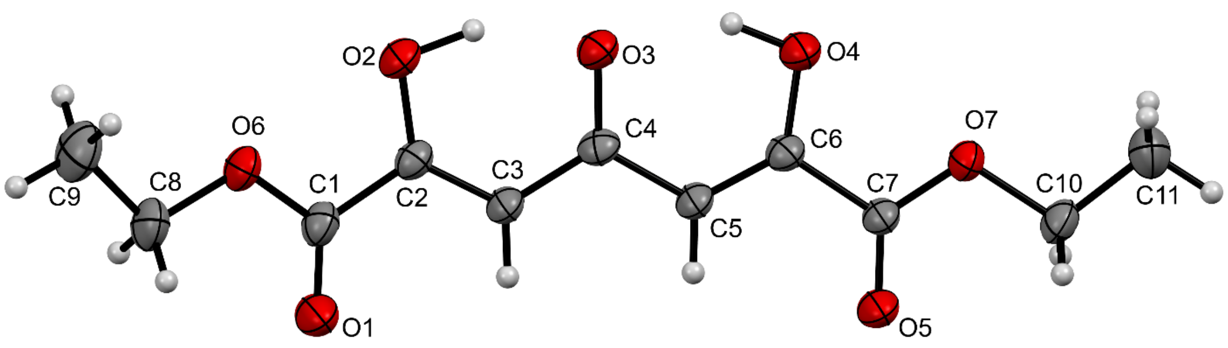

Figure S1. *Molecular structure of compound* ***2n*** *showing 30% probability amplitude displacement ellipsoids (CCDC 2486572).*

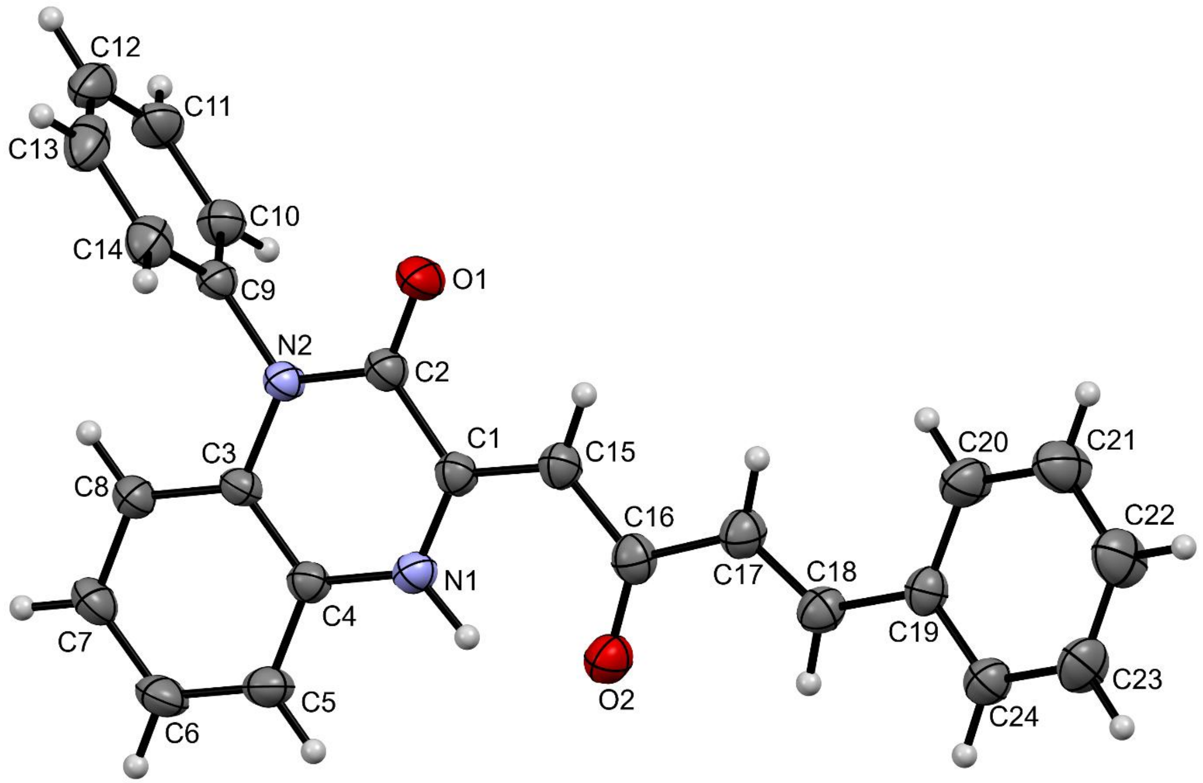

Figure S2. *Molecular structure of compound **6f** showing 30% probability amplitude displacement ellipsoids (CCDC 2010681; Refcode ZURBOG). Only one independent molecule is shown.*

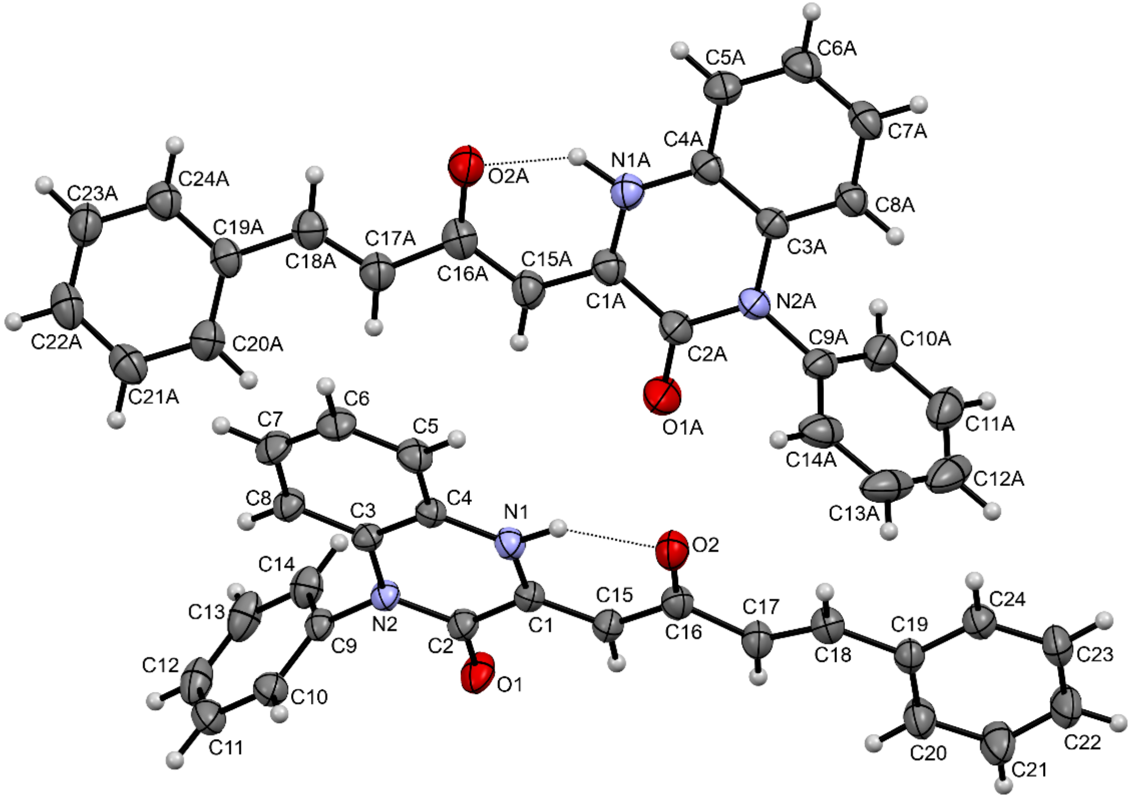

Figure S3. *The asymmetric unit of compound* ***6f*** *showing 30% probability amplitude displacement ellipsoids (CCDC 2010681; Refcode ZURBOG).*

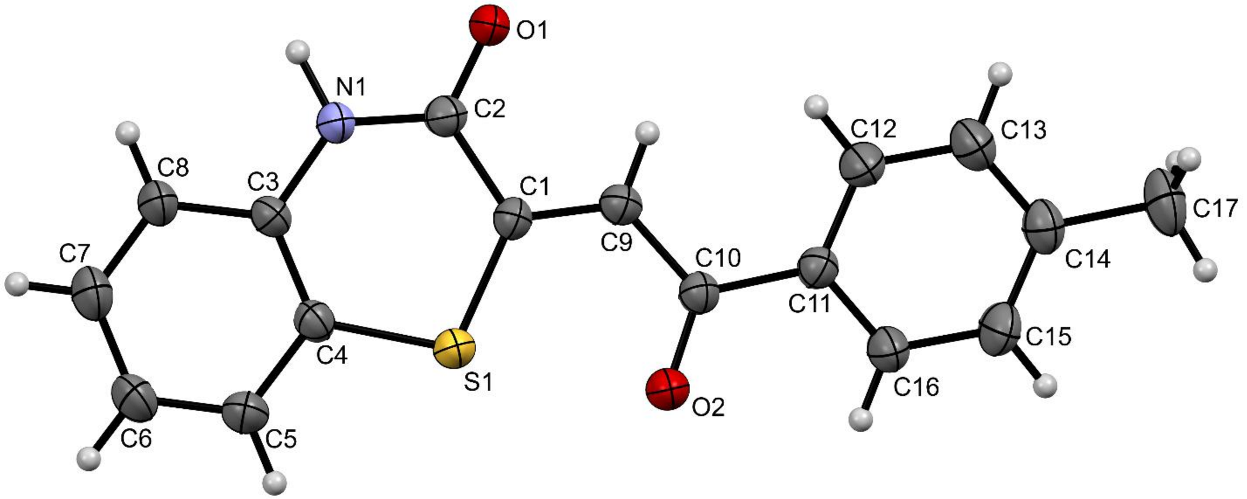

Figure S4. *Molecular structure of compound* ***14*** *showing 30% probability amplitude displacement ellipsoids (CCDC 2486573). Only one independent molecule is shown. Benzene solvate molecule is not shown.*

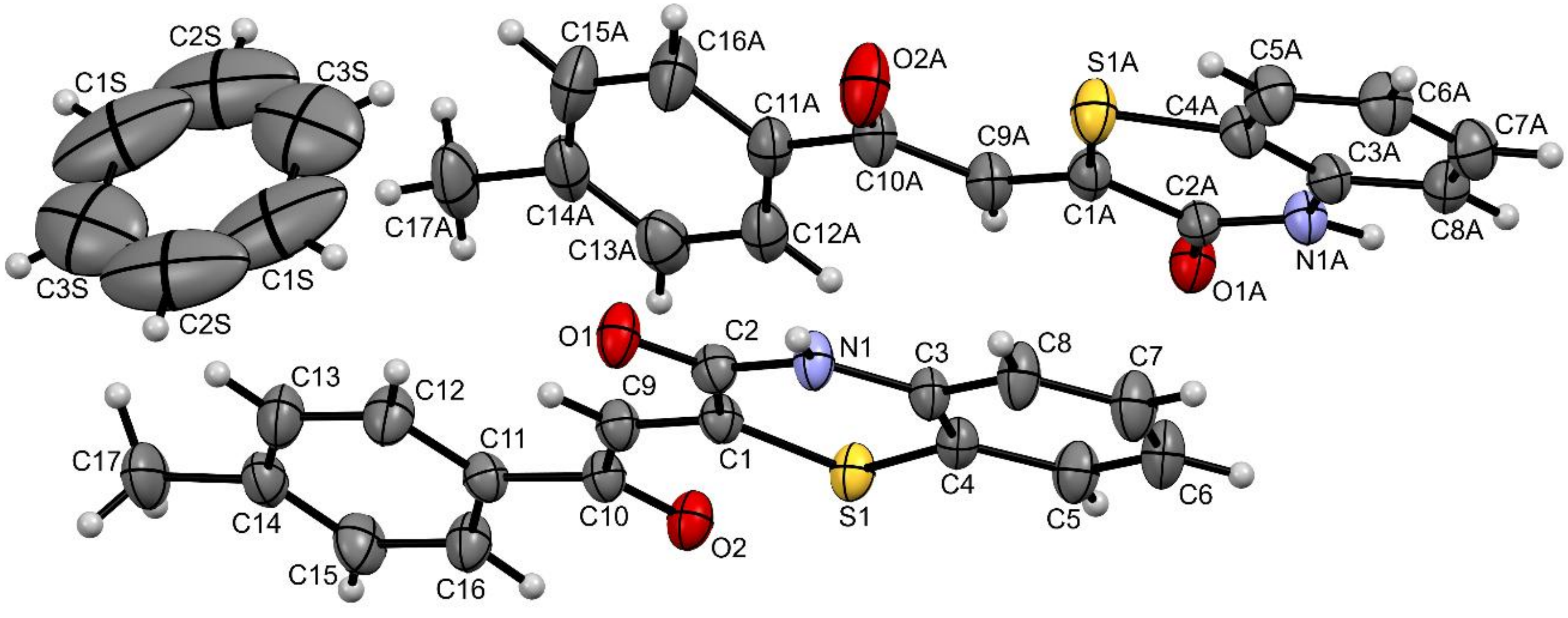

Figure S5. *The asymmetric unit of compound 1**4** showing 30% probability amplitude displacement ellipsoids (CCDC 2486573). Benzene solvate molecule is shown in its entirety.*
